# Dual Emissive Zn(II) Naphthalocyanines: Synthesis, Structural and Photophysical Characterization with Theory-Supported Insights towards Soluble Coordination Compounds with Visible and Near-Infrared Emission

**DOI:** 10.3390/ijms25052605

**Published:** 2024-02-23

**Authors:** Sidharth Thulaseedharan Nair Sailaja, Iván Maisuls, Alexander Hepp, Dana Brünink, Nikos L. Doltsinis, Andreas Faust, Sven Hermann, Cristian A. Strassert

**Affiliations:** 1 Institut für Anorganische und Analytische Chemie, Universität Münster, Corrensstraße 28/30, 48149 Münster, Germanymaisuls@uni-muenster.de (I.M.); alexander.hepp@uni-muenster.de (A.H.); 2 CeNTech, CiMIC, SoN, Universität Münster, Heisenbergstraße 11, 48149 Münster, Germany; 3 Institute for Solid State Theory and Center for Multiscale Theory and Computation, Universität Münster, Wilhelm-Klemm-Straße 10, 48149 Münster, Germany; 4 European Institute for Molecular Imaging, Universität Münster, Röntgenstraße 16, 48149 Münster, Germany; faustan@uni-muenster.de (A.F.);; 5 Department of Nuclear Medicine, University Hospital Münster, Albert-Schweitzer-Campus 1, 48149 Münster, Germany

**Keywords:** dual fluorescence, multiscale–multimodal imaging, photoacoustic/optoacoustic spectroscopy, steady-state and time-resolved multiphoton micro(spectro)scopy (FLIM), (TD-)DFT

## Abstract

Metal phthalocyaninates and their higher homologues are recognized as deep-red luminophores emitting from their lowest excited singlet state. Herein, we report on the design, synthesis, and in-depth characterization of a new class of dual-emissive (visible and NIR) metal naphthalocyaninates. A 4-*N*,*N*-dimethylaminophen-4-yl-substituted naphthalocyaninato zinc(II) complex (**Zn-NMe_2_Nc**) and the derived water-soluble coordination compound (**Zn-NMe_3_Nc**) exhibit a near-infrared fluorescence from the lowest ligand-centered state, along with a unique push–pull-supported luminescence in the visible region of the electromagnetic spectrum. An unprecedentedly broad structural (2D-NMR spectroscopy and mass spectrometry) as well as photophysical characterization (steady-state state and time-resolved photoluminescence spectroscopy) is presented. The unique dual emission was assigned to two independent sets of singlet states related to the intrinsic Q-band of the macrocycle and to the push–pull substituents in the molecular periphery, respectively, as predicted by TD-DFT calculations. In general, the elusive chemical aspects of these macrocyclic compounds are addressed, involving both reaction conditions, thorough purification, and in-depth characterization. Besides the fundamental aspects that are investigated herein, the photoacoustic properties were exemplarily examined using phantom gels to assess their tomographic imaging capabilities. Finally, the robust luminescence in the visible range arising from the push–pull character of the peripheral moieties demonstrated a notable independence from aggregation and was exemplarily implemented for optical imaging (FLIM) through time-resolved multiphoton micro(spectro)scopy.

## 1. Introduction

Over the past decade, there has been a rising interest in the design and synthesis of compounds exhibiting dual emission (DE) [1]. While most organic molecules typically manifest a single characteristic fluorescence, certain distinctive luminophores, wherein electron donor and electron acceptor moieties are connected by a single bond, may exhibit a dual emission [2]. The phenomenon of dual fluorescence (DF) was initially observed for 4-(dimethylamino)benzonitrile (DMABN) by Lippert et al. [3]. It arises from two distinct conformations of the same molecule in the first excited singlet state (S_1_), specifically, the locally excited state (LE) and the intramolecular charge-transfer state (ICT) [4]. Subsequently, several other systems were designed incorporating a strong electron-donating or -withdrawing group [5,6,7,8,9,10,11]. Owing to their nonlinear optical properties, these compounds have been extensively explored as part of the development of organic materials, serving various applications including their use as electrooptical switches, chemical sensors, and fluorescent markers [12,13,14,15]. 

Generally, due to their broad application range as functional materials for semiconductors, gas sensors, nonlinear optical limiters, liquid crystals and as sensitizers for photodynamic therapy (PDT) in cancer treatments, among many others, metal phthalocyanines (Pcs) are counted among the most extensively investigated dyes in history, besides others such as cyanine colorants and BODIPY derivatives [16,17,18]. Metal naphthalocyanines (Ncs) constitute expanded analogues of their Pc counterparts that possess a linearly annulated benzene ring in the periphery of the macrocyclic core that leads to an about 100 nm red shift of the absorption band in the red if compared with Pcs. This shift, known as the rule of “100 nm”, results from the destabilization of the HOMO (highest occupied molecular orbital) and the associated decrease in the HOMO-LUMO (lowest unoccupied molecular orbital) gap [19]. Thus, Ncs are able to act as versatile tetradentate chelators for various metals and show a strong absorption in the near-infrared region (750–900 nm) with high molar absorption coefficients. The intense absorption and fluorescence bands of naphthalocyanines in the near-infrared offers interesting opportunities such as the fabrication of high-performance NIR photodiodes and bioimaging [20,21], and the complexation of open-shell or late-transition element cations can add further functionalities related to spin–spin or spin–orbit coupling, respectively.

The structural flexibility of Ncs has been extensively illustrated through a diverse range of metal complexes and a wide array of substituents that can be attached to the periphery of the core or as axial ligands [19,22,23,24,25,26,27]. This chemical modification induces specific alterations in the electronic structure of the macrocyclic core, providing precise control over the physicochemical characteristics. However, despite their ability to absorb in the far red/near-infrared region, Ncs have been relatively neglected, mainly because of their intricate synthesis, with difficult purification, poor solubility, and a tendency to form inactive aggregates, owing to strong *van der Waals* interactions upon stacking [28,29]. As a consequence of their high tendency to form aggregates, a drop of fluorescence and singlet oxygen quantum yields is observed [30,31]. There have been several structural variations introduced to overcome the solubility and aggregation issues of (na)phthalocyanines, including the insertion of bulky substituents in the peripheral positions of the macrocycles [25,32,33,34,35], the addition of axial ligands to the central atom [28,30,36,37,38,39], or the encapsulation of the dye in colloidal particles [40,41]. Furthermore, peripheral functional groups in Ncs macrocycles may be utilized to further tailor intra- and intermolecular interactions as well as optical, photochemical, and electrochemical properties; particularly, aromatic groups increase intermolecular *π*-*π* interactions [19,34]. However, all these derivatives still exhibit a strong tendency to aggregate in aqueous media, which can significantly reduce their performance due to self-quenching [42]. In the case of Pcs, these issues were solved by the introduction of several hydrophilic and amphiphilic groups (e.g., carboxylates, sulfonates, phosphonates, PEG chains) [43,44,45,46,47,48], as well as bulky axial ligands (e.g., −OSiMe_2_(CH_2_)_3_NMe_2_ and −OCH(CH_2_NMe_2_)_2_) [49,50,51,52,53] in the periphery of the macrocycle or at the central atom, respectively. Among these strategies, positively charged macrocycles are of particular interest, as they might target highly vulnerable intracellular sites and produce effective DNA damage in the context of phototherapy [42]. Moreover, positively charged Pcs have been successfully employed for the photoinactivation of both Gram-negative and Gram-positive bacteria [54,55,56]. However, in the case of water-soluble Ncs, the majority of the reported exemplars are based on silicon(IV)-based coordination compounds, mainly due to the presence of two axial positions that can be readily modified to reduce aggregation while improving solubility in aqueous media [27,57]. While peripherally substituted water-soluble Pcs have been widely discussed in the literature [30,58], their Ncs counterparts with aqueous solubility have remained vastly unexplored [29,59]. Despite exhibiting NIR absorption and emission, Ncs exhibiting dual fluorescence (vis/NIR) have not been reported so far. 

With this background in mind, we herein report on an unprecedented design pattern for single-component yet dual-emissive zinc(II) naphthalocyaninates, including a detailed synthesis as well as a structural and photophysical characterization. In order to suppress the intermolecular interactions and to improve their solubility, we aimed at the tailored substitution on the *γ*-position of the macrocyclic ligand to introduce eight 4-*N*,*N*-dimethylaminophen-4-yl moieties, which were further quaternized with methyl iodide to enhance their hydrophilicity. Moreover, substitution with 4-*N*,*N*-dimethylaminophen-4-yl moieties opens up new possibilities for push–pull-based two-photon excitability and aggregation-independent luminescence in the visible region of the electromagnetic spectrum. To assess the charge-transfer nature of the excited state that was provided by the dimethylamino substitutents, an analogous 4-methoxyphen-4-yl-substituted zinc(II) naphthalocyaninate was synthesized. The assignment of the emissive states was carried out by TD-DFT calculations while rationalizing the electronic transitions dominating the absorption and emission spectra of these complexes. Thus, we anticipate that this strategy for preparing dual-emissive (visible and NIR) naphthalocyanines will pave the way for future innovation and accelerated developments in a broad range of application fields, including optoelectronics, energy conversion, and as contrast agents that are able to provide two orthogonal readouts for multiscale–multimodal imaging.

## 2. Results and Discussion

### 2.1. Design, Synthesis, Purification, and Structural Characterization

**Figure 1:**
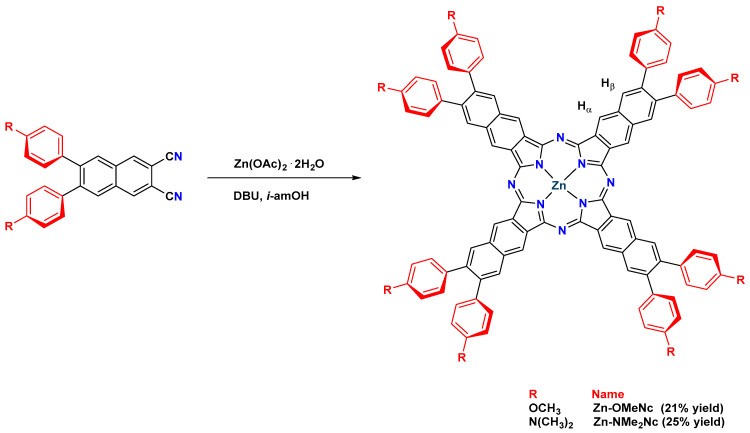
Synthesis of zinc(II) naphthalocyaninates.

The peripheral functionalization was achieved by means of a Suzuki–Miyaura coupling reaction between 6,7-dibromo-2,3-dicyanonaphthalene (**1**) and the corresponding boronic acids, following our previously reported methodology [60]. This substitution pattern was chosen in accordance with our recent report showing that the push–pull character and perpendicular-to-plane rigidified arrangement of the two phenyl moieties can provide strong luminescence in aggregates as well as in solution. The detailed synthetic procedure is shown in Section 3 (*vide infra*). The substituted naphthalonitriles **NMe_2_** and **OMe** were converted into the corresponding naphthalocyaninato zinc(II) complexes **Zn-OMeNc** and **Zn-NMe_2_Nc** by refluxing them in isoamyl alcohol (*i*-amOH) in the presence of 1,8-diazabicycloundec-7-ene (DBU) in catalytic amounts (Figure 1).

**Figure 2:**
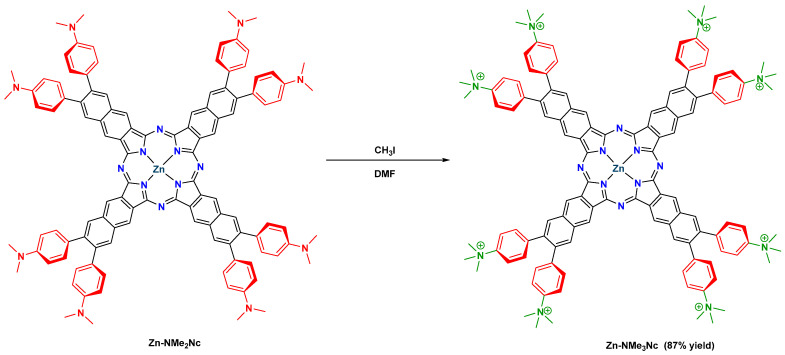
Synthesis of 3,4,12,13,21,22,30,31-octakis(4-(*N*,*N*,*N*-trimethylammonium)phenyl)-2,3-naphthalocyaninato zinc(II) (**Zn-NMe_3_Nc**).

The reaction was performed under inert conditions in order to prevent the oxidation of the macrocycle by atmospheric oxygen at high temperatures. The obtained products contained some fluorescent impurities (most likely low-molecular-weight condensates preceding the cyclotetramerization); attempts to use column chromatography with silica or alumina as stationary phases in combination with classical organic solvent gradients failed, as the polar components of the reaction mixture strongly bind to both materials. Reverse-phase silica was also explored for purification; also in this case, it was observed that the product strongly binds to the stationary phase. Thus, the crude reaction mixture was subjected to multiple Soxhlet extractions to remove bulk impurities. The obtained products were further purified by size exclusion chromatography (Sephadex LH-20, Amersham Pharmacia Biotech AB, Uppsala, Sweden) in *N*,*N*-dimethylformamide (DMF). Finally, the water-soluble octa-cationic zinc(II) naphthalocyaninate **Zn-NMe_3_Nc** was obtained by treating **Zn-NMe_2_Nc** with an excess of methyl iodide in DMF (Figure 2). Besides UV-vis absorption spectroscopy, the Ncs were structurally characterized by means of mass spectrometry and NMR spectroscopy. Due to their intrinsic aggregation tendency, attaining well-resolved ^1^H- and ^13^C-NMR spectra proved to be particularly challenging. Nonetheless, we were able to obtain precise 2D-NMR spectra (including an unambiguous assignment for all peaks).

In order to obtain a better resolution in the ^1^H-NMR spectra of **Zn-OMeNc**, **Zn-NMe_2_Nc**, and **Zn-NMe_3_Nc**, DMSO was used as the solvent in NMR spectroscopy, as it suppresses aggregation caused by stacking. Due to the de-shielding effect of the porphyrazinato core, a clear downfield shift of H_α_ protons compared to H_β_ was observed [34]. The interpretation of all signals in the ^1^H-NMR spectra of the Ncs **Zn-OMeNc**, **Zn-NMe_2_Nc**, and **Zn-NMe_3_Nc** were enabled by ^1^H-^1^H-COSY, ^1^H-^13^C-HSQC, ^1^H-^13^C-HMBC, and ^1^H-^1^H-ROESY spectroscopies, where all signals were unambiguously assigned (also for the naphthalonitriles, see SI, Figures S1–S26). From the ^1^H-NMR spectra of **Zn-OMeNc** and **Zn-NMe_2_Nc**, it was clear that these two coordination compounds aggregate at the required concentrations, whereas the eight positive charges in the periphery of **Zn-NMe_3_Nc** suppressed aggregation and yielded well-resolved ^1^H-NMR spectra (Figure S21). Hence, the proton signals from **Zn-NMe_3_Nc** were better resolved than those from **Zn-OMeNc** and **Zn-NMe_2_Nc**. In the ^1^H-^1^H-ROESY spectra, cross-peaks were observed between the signals of H_α_ and H_β_ protons and between the protons of the phenyl groups and signals corresponding to H_β_ (see SI, Figures S14, S20 and S26). From ^1^H-^1^H-COSY, additional groups of cross-peaks were found between the protons of the phenyl groups (see SI, Figures S11, S17 and S23). The mass spectra of **Zn-NMe_3_Nc** were obtained by using ESI-EM, whereas for **Zn-OMeNc** and **Zn-NMe_2_Nc**, MALDI-TOF had to be employed. The MS demonstrated a good agreement between the expected and observed peaks (see SI, Figures S27–S31). 

### 2.2. UV-Vis Absorption, Steady-State, and Time-Resolved Photoluminescence Spectroscopies

**Figure 3:**
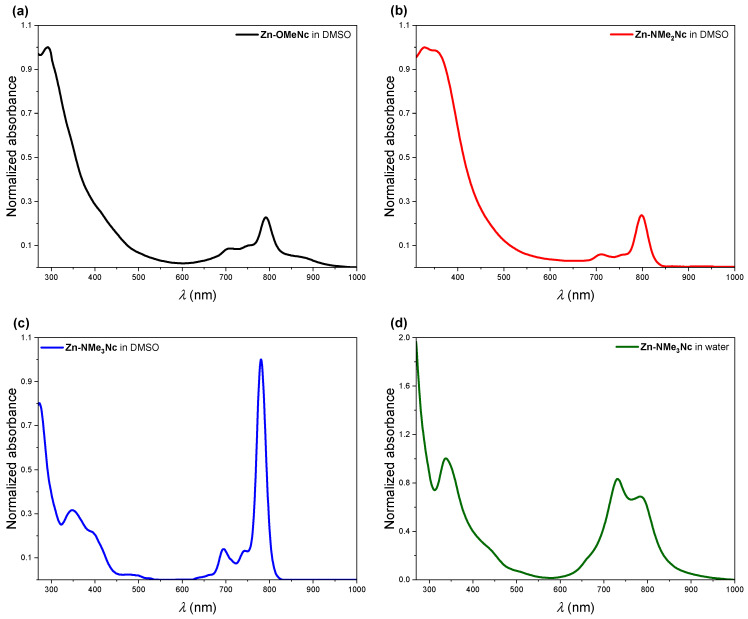
UV-vis absorption spectra normalized at the corresponding *λ*_max_ for **Zn-OMeNc** (**a**), **Zn-NMe_2_Nc** (**b**), and **Zn-NMe_3_Nc** (**c**) in DMSO, as well as for **Zn-NMe_3_Nc** in water (**d**).

In agreement with the characteristic spectroscopic features of metal (na)phthalocyaninates, the UV-vis absorption spectra of the herein reported complexes feature a *Soret*-band (or B-band) in the UV range and Q-band in the NIR region [25]. Figure 3 depicts the absorption spectra of **Zn-OMeNc**, **Zn-NMe_2_Nc**, and **Zn-NMe_3_Nc** in DMSO and in water. A red shift of the Q-band was observed when going from **Zn-OMeNc** (794 nm) to **Zn-NMe_2_Nc** (800 nm), implying that the energy gap between the HOMO and the LUMO is reduced when introducing a stronger electron *π*-donor (i.e., replacement of methoxy by dimethylamino moieties) [61,62]. Consistently, in the case of **Zn-NMe_3_Nc**, a blue shift was observed (781 nm) in DMSO, due to the reduced electron-donating capacity of the cationic trimethylammonium groups (Table 1).

As shown in Figure 3a,b, the Q-band appears to be less intense than the B-band, which is surprising when considering that the latter is usually weaker in metal (na)phthalocyaninates. This observation can be attributed to the strong absorption in the blue and in the UV related to the eight methoxy- or dimethylamino-substituted moieties, which actually exhibit a sizeable push–pull character (in fact, this blue-shifted band is drastically reduced upon quaternization, as seen by the comparison of **Zn-NMe_2_Nc** with its derivative **Zn-NMe_3_Nc**; *vide infra* regarding the additional influence of aggregation phenomena on the relative intensities in the red and in the blue region of the spectrum). Similar trends were previously reported for Zn(II) phthalocyanines [63,64]. The formation of red-shifted aggregates plays a role in the case of **Zn-OMeNc**, in agreement with the broadened signals that are observed in NMR spectroscopy (see SI, Figure S9); hence, the low molar absorption coefficient (*ε*) at *λ*_max_ = 794 nm (*ε* = 4.4 × 10^4^ M^−1^ cm^−1^) and the rather weak absorption band at around 876 nm point towards *J*-aggregation. Similarly, in the case of **Zn-NMe_2_Nc**, the intensity of the Q-band appears to be reduced if compared with the B-band, an observation that is reinforced by aggregation; therefore, a relatively low molar absorption coefficient at *λ*_max_ = 800 nm (*ε* = 5.3 × 10^4^ M^−1^ cm^−1^) is measured. Typically, the values for Q-bands in metal (na)phthalocyaninates are of the order of 10^5^ M^−1^ cm^−1^. Thus, by comparison of the three compounds presented herein, it is clear that the relatively high intensity of the B-band is prominently enhanced in the cases of **Zn-OMeNc** and **Zn-NMe_2_Nc**, due to the charge-transfer absorption of the eight aromatic electron-donating moieties (methoxy- or dimethylaminophenyl, respectively), which overlap with the B-band and disappear upon quaternization (*vide infra*); in addition, aggregation compromises the intensity of the Q-band. 

Nonetheless, the eight positive charges of **Zn-NMe_3_Nc** suppress aggregation in DMSO, and the relative intensity of the Q-band vs. the B-band fall in the expected range for metal (na)phthalocyaninates [25,65]. However, the absorption spectrum of **Zn-NMe_3_Nc** in water showed clear differences when compared to its spectrum in DMSO. In water, **Zn-NMe_3_Nc** still exhibits *H*-aggregation, as evidenced by the presence of two non-vibrational shoulders in the Q-band region (Figure 3d). The lower energy (red-shifted) band at about 784 nm can be attributed to the monomeric species, whereas the higher energy (blue-shifted) band at 732 nm suggests the presence of *H*-aggregates, as it was previously reported for comparable (na)phthalocyanines [29,30,31,66]. The formation of *H*-aggregates in water is more evident when comparing the molar absorption coefficients (*ε*) of **Zn-NMe_3_Nc** in DMSO vs. water. In DMSO, the *H*-aggregates are disrupted, and **Zn-NMe_3_Nc** shows a characteristically high *ε* value at *λ*_max_ = 781 nm (*ε* = 5.5 × 10^5^ M^−1^ cm^−1^). This contrasts with the spectrum in water, where **Zn-NMe_3_Nc** peaks at *λ*_max_ = 732 nm (*ε* = 2.2 × 10^5^ M^−1^ cm^−1^), and *λ*_max_ = 784 nm (*ε* = 1.8 × 10^5^ M^−1^ cm^−1^) (Table 1). In water, *H*-aggregation reduces the Q-band intensity with respect to the B-band, and the molar absorption coefficient in the red drops by a factor of roughly two, which points toward dimerization. Despite the clear aggregation phenomena discussed above, the Lambert–Beer law was obeyed within the experimental range for all these compounds (see SI, Figures S35–S47) [67]. In general, the molar absorption coefficients were obtained from the slope of the absorbance versus the concentration of the samples (the latter were determined by total reflection X-ray fluorescence (TXRF) addressing the concentration of Zn).

**Figure 4:**
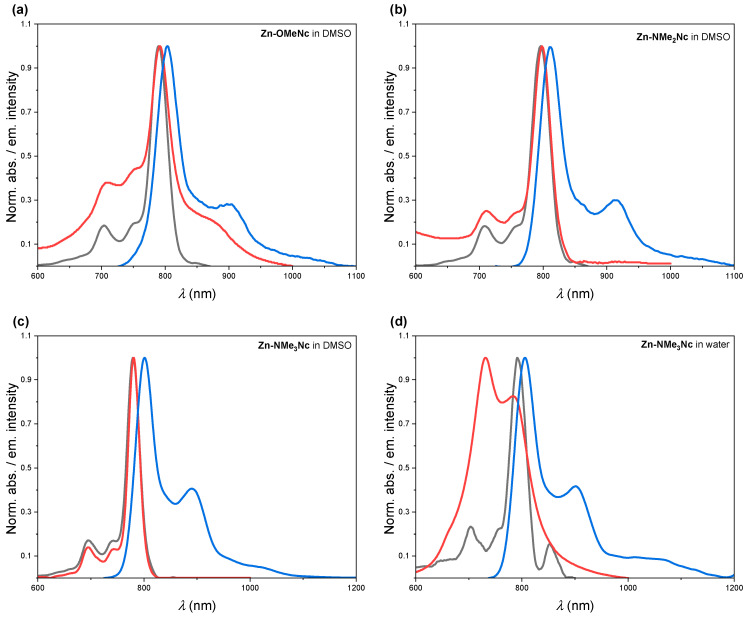
Absorption (red), excitation (black, *λ*_em_ = 920 nm), and emission (blue, *λ*_ex_ = 750 nm) spectra of **Zn-OMeNc** (**a**), **Zn-NMe_2_Nc** (**b**), **Zn-NMe_3_Nc** in DMSO (**c**) and **Zn-NMe_3_Nc** in water (**d**).

The UV-vis absorption, steady-state photoluminescence emission and excitation spectra of **Zn-OMeNc**, **Zn-NMe_2_Nc**, and **Zn-NMe_3_Nc** were measured in DMSO (Figure 4). In the case of **Zn-NMe_3_Nc** in water, a clear narrowing of the excitation spectrum was observed compared with the absorption spectrum, as only the emissive monomers were addressed, whereas all species (even aggregates) in a solution contribute to the broadened absorption. The proximity of the Q-band-related emission with respect to the Q-band excitation and absorption for **Zn-OMeNc**, **Zn-NMe_2_Nc**, and **Zn-NMe_3_Nc** in DMSO indicate that the nuclear configurations of the ground and excited states are comparable and therefore practically not affected by the excitation wavelength.

**Figure 5:**
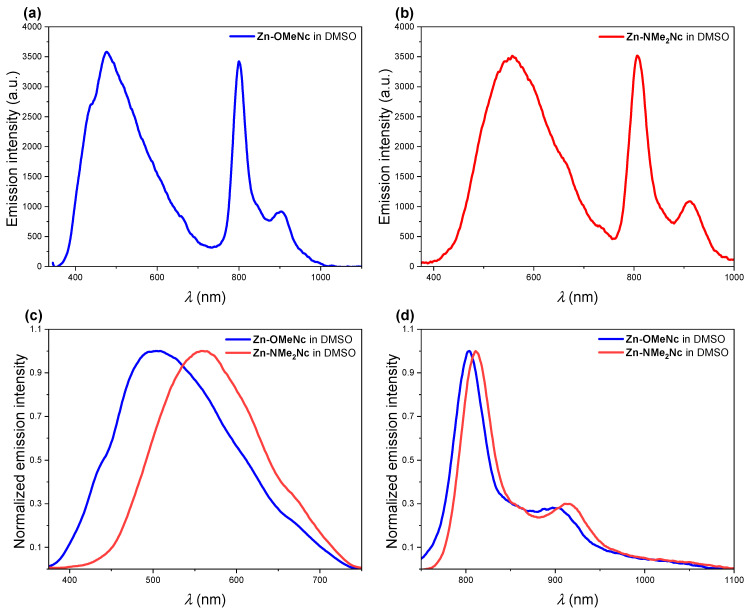
Fluorescence in DMSO: (**a**) full-range emission spectrum of **Zn-OMeNc** (blue), *λ*_ex_ = 335 nm; (**b**) full-range spectrum of **Zn-NMe_2_Nc** (red), *λ*_ex_ = 335 nm; (**c**) normalized emission spectra of **Zn-OMeNc** (blue) and **Zn-NMe_2_Nc** (red) in the visible region, *λ*_ex_ = 335 nm; (**d**) normalized emission spectra of **Zn-OMeNc** (blue) and **Zn-NMe_2_Nc** (red) in the near-infrared region, *λ*_ex_ = 750 nm. In the full-range emission spectra (**a**,**b**), the bands may appear slightly different (if compared with (**c**,**d**), both in shape and intensity), due to the different instrumental settings and filters employed (for details, see Section 3.3).

In agreement with previous reports on metal (na)phthalocyaninates, the photoluminescence emission spectra of **Zn-OMeNc**, **Zn-NMe_2_Nc**, and **Zn-NMe_3_Nc** mirror the excitation spectra with a typically small Stokes shift (Figure 4), if referring to the red-shifted Q-band. As can be observed in the full-range emission spectra (recorded while exciting in the UV), the compounds feature a surprisingly prominent dual emission, which will be discussed below (*vide infra*). This phenomenon was initially noticed while measuring the emission spectra of **Zn-NMe_2_Nc** in DMSO at different excitation wavelengths (*λ*_ex_). After varying *λ*_ex_, a broad emission band with a *λ*_em_ = 560 nm (*λ*_ex_ = 335 nm) and a sharp near-infrared emission with *λ*_em_ = 811 nm (*λ*_ex_ = 750 nm) was observed (Figure 5c,d, Table 2). In order to understand this rather unique dual emission, the analogous **Zn-OMeNc** was also designed, prepared, and studied. Interestingly, **Zn-OMeNc** also exhibited two clear emission bands, with a *λ*_em_ = 508 nm (*λ*_ex_ = 335 nm) and *λ*_em_ = 804 nm (*λ*_ex_ = 750 nm) in DMSO (Figure 5c,d, Table 2). It is important to mention that when using *λ*_ex_ = 335 nm and screening the emission from the blue to the NIR region to acquire the full-range fluorescence spectra, both bands are visible in the case of **Zn-OMeNc** and **Zn-NMe_2_Nc** (Figure 5a,b, Table 2). 

**Figure 6:**
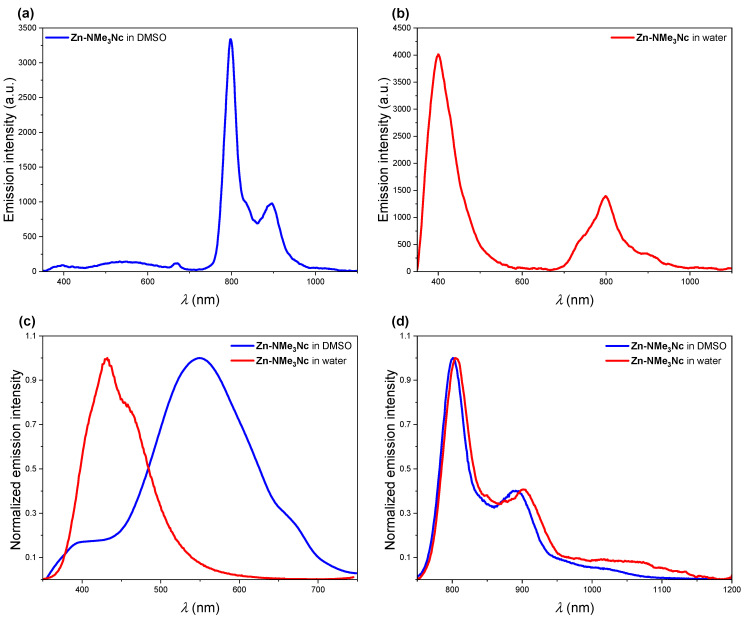
Fluorescence in DMSO and in water: (**a**) full-range emission spectrum of **Zn-NMe_3_Nc** (blue) in DMSO, *λ*_ex_ = 335 nm; (**b**) full-range emission spectrum of **Zn-NMe_3_Nc** (red) in water, *λ*_ex_ = 335 nm; (**c**) normalized emission spectra of **Zn-NMe_3_Nc** in DMSO (blue) and water (red) in the visible region, *λ*_ex_ = 335 nm; (**d**) normalized emission spectra of **Zn-NMe_3_Nc** in DMSO (blue) and water (red) in the near-infrared region, *λ*_ex_ = 750 nm. In the full-range emission spectra (**a**,**b**), the bands may appear slightly different (if compared with (**c**,**d**), both in shape and intensity) due to the different instrumental settings and filters employed (for details see Section 3.3).

A similar trend was observed for **Zn-NMe_3_Nc** when measured in water and in DMSO. In DMSO, **Zn-NMe_3_Nc** exhibited two emission bands, with a *λ*_em_ = 550 nm (*λ*_ex_ = 335 nm) and *λ*_em_ = 801 nm (*λ*_ex_ = 750 nm) (Figure 6c,d, Table 2). In contrast, the emission bands measured in water were observed at *λ*_em_ = 432 nm (*λ*_ex_ = 335 nm) and *λ*_em_ = 804 nm (*λ*_ex_ = 750 nm) (Figure 6c,d, Table 2). When a full-range emission spectrum for **Zn-NMe_3_Nc** in DMSO was measured (from blue to the NIR region using *λ*_ex_ = 335 nm), a relatively faint emission band with *λ*_em_ = 550 nm and a high-intensity luminescence with *λ*_em_ = 800 nm were observed (Figure 6a). This can be attributed to the reduction in the push–pull character upon quaternization of the peripheral 4-*N*,*N*-dimethylaminophen-4-yl groups and the monomeric nature of **Zn-NMe_3_Nc** in DMSO, yielding a comparably strong NIR fluorescence and a weak visible emission. Thus, it is inferred that the monomers of **Zn-NMe_3_Nc** in DMSO yield an intense NIR emission and a comparatively weak luminescence between 400 nm and 500 nm. In contrast, the full-range emission spectrum of **Zn-NMe_3_Nc** in water (from blue to the NIR region using *λ*_ex_ = 335 nm) exhibited a substantially stronger emission band, peaking at *λ*_em_ = 400 nm, when compared with the NIR fluorescence band at *λ*_em_ = 801 nm (Figure 6b); this can be understood when taking into account that in water, *H*-aggregation suppresses the fluorescence in the NIR, and a comparatively strong residual emission remains in the visible range. The dual emission of **Zn-OMeNc**, **Zn-NMe_2_Nc**, and **Zn-NMe_3_Nc** was interpreted and assigned by TD-DFT calculations (*vide infra*).

The dual fluorescence can be explained by two sets of singlet states (with orthogonal charge-transfer vs. main-ligand-centered character). The near infrared fluorescence originates from the S_1/2_ → S_0_ transition, and the visible emission stems from the S_3_ → S_0_ transition. The doubly degenerate S_1/2_ state has a *π*–π^*^ character, whereas the S_3_ states have a charge-transfer (*n*–π^*^) character involving the electron-rich methoxy- or dimethylamino-substituted phenyl moieties. The energy gap between S_1/2_ and S_3_ states is relatively large; the lack of excited state geometry distortion (i.e., parallel potential hypersurfaces or “nested states”) hamper the non-adiabatic crossover, leading to relatively slow internal conversion processes. Hence, in the case of **Zn-OMeNc**, **Zn-NMe_2_Nc**, and **Zn-NMe_3_Nc**, both an emission in the visible region and an NIR fluorescence are observed. In the case of **Zn-NMe_3_Nc** in water, the S_1/2_ state is quenched due to aggregation, the intensity of the NIR emission is low, and the push–pull character is reduced due to quaternization.

Taking into account that the relative intensities of the two emission bands remain invariant when comparing independently synthesized batches, and considering the thorough purification that was carried out with no traces of impurities in the NMR spectra, we can confidently exclude spurious emission from side products or other contamination (photodecomposition is ruled out, as the absorption and emission spectra do not significantly vary during the photophysical characterization). Moreover, TD-DFT calculations support the assignment (*vide infra*).

### 2.3. TD-DFT Calculations

**Figure 7:**
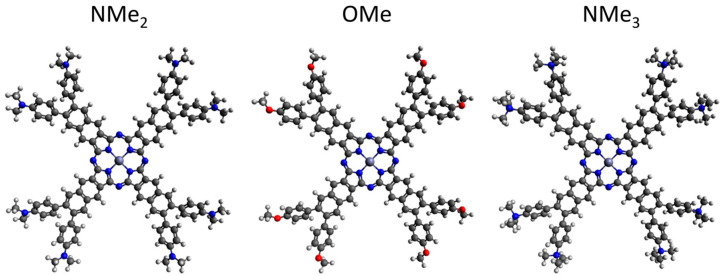
Optimized ground state structures of **Zn-OMeNc**, **Zn-NMe_2_Nc**, and **Zn-NMe_3_Nc**, obtained with DFT.

In order to assign the transitions that were observed in the absorption and fluorescence spectra, we optimized the structures of **Zn-OMeNc**, **Zn-NMe_2_Nc**, and **Zn-NMe_3_Nc** in their electronic ground states (for computational details, see Section 3.4). All three complexes exhibited a highly planar core, which was marginally influenced by the peripheral substituents (see Figure 7). At the optimized ground state geometries, the UV-vis absorption spectra were calculated using TD-DFT/B3LYP.

**Figure 8:**
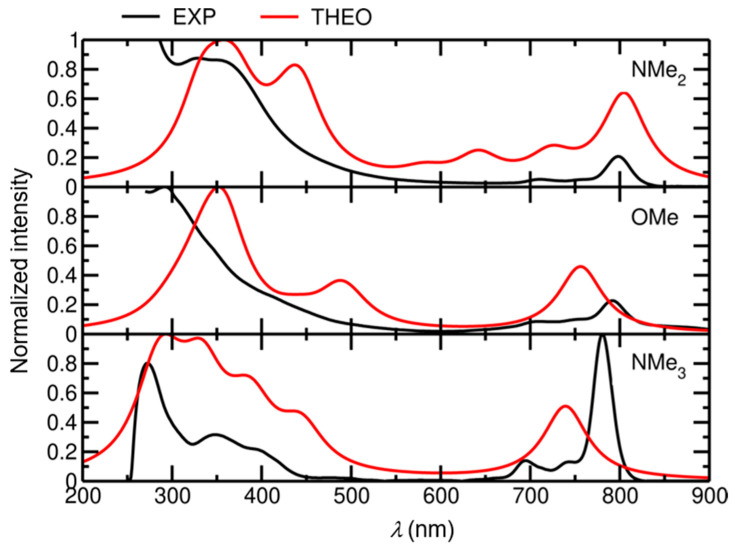
Calculated UV-vis absorption spectra of **Zn-OMeNc**, **Zn-NMe_2_Nc**, and **Zn-NMe_3_Nc** in DMSO (red lines), together with the corresponding experimental data (black lines).

**Figure 9:**
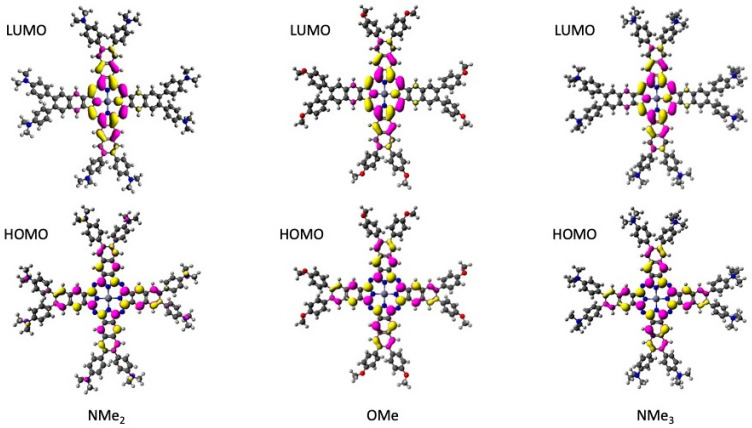
Isosurface plots (isovalue = 0.02) of the molecular orbitals of **Zn-OMeNc**, **Zn-NMe_2_Nc**, and **Zn-NMe_3_Nc**, with the highest contributions describing the S_1/2_ → S_0_ de-excitation.

As can be seen from Figure 8, the theoretical spectra generally agree well with the experimental data. Due to the degeneracy of the LUMO and LUMO + 1 orbitals, the S_1_ and S_2_ states are also doubly degenerate, as they can be described by HOMO → LUMO and HOMO → LUMO+1 excitations, respectively. The band corresponding to the S_0_ → S_1/2_ transition—located at 805 nm, 756 nm, and 740 nm for **Zn-NMe_2_Nc**, **Zn-OMeNc**, and **Zn-NMe_3_Nc**, respectively—shows a progressive blue shift with decreasing electron donor ability, in good agreement with the experimental data (and also qualitatively with respect to the relative intensities of the B- and Q-bands). The high intensity of this NIR maximum can be explained by the fact that it represents a local excitation within the 2,3-naphthalocyaninato ligand (the black part of the structure depicted in Figure 1, see Figure 9 for a visualization of the relevant frontier orbitals).

**Figure 10:**
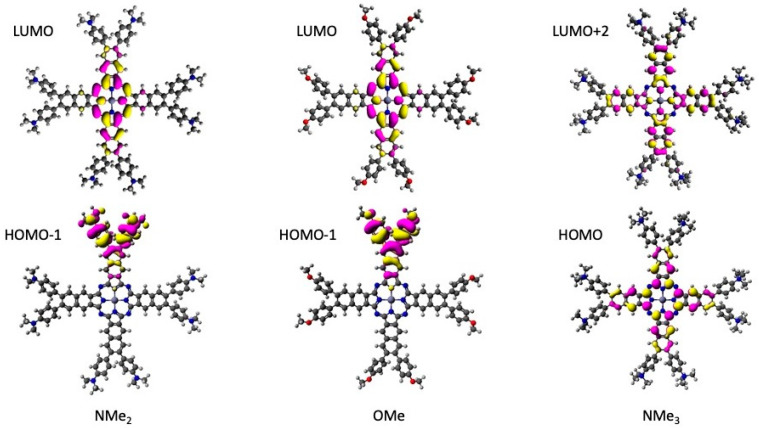
Isosurface plots (isovalue = 0.02) of the molecular orbitals of **Zn-OMeNc**, **Zn-NMe_2_Nc**, and **Zn-NMe_3_Nc**, with the highest contributions describing the S_3_ → S_0_ de-excitation.

**Figure 11:**
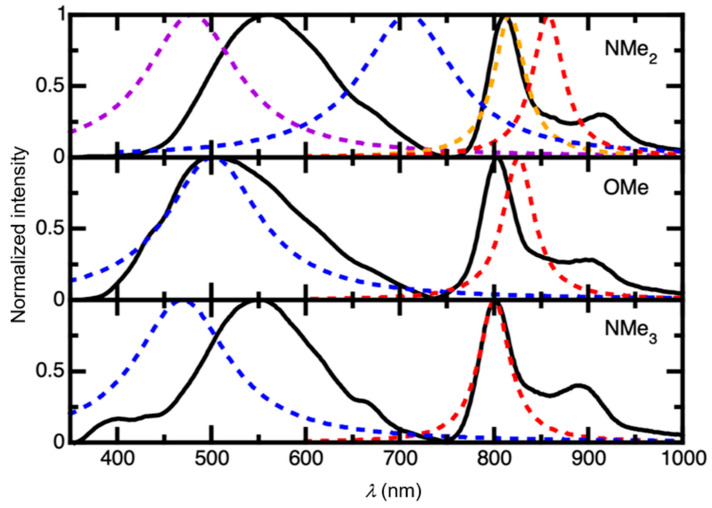
Calculated 0-0 emission wavelengths (dashed lines) from the S_1/2_ (red) and S_3_ (blue) states, obtained with the PBE0 functional, compared to experimental fluorescence spectra (black lines) of **Zn-OMeNc**, **Zn-NMe_2_Nc**, and **Zn-NMe_3_Nc** in DMSO. For **Zn-NMe_2_Nc**, additional results using the M06-2X functional are shown (S_1/2_ in orange, S_3_ in purple).

In addition, we calculated the 0-0 fluorescence wavelengths from the S_1/2_ and S_3_ states by optimizing the respective excited state geometries and subtracting the optimized ground state energy. While the S_1/2_ → S_0_ de-excitation involved a local LUMO/LUMO + 1 → HOMO configuration change (Figure 9), which was reasonably well described by all functionals that were tested herein, the S_3_ → S_0_ de-excitation turned out to be much more problematic, as it involved a pronounced charge-transfer character for the relevant excited state—at least for **Zn-OMeNc** and **Zn-NMe_2_Nc** (see Figure 10). For **Zn-NMe_2_Nc**, geometry optimization with the B3LYP functional leads to a collapse of the S_3_ state onto the S_1/2_ state, yielding spurious emission wavelengths. The long-range corrected functional CAM-B3LYP, on the other hand, significantly overestimated the S_3_ energy (see SI, Table S1). In fact, the PBE0 functional emerged as the best compromise for all three compounds, considering both emission lines in the visible and the NIR (see Figure 11). It is able to reproduce the position of the experimental 0-0 peaks accurately, including the slight blue shift when going from **Zn-NMe_2_Nc** to **Zn-NMe_3_Nc** via **Zn-OMeNc**. For **Zn-OMeNc**, the S_3_ peak was also described very well, while for **Zn-NMe_2_Nc**, the agreement with the experiment was particularly poor, with the PBE0 peak being red-shifted by about 150 nm.

Presumably, this is due to the stronger charge-transfer character in the case of **Zn-NMe_2_Nc**, with a notable amount of charge density being located at the NMe_2_ substituent in the HOMO-1. In addition, the S_3_ state contains a 7% contribution of the local HOMO → LUMO + 1 excitation. The use of the M06-2X functional leads to a considerable improvement for **Zn-NMe_2_Nc** (see Figure 11), but not for the other two compounds. PBE0 predicts the S_3_ state to be governed by a HOMO-1 → LUMO excitation in the case of **Zn-NMe_2_Nc** and **Zn-OMeNc**, whereas for **Zn-NMe_3_Nc**, the S_3_ state is dominated by a HOMO → LUMO + 2 excitation (Figure 10).

### 2.4. MSOT Imaging in Gel Phantoms 

**Figure 12:**
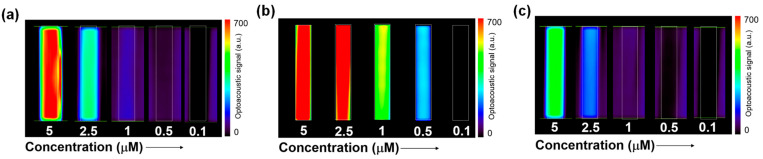
MSOT photoacoustic images of gel phantoms at different concentrations: (**a**) **Zn-Nme_2_Nc** in DMSO, (**b**) **Zn-Nme_3_Nc** in DMSO, and (**c**) **Zn-NMe_3_Nc** in water (*λ*_ex_ = 745 nm).

In recent studies, it has been demonstrated that naphthalocyaninato complexes (Ncs) hold significant potential as photoacoustic contrast agents [27,68,69]. In comparison with other organic compounds, such as cyanine dyes and nanoparticles, naphthalocyanines exhibit higher molar absorption coefficients in the NIR region, featuring sharp maxima between 770 nm and 850 nm [16]. The well-defined NIR absorption peaks mentioned above contribute to more precise spectral unmixing, distinguishing them from other exogenous contrast agents [70]. As a result, **Zn-NMe_2_Nc** and **Zn-NMe_3_Nc** were employed as representative examples for generating photoacoustic (PA) images utilizing multi-spectral optoacoustic tomography (MSOT). These measurements were conducted within tissue-mimicking gel phantoms, as depicted in Figure 12 [69]. The experiments were carried out at different concentrations, and the obtained photoacoustic profiles qualitatively agree with the absorption spectra regarding the wavelengths that induce the highest photoacoustic response. The phantom studies were performed by dissolving **Zn-NMe_2_Nc** in DMSO, **Zn-NMe_3_Nc** in DMSO, and **Zn-NMe_3_Nc** in water, respectively. 

**Figure 13:**
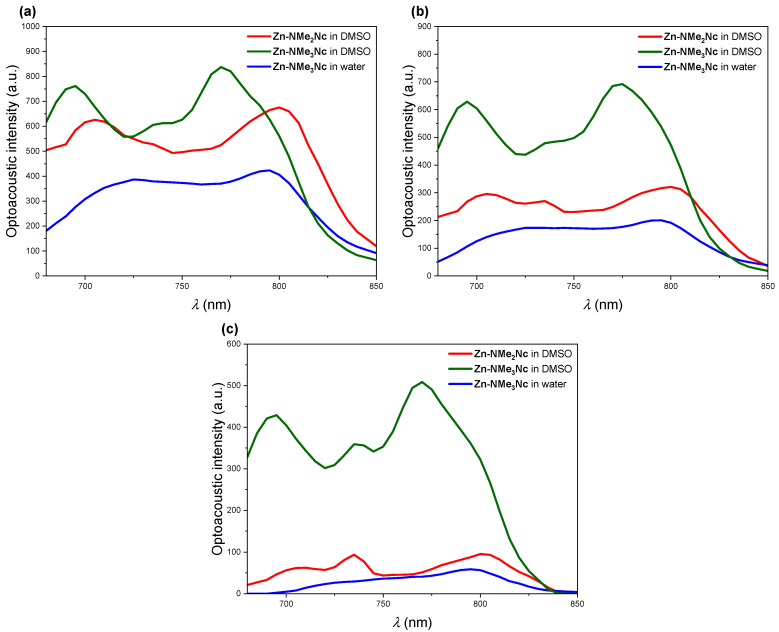
Photoacoustic spectra of **Zn-NMe_2_Nc** in DMSO (red) and **Zn-NMe_3_Nc** in DMSO (green) and water (blue) at different concentrations, obtained from reconstructed MSOT photoacoustic images. Spectra at concentration of 5 μM (**a**), 2.5 μM (**b**), and 1 μM (**c**).

Among these, **Zn-NMe_3_Nc** in DMSO provided the highest photoacoustic signal even at a low concentration (1 μM), with **Zn-NMe_2_Nc** in DMSO and **Zn-NMe_3_Nc** in water showing similar yet slightly lower PA signals (Figure 13). The observed difference in signal intensity of **Zn-NMe_3_Nc** in DMSO and water can be attributed to the monomerization in DMSO and aggregation in water. Also, when comparing the photoacoustic (Figure 13) and absorption spectra (Figure 3c) of **Zn-NMe_3_Nc** in DMSO at *λ*_max_ = 781 nm, the relative intensities of the vibronic features appear distorted. For the photoacoustic spectra of **Zn-NMe_3_Nc** in DMSO, the inner filter effect limits the excitation at the main maxima, even at very low concentrations (1 μM), while causing a saturation plateau between *λ*_max_ = 781 nm and *λ*_max_ = 700 nm. As **Zn-NMe_3_Nc** in DMSO provides the best photoacoustic signals at a low concentration, it is clear that the suppression of aggregation enhances the photoacoustic output. In the future, this could be further improved with vanadyl- or silicon-naphthalocyanines, where the axial ligands could prevent aggregation [30,69,71]; the insertion of vanadyl centers could further shift the absorption maxima to longer wavelengths in the infrared region [72,73,74].

### 2.5. Time-Resolved Multiphoton Micro(spectro)scopy 

**Figure 14:**
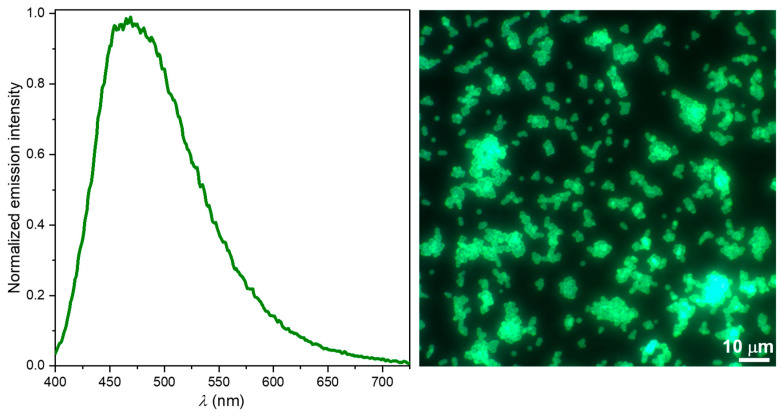
Left: emission spectrum of the **Zn-NMe_2_Nc@PSMP**, suspended in H_2_O (*λ*_ex_ = 335 nm). Right: luminescence image of the **Zn-NMe_2_Nc@PSMP**, observed under an optical microscope (scale bar = 10 μm; *λ*_ex_ = 355 ± 20 nm).

In order to demonstrate the ability to image structural features by time-resolved multiphoton micro(spectro)scopy, we employed **Zn-NMe_2_Nc** to stain 1 μm sized aminated polystyrene microparticles (PSMPs) by a swelling–diffusion processes [75], as these are well-established models for microstructured samples (Figure 14b). As depicted in Figure 14, the photoluminescence emission spectra of **Zn-NMe_2_Nc**-loaded PSMPs (**Zn-NMe_2_Nc@PSMP**) in water show a broad emission band in the green (*λ*_max_ ≈ 470 nm), but no emission was observed in the near-infrared region (Figure 14a); this points towards aggregation-caused quenching of the fluorescence from the macrocycle, as it was previously reported for other (na)phthalocyanines [76]. However, the distinct emission in the visible region, originating from the push–pull character of the excited side groups, can be utilized as an additional optical readout, despite the lack of intrinsic luminescence from the macrocycle. In fact, the push–pull side groups have been previously introduced in our recent work as robust luminophores, both in aggregated and in monomeric forms [60]. The excited state lifetime of the stained microparticles in liquid water was determined, and a *τ*_av_amp_ = 5.1 ns was obtained (Figure S56).

**Figure 15:**
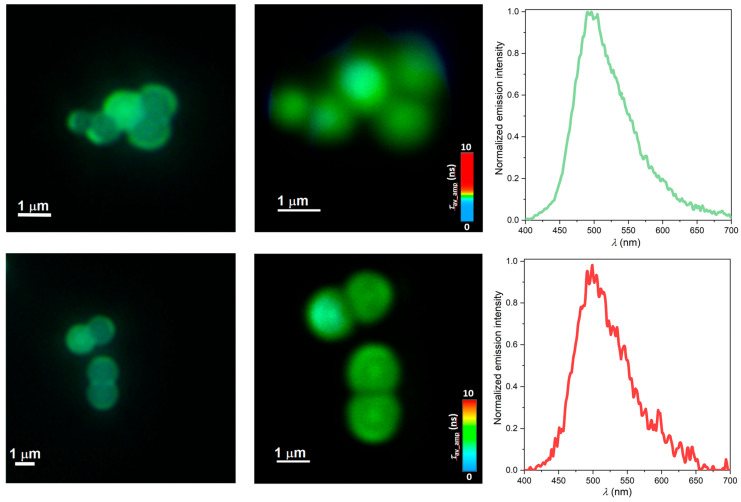
Upper row: Fluorescence micrograph of **Zn-NMe_2_Nc@PSMP** (**left**); fluorescence lifetime map measured with single-photon excitation (SPE, *λ*_ex_ = 375 nm, **center**); emission spectrum of a discrete particle using SPE (*λ*_ex_ = 375 nm, **right**). Lower row: Fluorescence micrograph of **Zn-NMe_2_Nc@PSMP** (**left**); fluorescence lifetime map measured with two-photon excitation (TPE, *λ*_ex_ = 810 nm, **center**); emission spectrum of a discrete particle using TPE (*λ*_ex_ = 810 nm, **right**). Raw photoluminescence decays and further details can be found in Figures S57 and S58.

Further experiments were carried out to assess the photophysical properties of discrete **Zn-NMe_2_Nc@PSMP**. Briefly, **Zn-NMe_2_Nc@PSMP** were placed on a microscope slide and analyzed using single-photon excitation (SPE) to carry out fluorescence lifetime imaging microscopy (FLIM). As depicted in Figure 15, the particles were homogeneously stained with the push–pull luminophore, confirming the observation that is shown in Figure 14, while possessing a homogeneously distributed amplitude-weighted average lifetime of *τ* ≈ 3.9 ns (Figure 15 and Figure S57). In addition, employing a spectrophotometer coupled by an optical fiber to the confocal microscope, the emission spectra of discrete **Zn-NMe_2_Nc@PSMP** entities were obtained as dry samples while consistently reproducing the results for **Zn-NMe_2_Nc@PSMP** that was suspended in liquid water (Figure 15). In contrast to single-photon excitation (SPE), two-photon excitation (TPE) laser scanning microscopy is often anticipated to increase cell survival and tissue penetration [77]. Thus, we explored herein a way to increase the detection sensitivity due to an improved sample penetration employing a near-infrared TPE laser; we therefore performed comparable FLIM experiments using a high-intensity Ti:Sa oscillator as the two-photon excitation source (Figure 15), where comparable photoluminescence lifetime maps were obtained. In addition, the emission spectra of discrete **Zn-NMe_2_Nc@PSMP** were also measured by TPE. These experiments demonstrate that no significant differences are to be expected when using SPE and TPE (both in lifetimes (Figures S57 and S58) and in the emission spectra) for optical imaging, while providing a second readout for microscopic imaging.

## 3. Materials and Methods

All chemicals were purchased with the maximum quality available from Sigma Aldrich (Taufkirchen, Germany) or from TCI Chemicals (Eschborn, Germany) and used without further purification. Reactions were carried out using dried solvents (99.9% purity) under argon atmosphere. They were monitored by thin-layer chromatography (TLC), which was performed on 0.2 mm Macherey-Nagel ALUGRAM (Eupen, Belgium) precoated silica gel aluminum sheets. Spots were visualized by a UV handlamp (254 and 365 nm). Silica gel 60 (0.063–0.200 mm, Merck, Darmstadt, Germany) was used for column chromatography, if not otherwise stated. Fresh spectroscopic-grade solvents (Uvasol^®^, Merck, Darmstadt, Germany) were utilized for the spectroscopic studies.

### 3.1. Synthesis and Characterization of 6,7-Disubstituted Naphthalene-2,3-dicarbonitriles and Zinc(II) Naphthalocyanines

#### 3.1.1. General Procedure for the Synthesis of 6,7-Disubstituted Naphthalene-2,3-dicarbonitriles

**Scheme 1:**
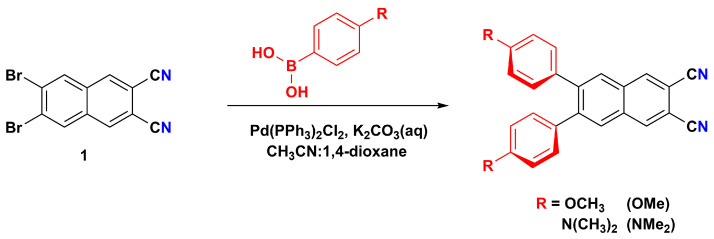
Synthesis of 6,7-disubstituted naphthalene-2,3-dicarbonitriles.

6,7-dibromonaphthalene-2,3-dicarbonitrile (**1**) was synthesized according to a published procedure [23,78,79]. The 6,7-disubstituted naphthalene-2,3-dicarbonitriles were obtained by Suzuki–Miyaura reactions from **1** with the corresponding boronic acids (Scheme 1) [60]. Briefly, a mixture of **1** (1 equiv.), the corresponding *p*-substituted-phenylboronic acid (4 equiv.), and a saturated aqueous solution of K_2_CO_3_ (5 mL) were stirred in 50 mL of a boiling mixture of 1,4-dioxane:acetonitrile (8:3 *v*/*v*) under argon. Afterwards, palladium(II)bis(triphenylphosphane) dichloride (0.01 equiv.) was added to the boiling mixture. The reaction was stirred for 6 h (TLC control: Al_2_O_3_, F_254_, ethyl acetate:hexane, 1:4). After consumption of the starting materials, the reaction mixture was cooled down to room temperature and water was added. The product was collected by extraction with dichloromethane, and the organic layer was dried with anhydrous MgSO_4_. The residue was treated by flash chromatography on silica (ethyl acetate:hexane, 1:2) to remove impurities. The resulting compound was additionally purified by column chromatography on silica using DCM as the eluent to obtain the corresponding *p*-phenyl-6,7-disubstituted naphthalene-2,3-dicarbonitrile.

#### 3.1.2. Synthesis of 6,7-Bis(4-methoxyphenyl)naphthalene-2,3-dicarbonitrile (**OMe**)

**OMe** was synthesized according to the general procedure using **1** (50 mg, 0.15 mmol), 4-methoxyphenylboronic acid (91 mg, 0.6 mmol), and a catalytic amount of palladium(II)bis(triphenylphosphane) dichloride (1 mg, 0.0015 mmol). Molecular formula: C_26_H_18_N_2_O_2_ (pale yellow solid). Yield: 51% (29 mg, 0.07 mmol).

^1^H-NMR (500 MHz, DCM-*d*_2_): *δ* (ppm) = 8.37 (s, 2H, 3-*H*), 7.96 (s, 2H, 5-*H*), 7.18–7.12 (m, 4H, 8-*H*), 6.86–6.82 (m, 4H, 9-*H*), 3.80 (s, 6H, 12-*H*).

^13^C-{^1^H}-NMR (126 MHz, DCM-*d*_2_): *δ* (ppm) = 159.76 (C-10), 144.47 (C-6), 136.01 (C-3), 132.72 (C-4), 132.54 (C-7), 131.34 (C-8), 130.13 (C-5), 116.69 (C-1), 114.09 (C-9), 110.16 (C-2), 55.65 (C-12).

MS-ESI-EM (CH_2_Cl_2_, M = C_26_H_18_N_2_O_2_): *m*/*z* calc. for [M + H]^+^ = 390.13628; found 390.13624 for [M + H]^+^.

#### 3.1.3. Synthesis of 6,7-Bis(4-(dimethylamino)phenyl)naphthalene-2,3-dicarbonitrile (**NMe_2_**)

**NMe_2_** was synthesized according to the general procedure using **1** (50 mg, 0.15 mmol), 4-methoxyphenylboronic acid (98 mg, 0.6 mmol), and a catalytic amount of palladium(II)bis(triphenylphosphane) dichloride (1 mg, 0.0015 mmol). Molecular formula: C_28_H_24_N_4_ (yellow solid). Yield: 63% (39 mg, 0.09 mmol).

^1^H-NMR (400 MHz, DCM-*d*_2_): *δ* (ppm) = 8.30 (s, 2H, 3-*H*), 7.88 (s, 2H, 5-*H*), 7.15–7.09 (m, 4H, 8-*H*), 6.69–6.61 (m, 4H, 9-*H*), 2.96 (s, 12H, 12-*H*).

^13^C-{^1^H}-NMR (101 MHz, DCM-*d*_2_): *δ* (ppm) = 150.36 (C-10), 145.08 (C-6), 135.81 (C-3), 132.55 (C-4), 130.88 (C-8), 129.70 (C-5), 128.09 (C-7), 116.90 (C-1), 112.19 (C-9), 109.49 (C-2), 40.49 (C-12).

MS-ESI-EM (CH_2_Cl_2_, M = C_28_H_24_N_4_): *m*/*z* calcd. for [M + H]^+^ = 417.20737; found 417.20710 for [M + H]^+^.

#### 3.1.4. General Procedure for the Synthesis of Zinc(II) Naphthalocyanines

A mixture of the corresponding 6,7-disubstituted naphthalene-2,3-dicarbonitrile (1 equiv.) and Zn(OAc)_2_·2H_2_O (0.5 equiv.) was refluxed in 10 mL *i*-amOH (isoamyl alcohol) for 6 h with catalytic amounts of DBU (Figure 1). The reaction mixture was cooled to room temperature, and a MeOH:H_2_O mixture (20:1 *v*/*v*) was added. The precipitate was filtered and washed with a MeOH:H_2_O (10:1 *v*/*v*) mixture. However, the obtained precipitate contained some impurities, which were removed by a Soxhlet extraction with successive solvents of increasing polarity (from diethyl ether to hexane and ethyl acetate). The product was further purified by size exclusion chromatography using a resin (Sephadex-LH20, Amersham Pharmacia Biotech AB, Uppsala, Sweden) in *N*,*N*-dimethylformamide to obtain the corresponding zinc(II) naphthalocyanines.

#### 3.1.5. Synthesis of 3,4,12,13,21,22,30,31-Octakis(4-methoxyphenyl) Naphthalocyaninato Zinc(II) (**Zn-OMeNc**)

**Zn-OMeNc** was prepared by treating **OMe** (0.1 g, 0.25 mmol) with Zn(OAc)_2_·2H_2_O (0.028 g, 0.127 mmol) and using DBU as the catalyst in 10 mL *i*-amOH. The obtained product was precipitated with a MeOH:H_2_O (10:1 *v*/*v*) mixture and filtered. The purification of the obtained products involved a sequential two-step approach. Initially, a Soxhlet extraction was employed using solvents of increasing polarity (from diethyl ether to hexane and ethyl acetate). Subsequently, the resulting product underwent a final purification using size exclusion chromatography with a resin (Sephadex-LH20) in *N*,*N*-dimethylformamide. The collected fractions were concentrated to obtain the **Zn-OMeNc**. Molecular formula: C_104_H_72_N_8_O_8_Zn (brown solid). Yield: 21% (90 mg, 0.05 mmol).

^1^H-NMR (400 MHz, DMSO-*d*_6_): *δ* (ppm) = 8.50–8.26 (br, m, 8H, 3-*H*), 8.24–7.97 (br, m, 8H, 5-*H*), 7.15 (m, 16H, 8-*H*), 6.90 (m, 16H, 9-*H*), 3.76 (s, 24H, 11-*H*).

^13^C-{^1^H}-NMR (125 MHz, DMSO-*d*_6_): *δ* (ppm) = 168.80 (C-1), 158.40 (C-10), 141.00 (C-6), 134.10 (C-4), 132.50 (C-7), 131.25 (C-5), 130.70 (C-8), 128.70 (C-2), 123.90 (C-3), 113.54 (C-9), 55.00 (C-11).

MALDI-TOF-MS (CHCl_3_, M = C_104_H_72_N_8_O_8_Zn): *m*/*z* calcd. for [M]^+^ = 1624.48; found 1777.51 for [M + 2 Ph]^+^, 1624.40 for [M]^+^, and 1400.36 for [M-2 OMePh]^+^.

#### 3.1.6. Synthesis of 3,4,12,13,21,22,30,31-Octakis(4-(*N*,*N*-dimethylamino)phenyl) Naphthalocyaninato Zinc(II) (**Zn-NMe_2_Nc**)

**Zn-NMe_2_Nc** was prepared by treating **NMe_2_** (0.100 g, 0.24 mmol) with Zn(OAc)_2_·2H_2_O (0.026 g, 0.120 mmol) and using DBU as the catalyst in 10 mL *i*-amOH. The obtained product was precipitated with a MeOH:H_2_O (10:1 *v*/*v*) mixture and filtered. The purification of the obtained products involved a sequential two-step approach. Initially, a Soxhlet extraction was performed using solvents of increasing polarity (from diethyl ether to hexane and ethyl acetate). Subsequently, the resulting product underwent final purification using size exclusion chromatography with a resin (Sephadex-LH20) in *N*,*N*-dimethylformamide. The collected fractions were concentrated to obtain the complex **Zn-NMe_2_Nc**. Molecular formula: C_112_H_96_N_16_Zn (brown solid). Yield: 25% (102.50 mg, 0.06 mmol).

^1^H-NMR (400 MHz, DMSO-*d*_6_): *δ* (ppm) = 8.43–8.22 (m, 8H, 3-*H*), 8.14–7.89 (m, 8H, 5-*H*), 7.08 (m, 16H, 8-*H*), 6.65 (m, 16H, 9-*H*), 2.91 (s, 48H, 11-*H*).

^13^C-{^1^H}-NMR (125 MHz, DMSO-*d*_6_): *δ* (ppm) = 168.80 (C-1), 149.10 (C-10), 141.70 (C-6), 133.9 (C-4), 130.90 (C-5), 130.10 (C-8), 128.00 (C-7), 128.0 (C-2), 123.80 (C-3), 111.70 (C-9), 39.90 (C-11).

MALDI-TOF-MS (CHCl_3_, M = C_112_H_96_N_16_Zn): *m*/*z* calcd. for [M]^+^ = 1728.73; found 1766.79 for [M + 2H_2_O]^+^, 1728.80 for [M]^+^, 1646.71 for [M-NMe_2_Ph + 2H_2_O]^+^, and 1610.74 for [M-NMe_2_Ph]^+^.

#### 3.1.7. Synthesis of 3,4,12,13,21,22,30,31-Octakis(4-(*N*,*N*,*N*-trimethylammonium)phenyl) naphthalocyaninato Zinc(II) Octaiodide (**Zn-NMe_3_Nc**)

**Zn-NMe_3_Nc** was prepared by treating **Zn-NMe_2_Nc** (0.030 g, 0.017 mmol) with methyl iodide (excess) in *N*,*N*-dimethylformamide for 3 days to quarternize the amino groups (Figure 2). Once the reaction was completed, the desired product was precipitated using acetone and then dried. The compound was further purified with a size exclusion resin (Sephadex-G25, Amersham Pharmacia Biotech AB, Uppsala, Sweden) using water as the eluent. The collected fractions were subjected to lyophilization to obtain **Zn-NMe_3_Nc**. Molecular formula: [C_120_H_120_N_16_Zn]I_8_ (greenish brown solid). Yield: 87% (28 mg, 0.015 mmol).

^1^H-NMR (400 MHz, DMSO-*d*_6_): *δ* (ppm) = 10.08 (s, 8H, 3-*H*), 8.87 (s, 8H, 5-*H*), 8.04 (d, J = 8.6 Hz, 16H, 9-*H*), 7.70 (d, J = 8.4 Hz, 16H, 8*-H*), 3.70 (s, 72H, 11-*H*).

^13^C-{^1^H}-NMR (100 MHz, DMSO-*d*_6_): *δ* (ppm) = 153.48 (C-1), 146.16 (C-10), 142.19 (C-7), 137.35 (C-6), 136.11 (C-2), 132.67 (C-4), 131.59 (C-5), 131.33 (C-8), 121.97 (C-3), 120.31 (C-9), 56.56 (C-11).

MS-ESI-EM (H_2_O, M = C_120_H_120_N_16_Zn^8+^): *m*/*z* calcd. for [M]^8+^ = 231.24; found 231.23987 for [M]^8+^. 

#### 3.1.8. **ZnNMe_2_Nc** Loaded onto Polystyrene Microparticles (PSMPs)

1 μm sized aminated polystyrene microparticles (PSMPs) were bought from Micromod GmbH (Rostock, Germany). The highly hydrophobic complex was encapsulated in the 1 μm sized aminated polystyrene microparticles (PSMPs) via a simple one-step staining procedure as follows: First, 50 mg/mL of PS microparticles were diluted in deionized water to 2.5 mg/mL (1.2 mL). A 0.8 mM solution of the **ZnNMe_2_Nc** complex in tetrahydrofuran (THF) was prepared. Then, 200 μL of this solution in THF was added to the PSMP dispersion in water, and the sample was subsequently shaken for 40 min. Shrinkage of the particles was induced by adding 200 μL of deionized water to the dispersion, thereby encapsulating the complex in the microparticles. The particles were then centrifuged at 16.000 rpm for 5 min (Megafuge™ 16 from Thermo Scientific, Schwerte, Germany). The precipitate was washed three times with ethanol/water mixtures (volume ratios of 40/60, 30/70, and 20/80) and once with deionized water to remove the excess of dye that was adsorbed onto the particles’ surface. Finally, the suspension containing the PSMPs loaded with the **ZnNMe_2_Nc** complex was diluted to 5 mg/mL (600 μL).

### 3.2. NMR Spectroscopy and Mass Spectrometry

NMR spectra were obtained at the Institut für Anorganische und Analytische Chemie (Universität Münster), using Bruker Avance Neo 500 and Bruker Avance III 400. All measurements were performed at room temperature unless otherwise specified. The ^1^H-NMR and ^13^C-NMR chemical shifts (*δ*) of the signals are given in parts per million and are referenced to the residual proton signal in the deuterated solvent DCM-*d*_2_ (^1^H: 5.32 ppm/^13^C: 54.0 ppm) or DMSO-*d*_6_ (^1^H: 2.50 ppm/^13^C: 39.52 ppm). The signal multiplicities are abbreviated as follows: s, singlet; d, doublet; t, triplet; q, quartet; br, broad; m, multiplet. 

Exact mass (EM) determination by mass spectrometry (MS) was carried out at the Organisch-Chemisches Institut (Universität Münster) using a LTQ Orbitap LTQ XL (Thermo-Fisher Scientific, Bremen, Germany) with electrospray ionization (ESI). MALDI-TOF mass spectra were taken on Autoflex Speed MALDI-TOF spectrometer with 2-[(2E)-3-(4-*tert*-butylphenyl)-2-methylprop-2-enylidene]malonitrile (DCTB) as the matrix.

### 3.3. Absorption and Fluorescence Spectroscopy

Absorption spectra were measured using quartz Hellma^®^ (Müllheim, Germany) square cuvettes on a Shimadzu UV-3600i Plus UV-vis-NIR spectrophotometer (Shimadzu, Kyoto, Japan) and baseline-corrected.

Steady-state excitation and emission spectra were recorded on a FluoTime 300 spectrometer from PicoQuant (Berlin, Germany), equipped with a 300 W ozone-free Xe lamp (250–900 nm), a 10 W Xe flash lamp (250–900 nm, pulse width < 10 µs) with repetition rates of 0.1–300 Hz, a single-grating excitation monochromator (Czerny-Turner 2.7 nm/mm dispersion, 1200 grooves/mm, blazed at 300 nm), diode lasers (pulse width < 80 ps) operated by a computer-controlled laser driver PDL-820 (repetition rate up to 80 MHz, burst mode for slow and weak decays), two single-grating emission monochromators (Czerny-Turner, selectable gratings blazed at 500 nm with 2.7 nm/mm dispersion and 1200 grooves/mm, or blazed at 1250 nm with 5.4 nm/mm dispersion and 600 grooves/mm), Glan–Thompson polarizers for excitation (Xe-lamps) and emission, a Peltier-thermostatized sample holder from Quantum Northwest (Washington, DC, USA) (−40–105 °C), and two detectors, namely, a PMA Hybrid 40 (transit time spread FWHM < 120 ps, 300–720 nm) and a R5509-42 NIR photomultiplier tube (transit time spread FWHM 1.5 ns, 300–1400 nm) with external cooling (−80 °C) from Hamamatsu (Shizuoka, Japan). Steady-state and fluorescence lifetimes were recorded in the TCSPC mode by a PicoHarp 300 (minimum base resolution 4 ps) from PicoQuant (Berlin, Germany). Emission and excitation spectra were corrected for source intensity (lamp and grating) by standard correction curves. Lifetime analysis was performed using the commercial EasyTau software (version 2.2) package from PicoQuant (Berlin, Germany). The quality of the fit was assessed by minimizing the reduced chi squared function (*χ*^2^) and through visual inspection of the weighted residuals and their autocorrelation. At least three independent batches of the compounds were synthesized and measured, with the samples undergoing multiple measurements to ensure data consistency and to mitigate measurement errors. Across these different batches, the results were consistently identical, underscoring the reliability of our findings.

### 3.4. Computational Details

All density functional theory (DFT) and time-dependent density functional theory (TD-DFT) calculations were performed using the quantum chemistry package Gaussian 09 Rev. D.01 [80]. Geometry optimizations in the ground state S_0_ were carried out with DFT, while TD-DFT was employed to obtain the optimized S_1_ and S_3_ excited state structures. The solvent dimethyl sulfoxide (DMSO) was modelled by the polarizable continuum model (PCM) in an integral equation formalism framework [81] with atomic radii from the universal force field model (UFF) [82]. In all cases, the spectral properties were calculated using the same functional as employed for geometry optimization.

UV-vis absorption spectra were calculated using the B3LYP functional [83], along with Grimme’s D3 dispersion correction with Becke–Johnson damping (BJ) [84] and the SDD basis set, which applies an effective core potential for the Zn atom [85] and the D95 basis set for H, C, N, and O atoms [86]. For each absorption spectrum, the 120 lowest excited singlet states were considered. A Lorentzian broadening with a half width of half maximum (HWHM) of 30 nm was used for each transition. 

The emission wavelengths were obtained from the difference in energy between the optimized ground state and the corresponding excited state geometries (0-0). In addition to the B3LYP functional, emission spectra were also calculated with the CAM-B3LYP [87], PBE0 [88], and M06-2X [89] functionals due to the delicate balance between local and charge-transfer excitations. A Lorentzian broadening with a half width of half maximum (HWHM) of 20 nm was used for the S_1_ → S_0_ transition, while a value of 60 nm was applied for the S_3_ → S_0_ transition to match the experimental line shape.

### 3.5. MSOT Photoacoustic Imaging

Phantom photoacoustic imaging experiments were performed using a Multispectral Optoacoustic Tomography system (MSOT inVision 512-echo, iThera Medical, Munich, Germany). The phantom consists of a cylinder of 1.5% agarose with 2% of 20% soybean cream as scattering medium and 0.75% 2.5 mM Nigrosin, which was cast, including 2 straws as placeholders. Once hardened, these placeholders were removed and replaced with sealed straws, which had been filled with test solution and a control containing only the solvent. Multispectral photoacoustic imaging of phantoms containing the probe and control samples were performed tomographically for the length of the straw using an excitation wavelength ranging from 680 to 900 nm in steps of 5 nm. Images were loaded into an in-house developed image processing software, MEDgical (Version 0.9.9, EIMI, Münster, Germany). Spectra were exported and processed to create the presented plots.

### 3.6. Fluorescence Lifetime Imaging Microscopy (FLIM) by Time-Resolved Multiphoton Micro(spectro)scopy

Fluorescence lifetime imaging microscopy (FLIM) was recorded on a fluorescence microscope IX 73 from Olympus (Shinjuku, Japan) with a complete confocal system and a laser-combining unit (LCU), an inverted microscope body, and a multichannel detection unit, namely a MicroTime 200 from PicoQuant (Berlin, Germany) equipped with diode lasers (providing adjustable output power and repetition rates up to 80 MHz inside a compact fiber couple unit with wavelengths between 375 and 900 nm). For beam diagnostics, a charge-coupled device (CCD) camera and a photodiode were available in the main optical unit (MOU) of the microscope. The MOU was equipped with two detectors, namely, a hybrid photomultiplier-based single photon counting module (PMA Hybrid 40, PicoQuant) and an SPAD-based photon-counting module (SPCM-AQR-14, Perkin-Elmer, Hopkinton, MA, USA). Different band-pass (BP) and low-pass (LP) filters were placed before these detectors on demand to acquire lifetime maps. Data acquisition was based on the unique time-tagged time-resolved (TTTR) measurement mode, where simultaneous data acquisition on two channels is possible. Data were processed and analyzed with the SymphoTime 64 (PicoQuant) software. In order to couple the MicroTime 200 and the FluoTime 300 instruments (both from PicoQuant, Berlin, Germany), a fiber coupler was employed. In this way, the spectrometer can be used to record either steady-state or time-resolved luminescence spectra and decays from a sample mounted on the microscope. Luminescence micrographs were acquired using the same microscope mentioned above, equipped with an X-CiteQ Lamp module (Excelitas Technologies, Waltham, MA, USA) as excitation source and a UI-5580SE (IDS) digital camera. Different band-pass (BP) and low-pass (LP) cubes were using accordingly.

Additionally, a two-photon (Mai Tai^®^ from SpectraPhysics, Darmstadt, Germany) Ti:Sapphire laser (Ti:Sa oscillator) with a pulse with <100 fs and tuning range of 690–1040 nm, connected to the MOU, can be used as excitation source. In order to reduce the repetition rate, the Ti:Sapphire laser was connected to a double pulse picker (A.P.E.^®^, Berlin, Germany).

## 4. Conclusions

In conclusion, we report on a set of novel Zn(II) naphthalocyaninates with a prominent dual fluorescence encompassing the visible and the NIR portion of the electromagnetic spectrum. A proper synthesis, purification, and full characterization of the peripherally octa-substituted zinc(II) naphthalocyaninato complexes **Zn-OMeNc**, **Zn-NMe_2_Nc**, and **Zn-NMe_3_Nc** was undertaken for the first time; hence, a detailed structural elucidation based on NMR studies is provided. The introduction of a push–pull system on the macrocycle enabled the integration of two orthogonal chromophores with unprecedented dual emission, which was assigned by means of TD-DFT calculations. Upon quaternization of **Zn-NMe_2_Nc**, we produced the first to-date reported octa-cationic water-soluble zinc(II) naphthalocyaninate, namely, **Zn-NMe_3_Nc**. The quaternized species, **Zn-NMe_3_Nc**, exhibited excellent solubility in water; even though aggregation phenomena can be traced in aqueous media, stacking is suppressed in DMSO. Furthermore, due to its good absorption in the near-infrared region, we also investigated the photoacoustic imaging capability of **Zn-NMe_2_Nc** and **Zn-NMe_3_Nc**. The photoacoustic profiles of these Ncs, studied in gel phantoms, show a high intensity and red-shifted photoacoustic maxima in a broad concentration range. Among **Zn-NMe_2_Nc** and **Zn-NMe_3_Nc**, the best photoacoustic performance was observed for **Zn-NMe_3_Nc** in DMSO, particularly in terms of its detectability at lower concentrations. Staining microparticles with **Zn-NMe_2_Nc** revealed that the additional readout in the visible range stemming from the side groups enables time-resolved multiphoton micro(spectro)scopy. This holds true even when aggregation quenches the intrinsic fluorescence of the macrocycle. Since well-defined purification and characterization techniques for Ncs are sparsely found in the literature, this work could expand the possibilities of water-soluble dyes for PDT and diagnostics. More generally, the unprecedented dual emission (visible and NIR) unlocks the prospective use of naphthalocyanines for multiscale–multimodal bioimaging and for applications in sensing or optoelectronics. 

## Figures and Tables

**Table 1:** Q- and B-bands observed in the absorption spectra. The corresponding molar absorption coefficient (*ε*) for each band is also listed in square brackets.

Compound/ Solvent	Q-Band (*λ*_max_/nm) [*ε*/10^4^ M^−1^ cm^−1^]	B-Band (*λ*_max_/nm) [*ε*/10^5^ M^−1^ cm^−1^]
**Zn-OMeNc**/DMSO	794 [4.4]	293 [1.9]
**Zn-NMe_2_Nc**/DMSO	800 [5.3]	350 [2.0]
**Zn-NMe_3_Nc**/DMSO	781 [55]	350 [1.8]
**Zn-NMe_3_Nc**/H_2_O	732, 784 [22], [18]	340 [2.7]

**Table 2:** Photophysical properties of the compounds (*λ*_max_ and *τ*). P1 and P2 are the peaks in the visible and NIR regions in the emission spectra, respectively. For the bi-exponential photoluminescence decays, the amplitude-weighted average lifetimes (*τ*_av_amp_) are shown. The *τ* values are rounded to match the significant figures of the uncertainties; raw time-resolved photoluminescence decays and fitting parameters are shown in the SI, Figures S48–S55.

Compound/ Solvent	*λ*_max_/nm P1/P2	*τ*/ns [P1]	*τ*/ns [P2]
**Zn-OMeNc**/DMSO	508/804	*τ*_1_ = 8.2 ± 0.3 (21%) *τ*_2_ = 2.7 ± 0.1 (79%) *τ*_av_amp_ = 3.8 ± 0.1	*τ*_1_ = 2.05 ± 0.03 (68%) τ_2_ = 1.0 ± 0.1 (32%) *τ*_av_amp_ = 1.7 ± 0.1
**Zn-NMe_2_Nc**/DMSO	560/811	*τ*_1_ = 8.4 ± 0.2 (34%) *τ*_2_ = 2.3 ± 0.2 (61%) *τ*_av_amp_ = 4.6 ± 0.1	*τ*_1_ = 1.80 ± 0.08 (21%) *τ*_2_ = 0.61 ± 0.03 (79%) *τ*_av_amp_ = 0.86 ± 0.04
**Zn-NMe_3_Nc**/DMSO	550/801	*τ*_1_ = 9.1 ± 0.2 (55%) *τ*_2_ = 3.4 ± 0.4 (45%) *τ*_av_amp_ = 6.6 ± 0.2	*τ* = 5.69 ± 0.02
**Zn-NMe_3_Nc**/H_2_O	432/804	*τ*_1_ = 8.5 ± 0.4 (9%) *τ*_2_ = 5.2 ± 0.1 (91%) *τ*_av_amp_ = 5.51 ± 0.03	*τ*_1_ = 5.43 ± 0.05 (28%) *τ*_2_ = 1.20 ± 0.08 (72%) *τ*_av_amp_ = 2.4 ± 0.1

## Data Availability

The data that support the findings of this study are available in the Supplementary Material of this article.

## Supplementary Materials

The following supporting information can be downloaded at: https://www.mdpi.com/article/10.3390/ijms25052605/s1.
