# Supplementary material for: Dual Emissive Zn(II) Naphthalocyanines: Synthesis, Structural and Photophysical Characterization with Theory-Supported Insights towards Soluble Coordination Compounds with Visible and Near-Infrared Emission
Source: Int J Mol Sci. 2024 Feb 23;25(5):2605. doi: 10.3390/ijms25052605 (PMC10931793; doi:10.3390/ijms25052605)
Supplement: Supplementary file 1 [file ijms-25-02605-s001.zip › ijms-2828144-supplementary.pdf]

# Dual Emissive Zn(II) Naphthalocyanines: Synthesis, Structural and Photophysical Characterization with Theory-Supported Insights towards Soluble Coordination Compounds with Visible and Near-Infrared Emission

Sidharth Thulaseedharan Nair Sailaja 1,2 , Iván Maisuls 1,2, Alexander Hepp 1 , Dana Brünink 3,
Nikos L. Doltsinis 3 , Andreas Faust 4,5, Sven Hermann 4,5 and Cristian A. Strassert 1,2,*
1 Institut für Anorganische und Analytische Chemie, Universität Münster, Corrensstraße 28/30,
48149 Münster, Germany; maisuls@uni-muenster.de (I.M.); alexander.hepp@uni-muenster.de (A.H.)
2 CeNTech, CiMIC, SoN, Universität Münster, Heisenbergstraße 11, 48149 Münster, Germany
3 Institute for Solid State Theory and Center for Multiscale Theory and Computation, Universität Münster,
Wilhelm-Klemm-Straße 10, 48149 Münster, Germany
4 European Institute for Molecular Imaging, Universität Münster, Röntgenstraße 16, 48149 Münster, Germany;
faustan@uni-muenster.de (A.F.)
5 Department of Nuclear Medicine, University Hospital Münster, Albert-Schweitzer-Campus 1,
48149 Münster, Germany
* Correspondence: ca.s@uni-muenster.de

# Table of contents

**Section S1:** NMR spectra of **OMe**, **$NMe_2$**, **Zn-OMeNc**, **Zn-$NMe_2$Nc**, **Zn-$NMe_3$Nc** **03**

**Section S2:** Mass spectra of **OMe**, **$NMe_2$**, **Zn-OMeNc**, **Zn-$NMe_2$Nc**, **Zn-$NMe_3$Nc** **16**

**Section S3:** Photophysical characterization of **OMe**, **$NMe_2$**, **Zn-OMeNc**, **Zn-$NMe_2$Nc**, **Zn-$NMe_3$Nc** **18**

**Section S4:** DFT calculations **30**

**Section S5: Zn$NMe_2$Nc** loaded onto polystyrene microparticles (PSMPs) **33**

## Section S1: NMR spectra of OMe, NMe$_2$, Zn-OMeNc, Zn-NMe$_2$Nc, Zn-NMe$_3$Nc

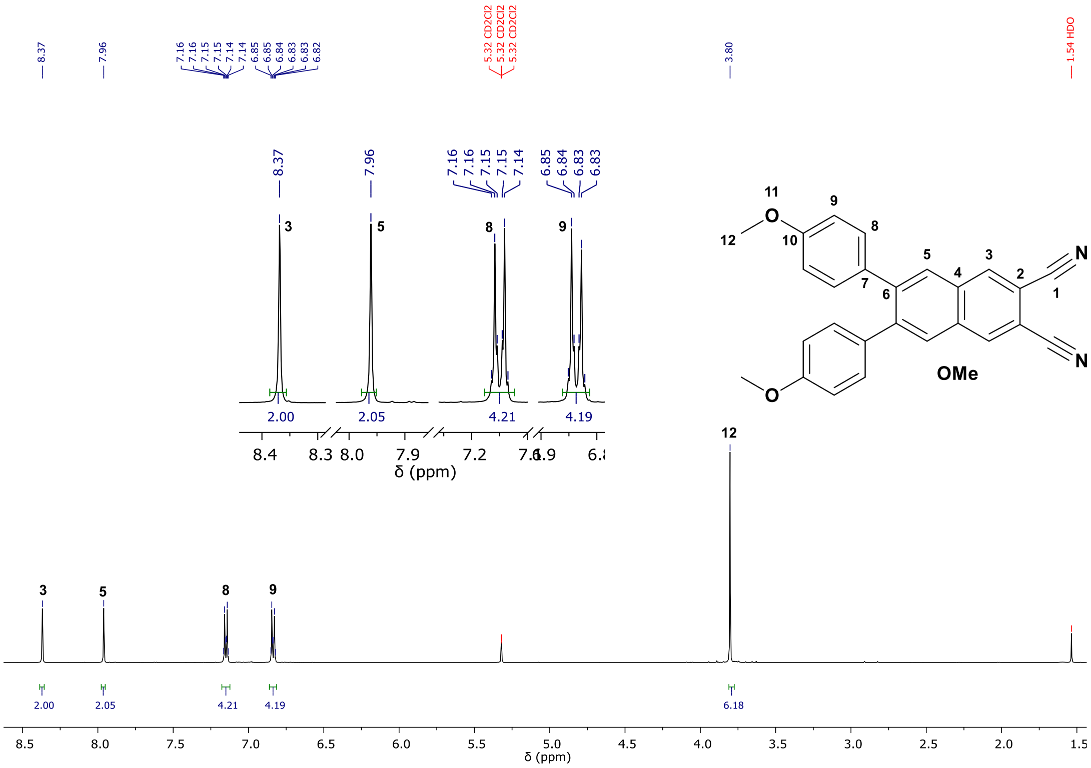

**Fig. S1** $^{1}$H-NMR spectrum (500 MHz, DCM-$d_2$) of **OMe**.

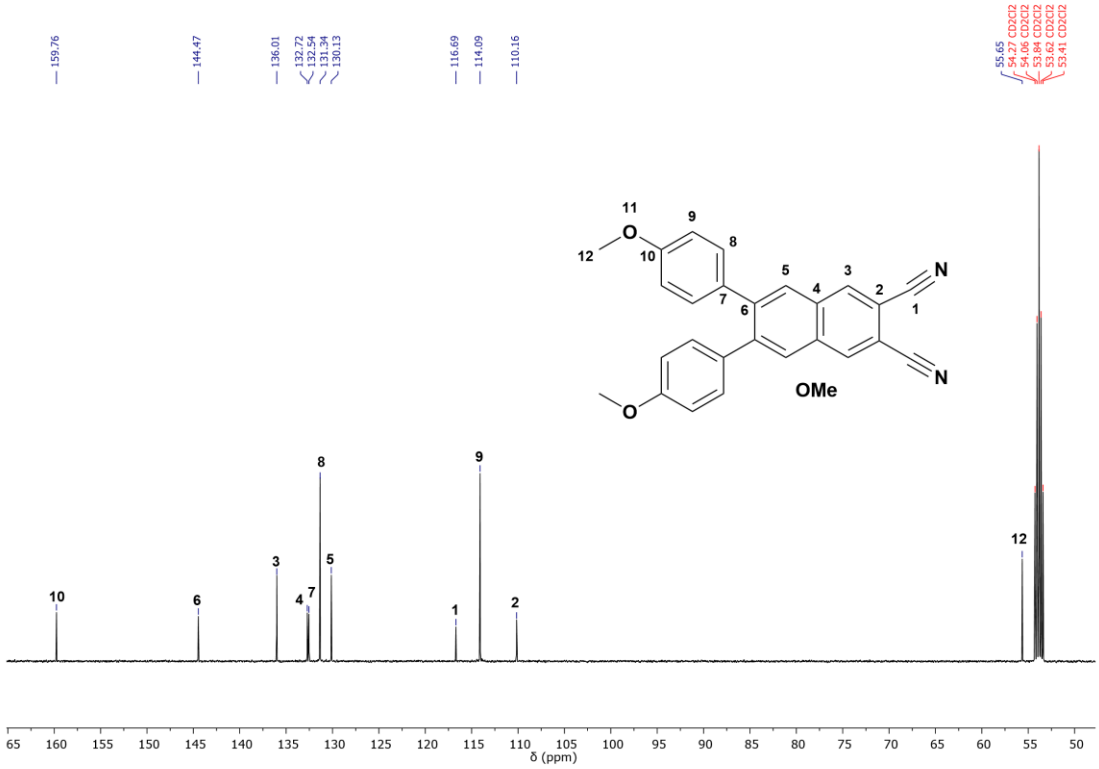

**Fig. S2** $^{13}$C-{$^{1}$H}-NMR spectrum (126 MHz, DCM-$d_2$) of **OMe**).

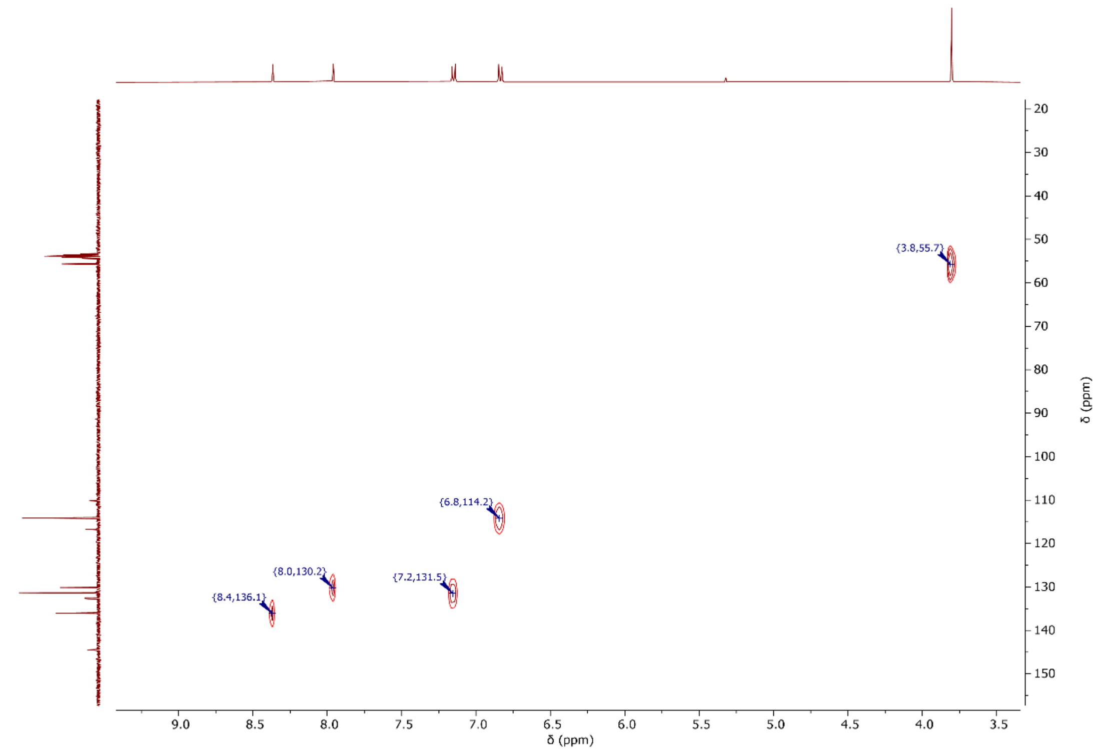


**Fig. S3** $^{1}H/^{13}C$-gHSQC-NMR spectrum (400 MHz/101 MHz, DCM-$d_2$) of **OMe**.

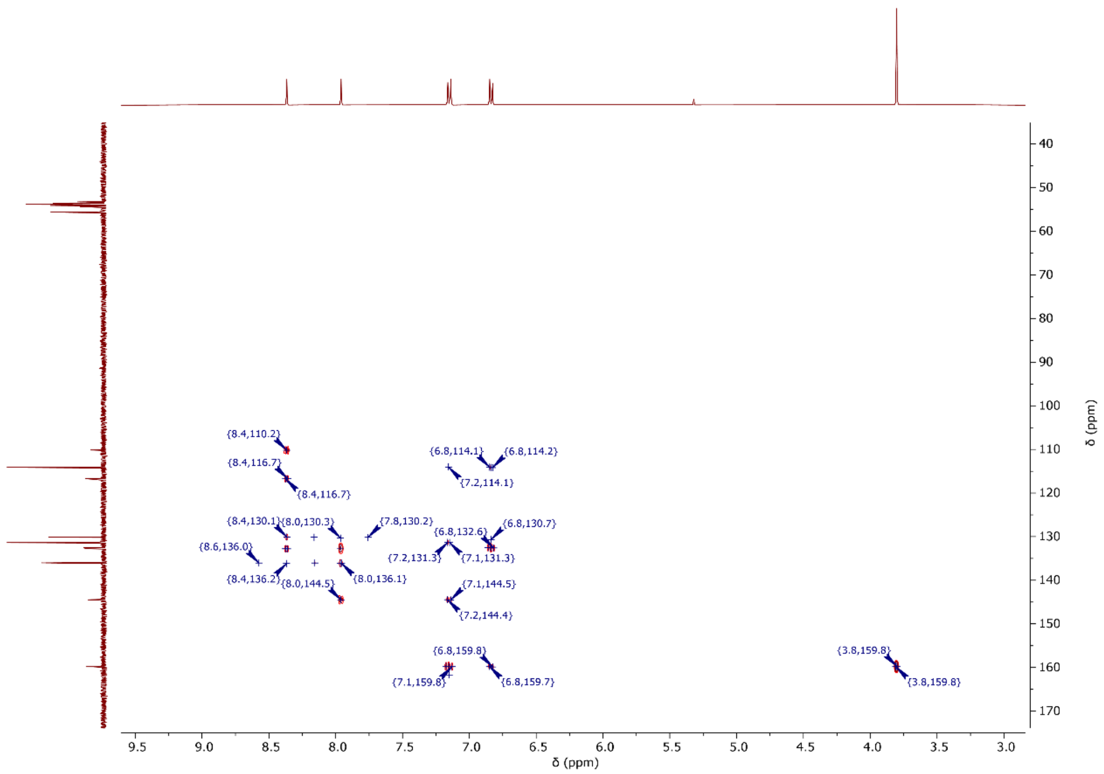


**Fig. S4** $^{1}H/^{13}C$-gHMBC-NMR spectrum (400 MHz/101 MHz, DCM-$d_2$) of **OMe**.

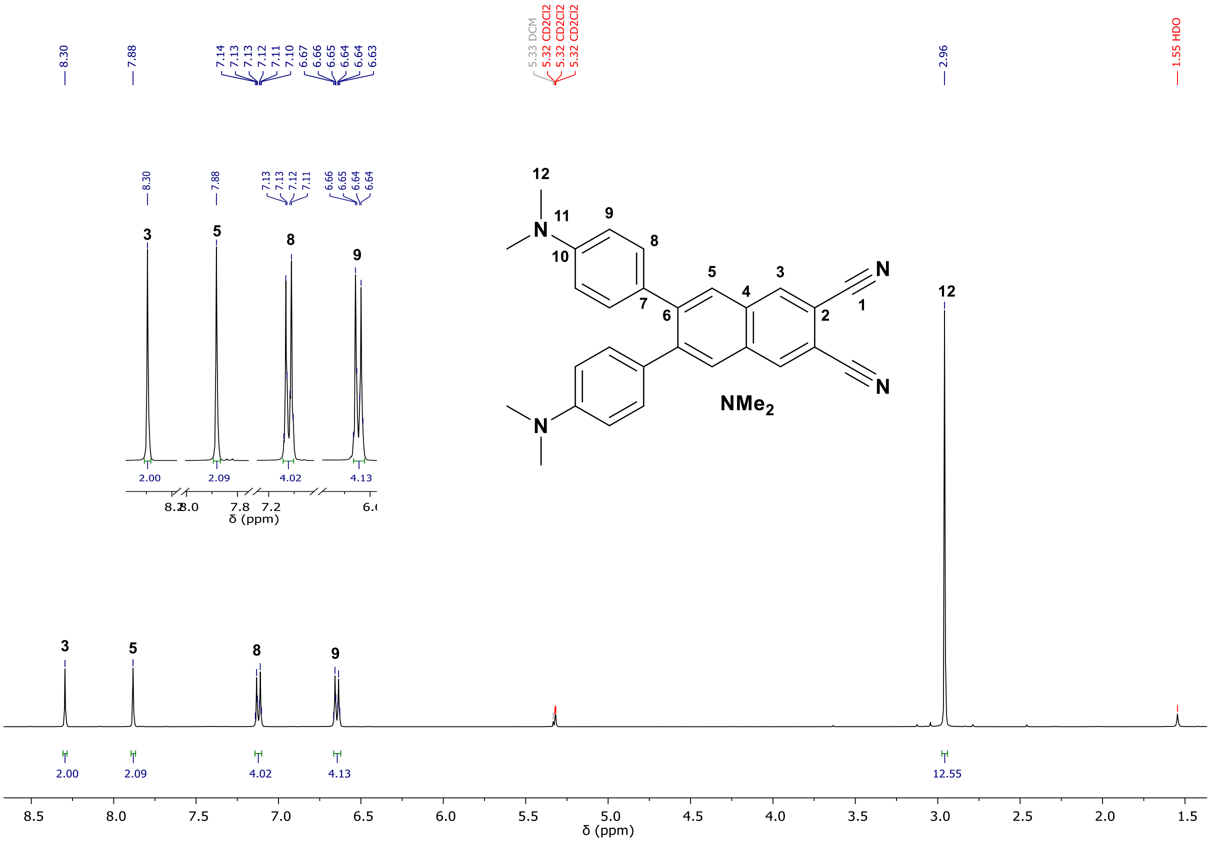

**Fig. S5** $^{1}$H-NMR spectrum (500 MHz, DCM-$d_2$) of **NMe$_2$**.

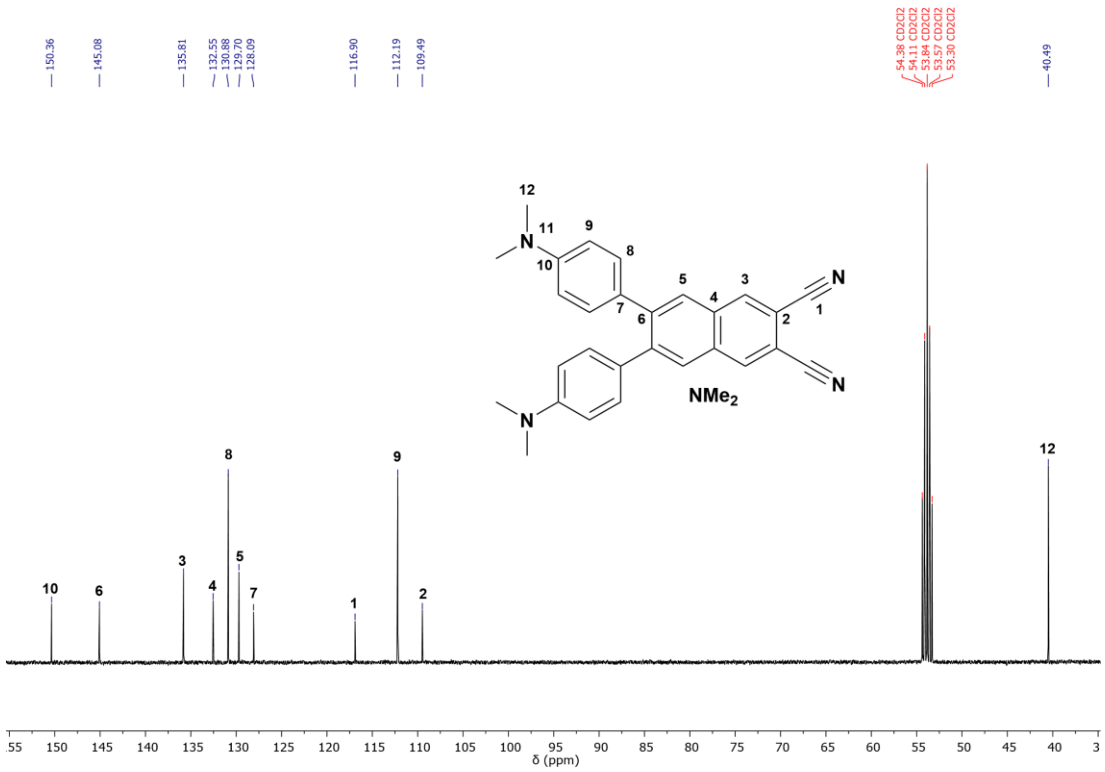

**Fig. S6** $^{13}$C-{$^{1}$H}-NMR spectrum (126 MHz, DCM-$d_2$) of **NMe$_2$**.

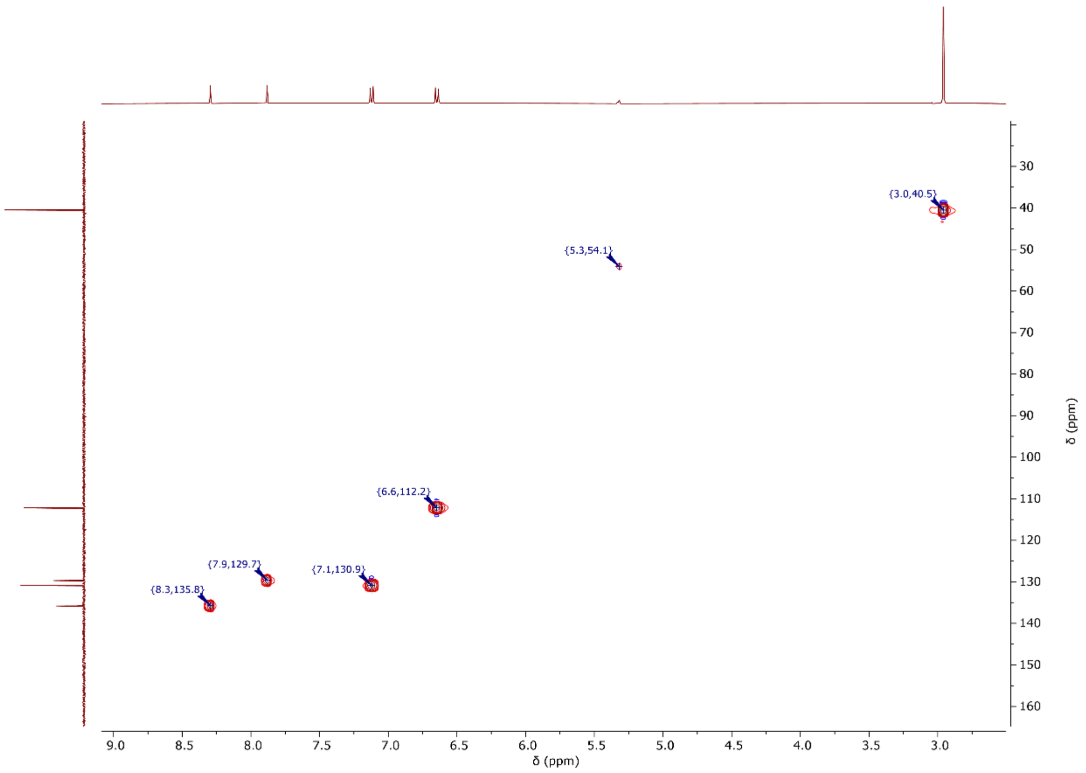


**Fig. S7** $^{1}H/^{13}C$-gHSQC-NMR spectrum (400 MHz/101 MHz, DCM-$d_2$) **NMe₂**.

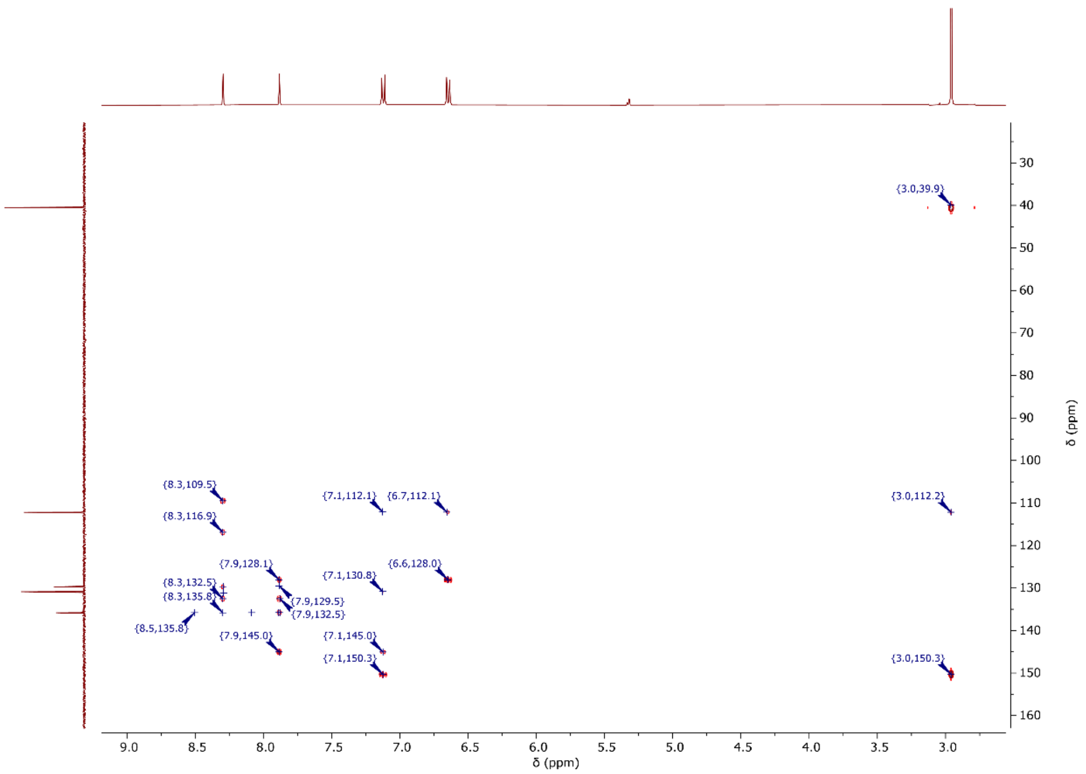


**Fig. S8** $^{1}H/^{13}C$-gHMBC-NMR spectrum (400 MHz/101 MHz, DCM-$d_2$) of **NMe₂**.

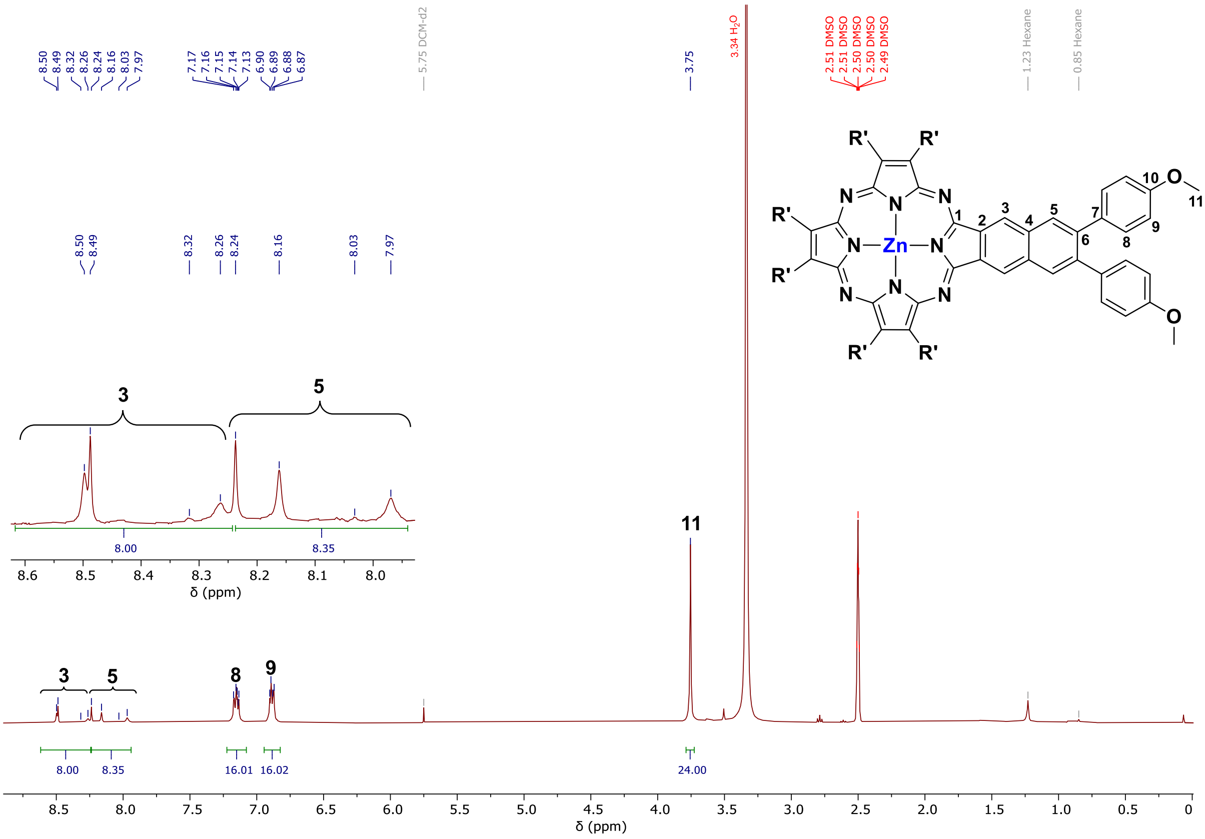

**Fig. S9** $^{1}$H-NMR spectrum (400 MHz, DMSO-$d_6$) of **Zn-OMeNc**.

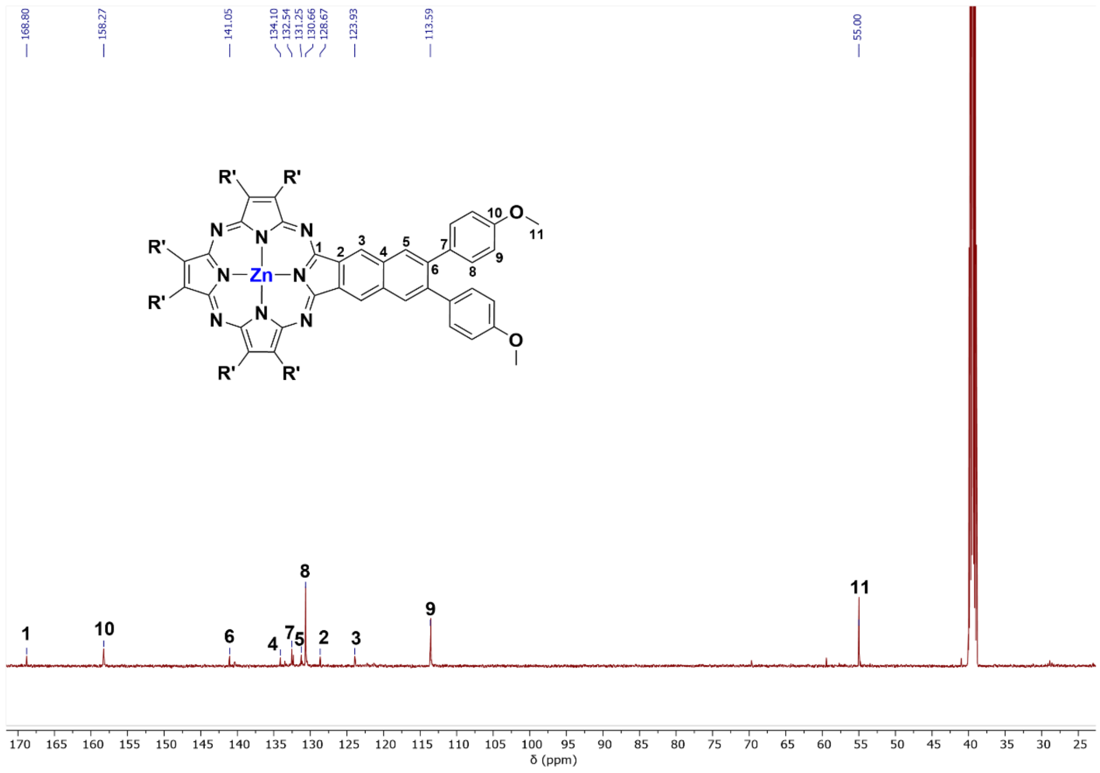

**Fig. S10** $^{13}$C-{$^{1}$H}-NMR spectrum (126 MHz, DMSO-$d_6$) of **Zn-OMeNc.**

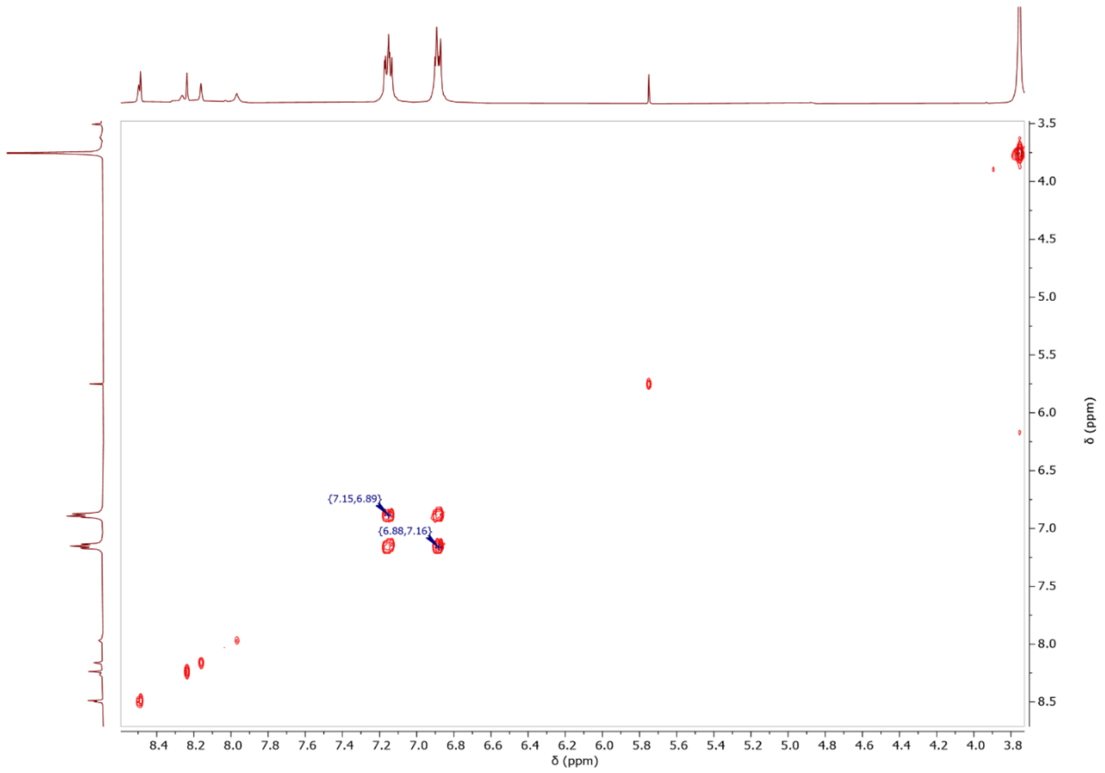


**Fig. S11** $^{1}H$-$^{1}H$-COSY-NMR spectrum (500 MHz, DMSO-$d_6$) of **Zn-OMeNc**.

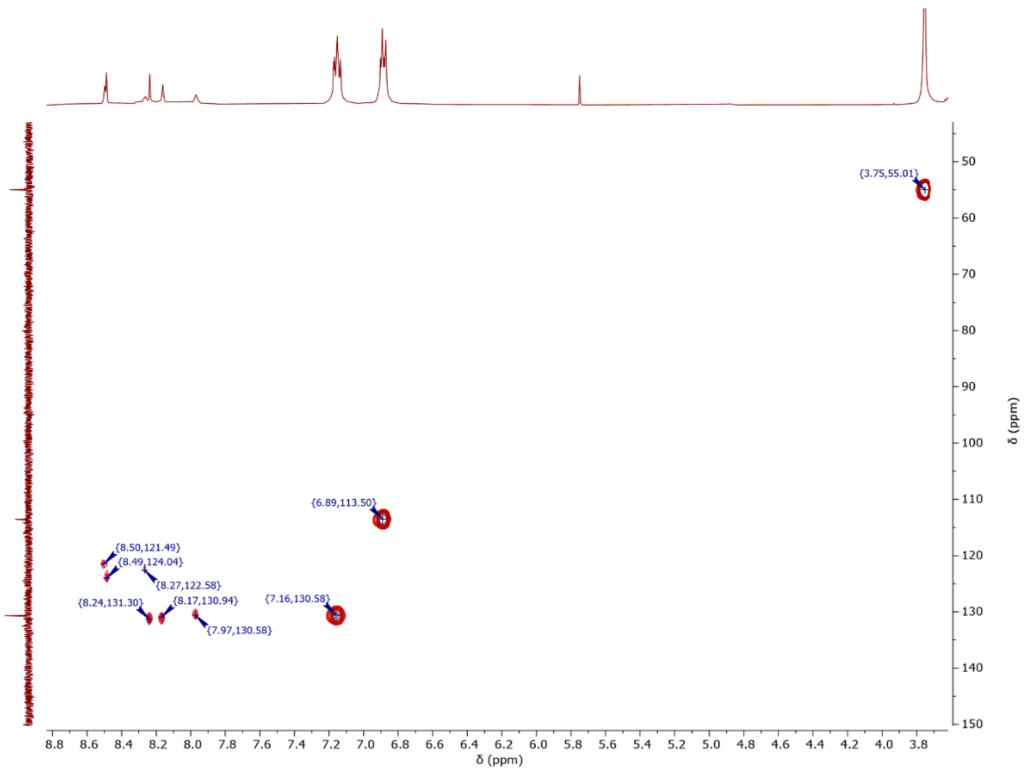


**Fig. S12** $^{1}H$/$^{13}C$-gHSQC-NMR spectrum (400 MHz/101 MHz, DMSO-$d_6$) of **Zn-OMeNc**.

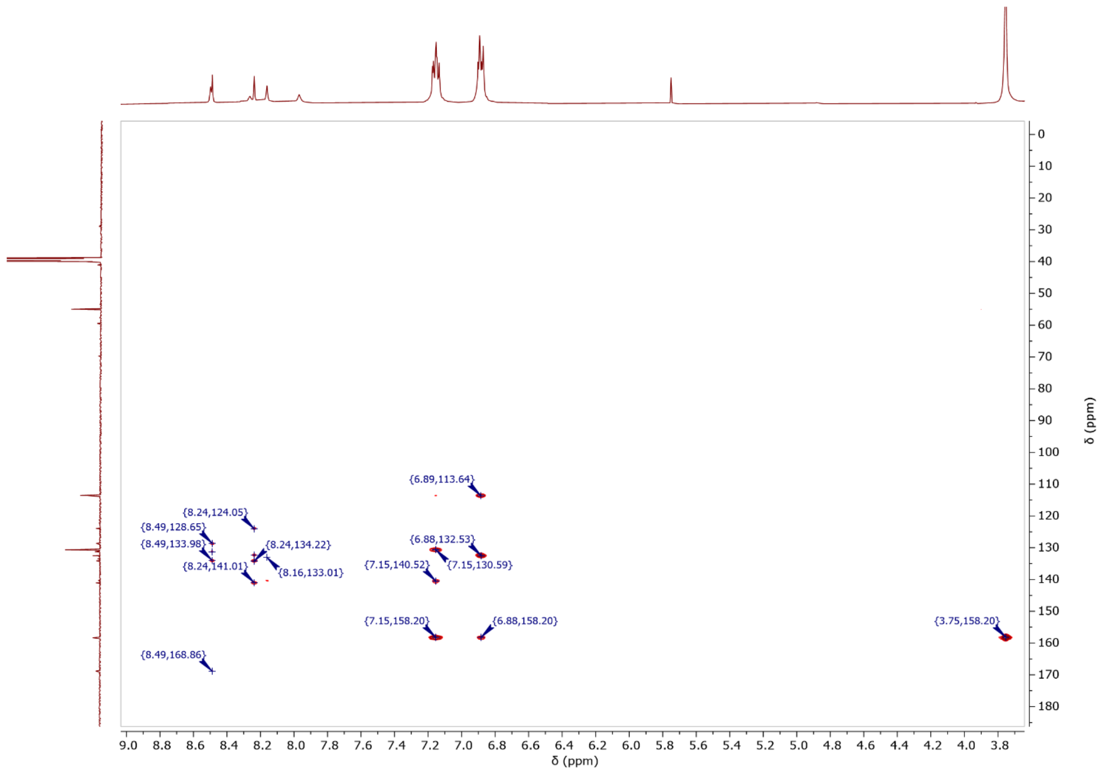


**Fig. S13** $^{1}H/^{13}C$-gHMBC-NMR spectrum (400 MHz/101 MHz, DMSO-$d_6$) of **Zn-OMeNc**.

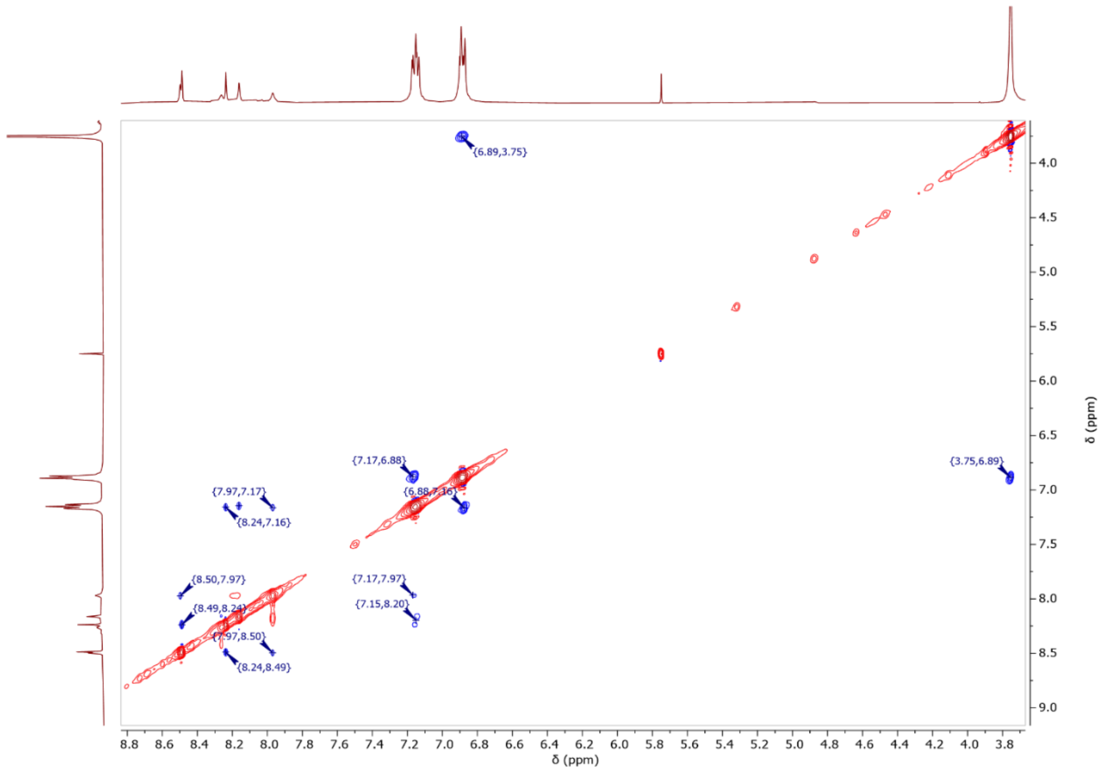


**Fig. S14** $^{1}H$-$^{1}H$-ROESY-NMR spectrum (500 MHz, DMSO-$d_6$) of **Zn-OMeNc**.

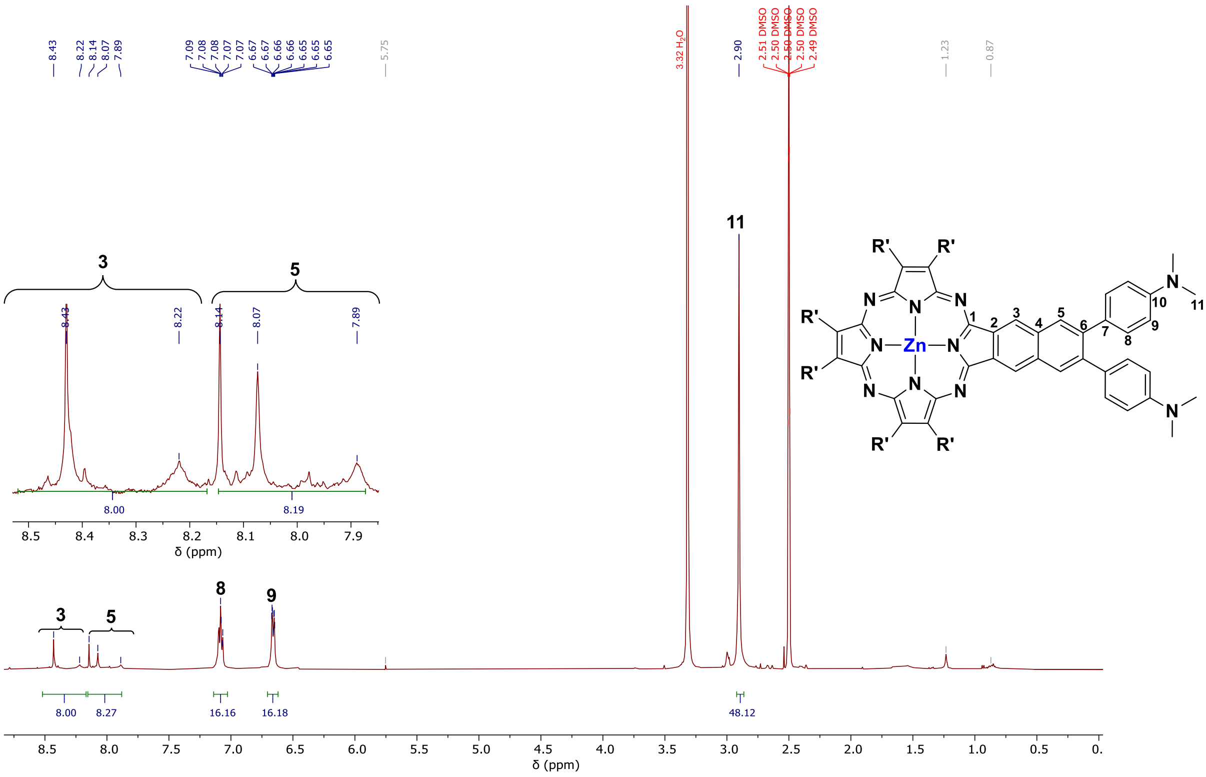

**Fig. S15** $^{1}$H-NMR spectrum (400 MHz, DMSO-$d_6$) of **Zn-NMe$_2$Nc**.

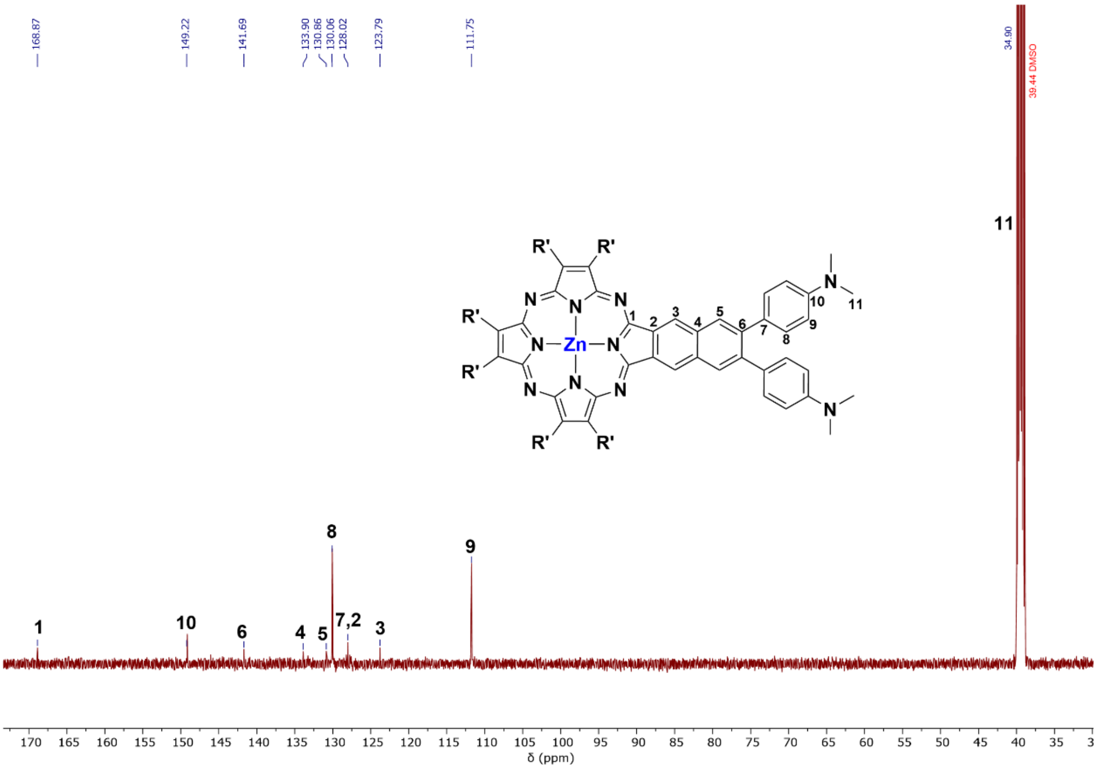

**Fig. S16** $^{13}$C-{$^{1}$H}-NMR spectrum (100 MHz, DMSO-$d_6$) of **Zn-NMe$_2$Nc**.

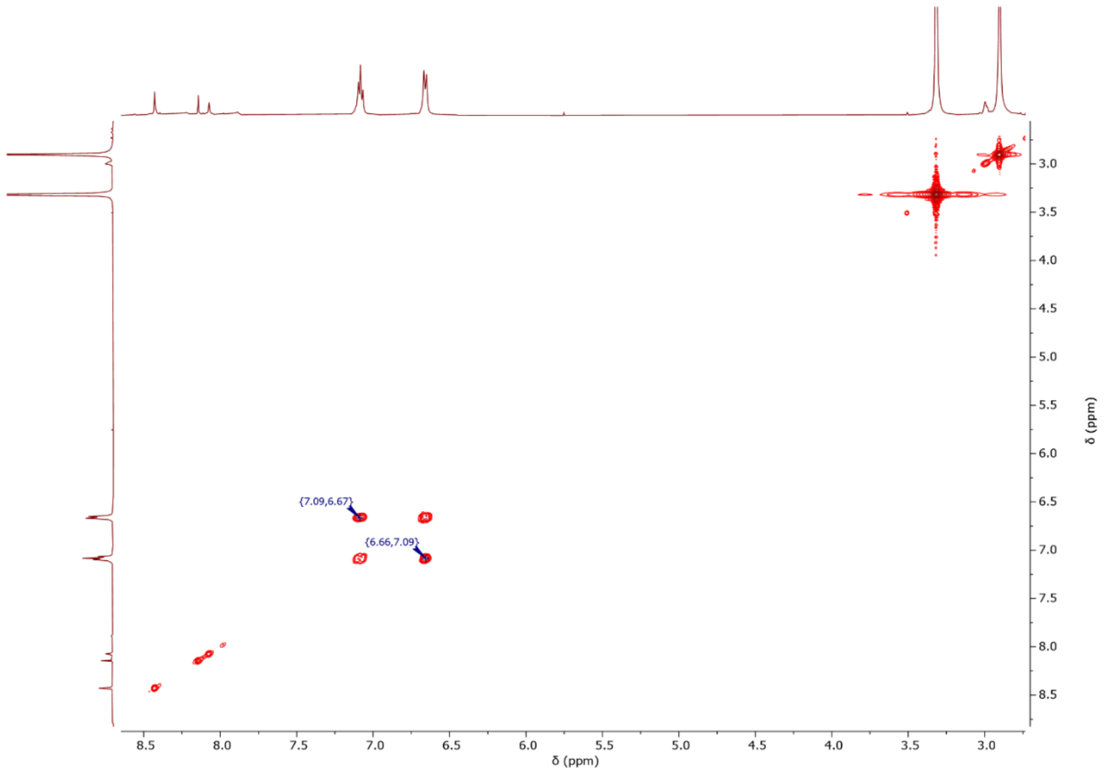


**Fig. S17** $^{1}$H-$^{1}$H-COSY-NMR spectrum (400 MHz, DMSO-*d*$_6$) of **Zn-NMe$_2$Nc**.

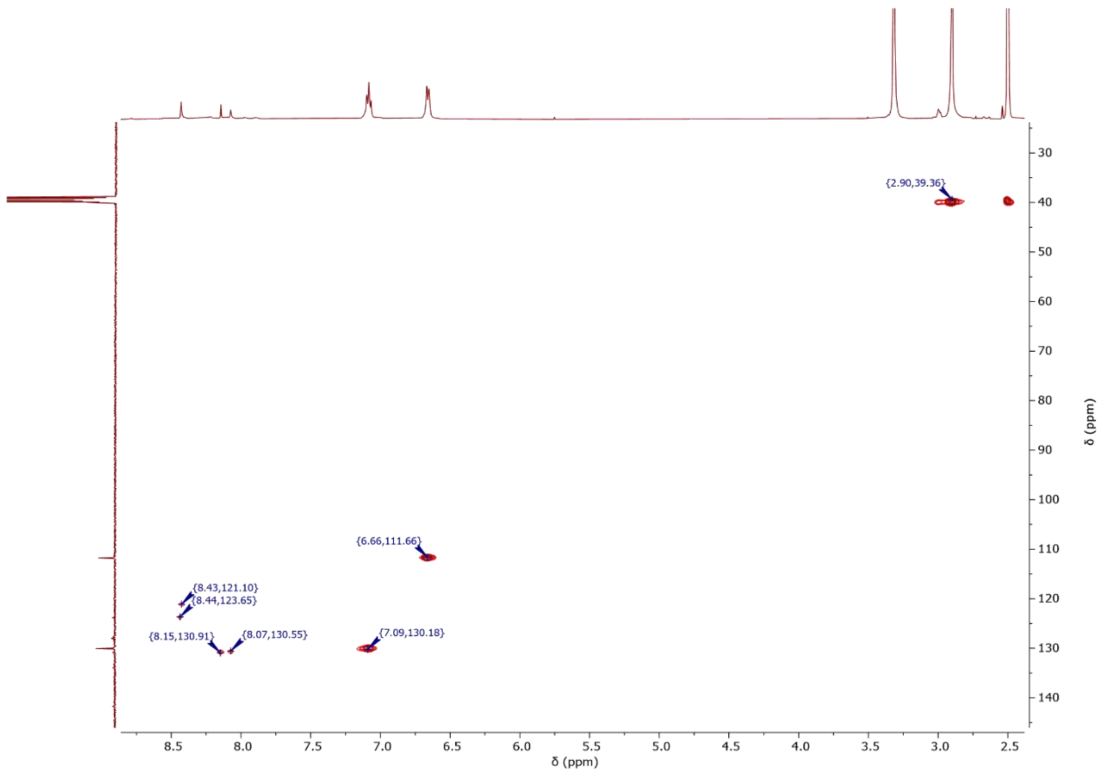


**Fig. S18** $^{1}$H/$^{13}$C-gHSQC-NMR spectrum (400 MHz, DMSO-*d*$_6$) of **Zn-NMe$_2$Nc**.

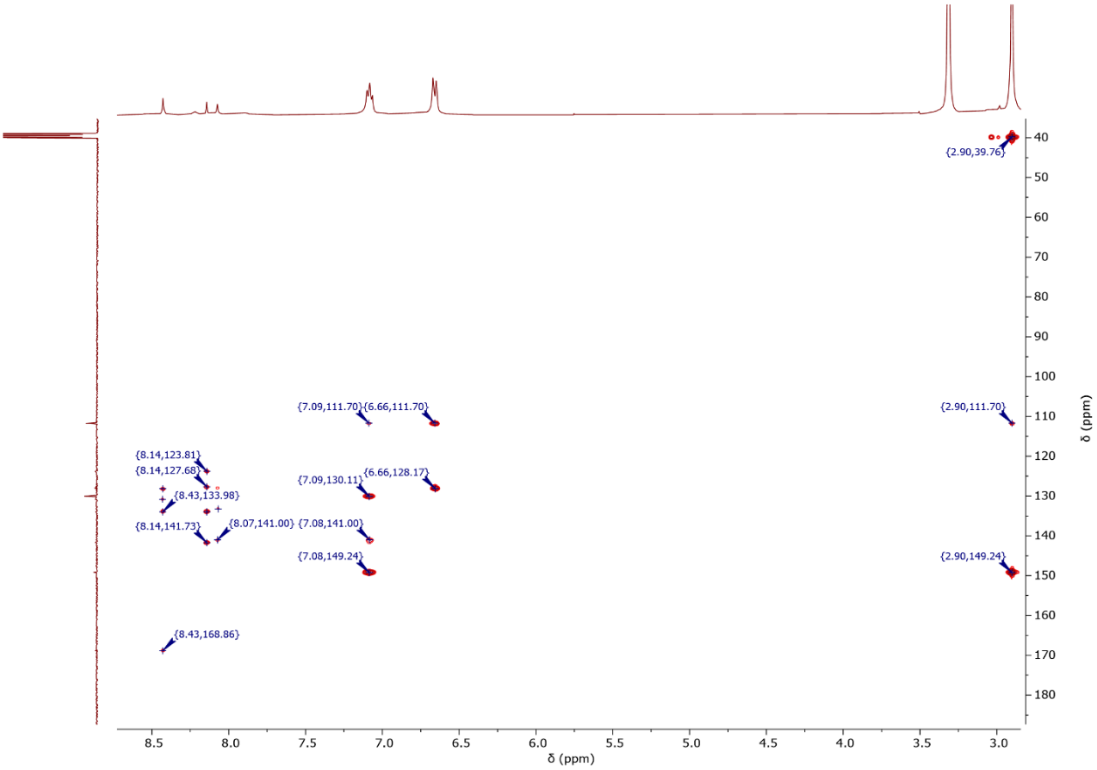


**Fig. S19** $^{1}H/^{13}C$-gHMBC-NMR spectrum (400 MHz, DMSO-$d_6$) of **Zn-NMe$_2$Nc**.

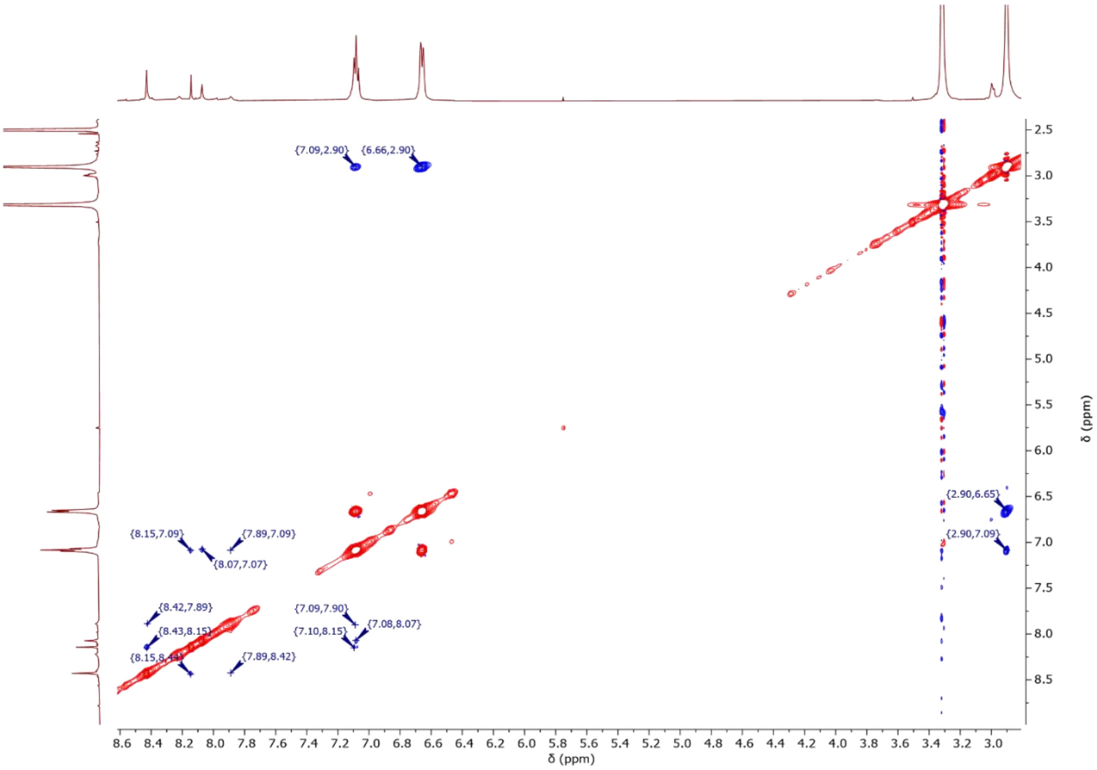


**Fig. S20** $^{1}H$-$^{1}H$-ROESY-NMR spectrum (400 MHz, DMSO-$d_6$) of **Zn-NMe$_2$Nc**.

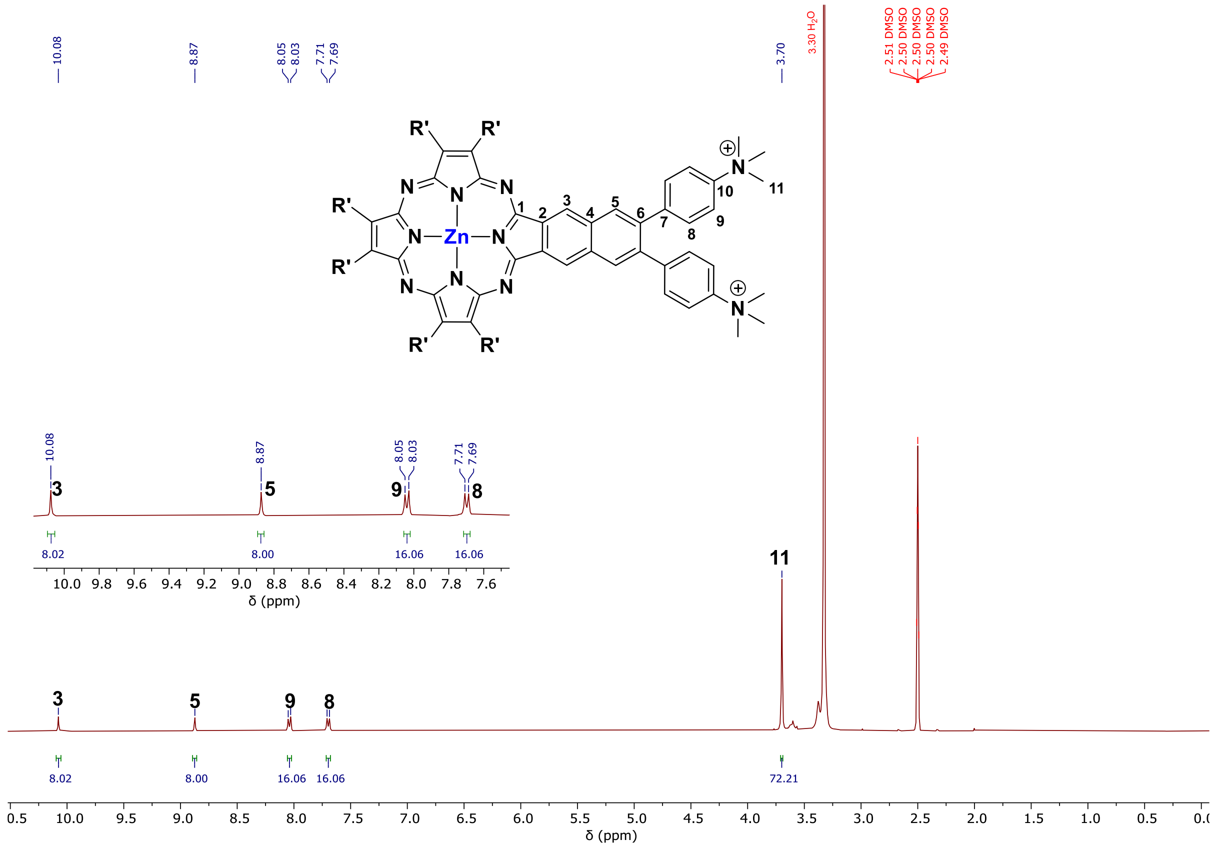


**Fig. S21** $^{1}$H-NMR spectrum (400 MHz, DMSO-$d_6$) of **Zn-NMe$_3$Nc**.

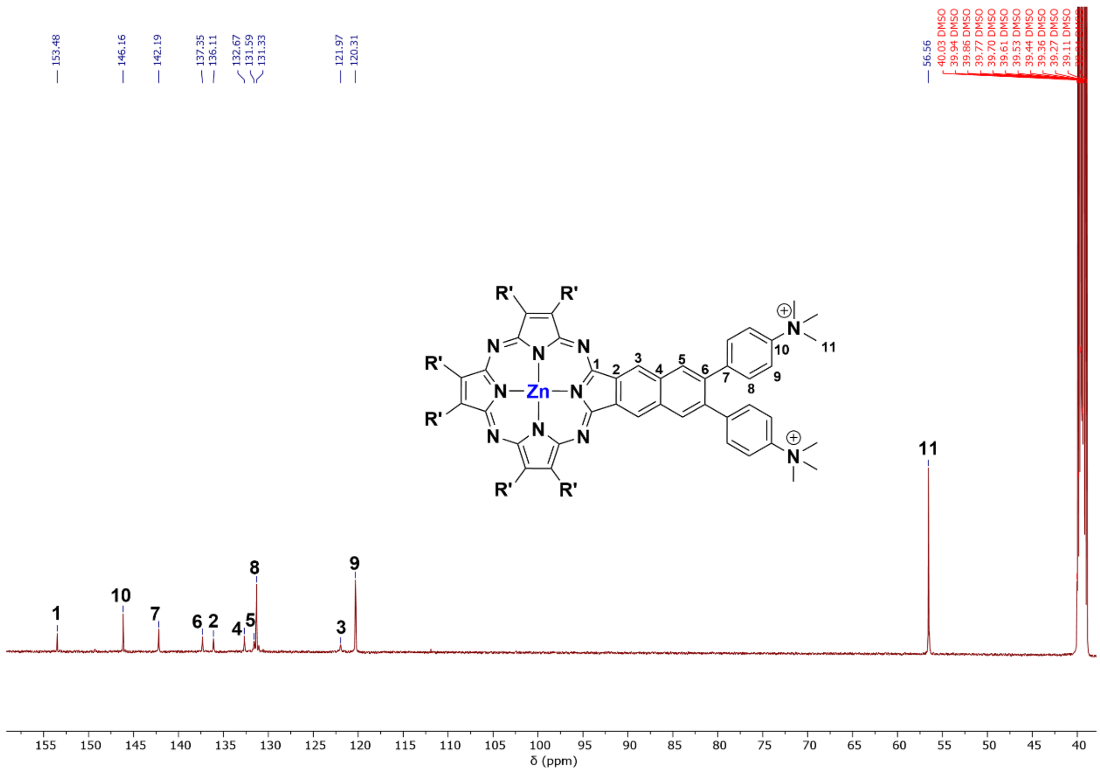


**Fig. S22** $^{13}$C-{$^{1}$H}-NMR spectrum (126 MHz, DMSO-$d_6$) of **Zn-NMe$_3$Nc**.

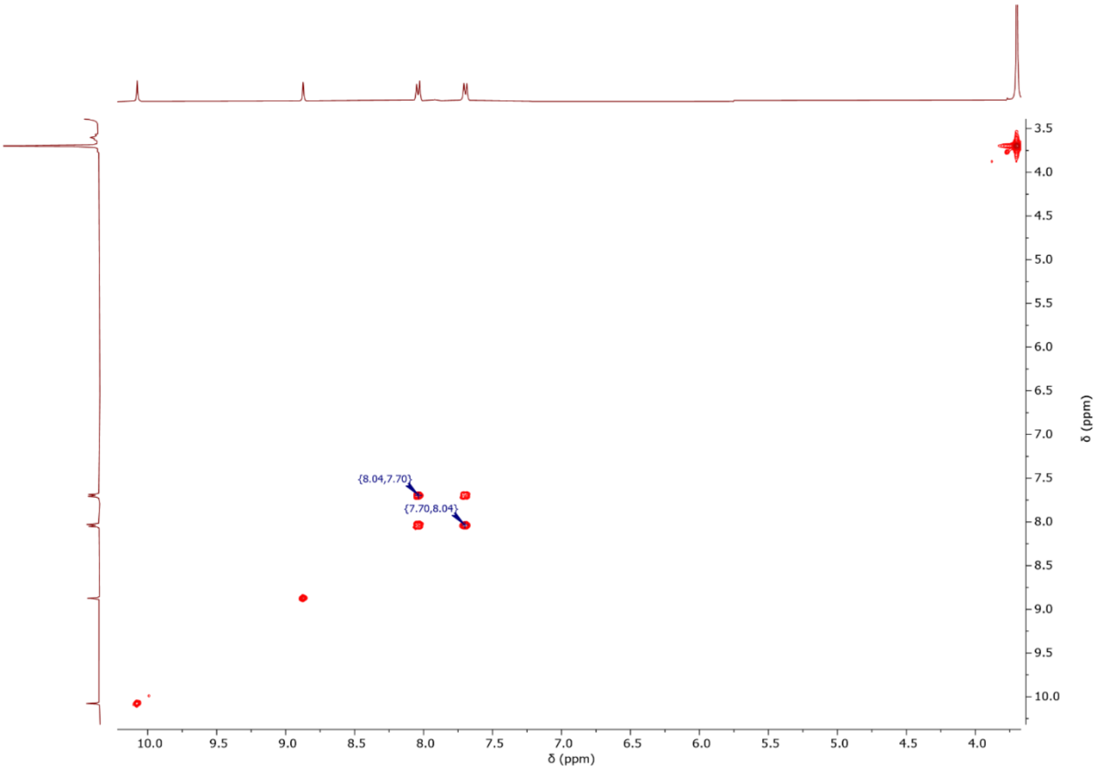


**Fig. S23** $^{1}H$-$^{1}H$-COSY-NMR spectrum (400 MHz, DMSO-$d_6$) of **Zn-NMe$_3$Nc**.

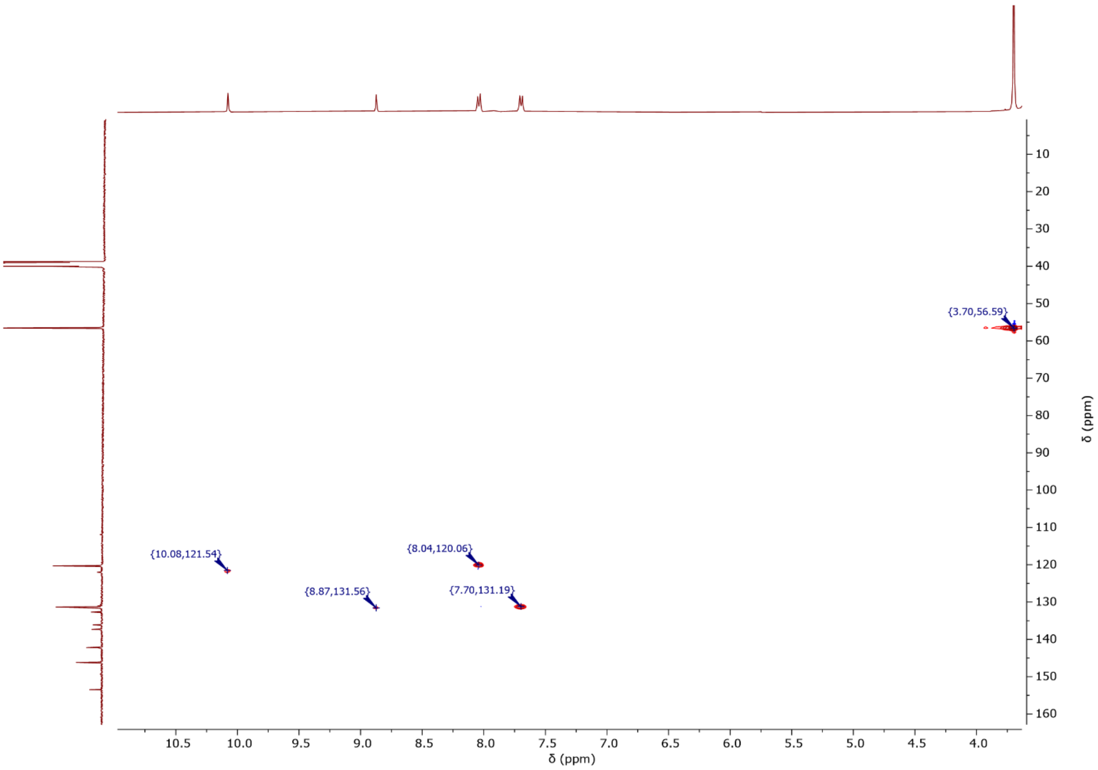


**Fig. S24** $^{1}H$/$^{13}C$-gHSQC-NMR spectrum (400 MHz/101 MHz, DMSO-$d_6$) of **Zn-NMe$_3$Nc**.

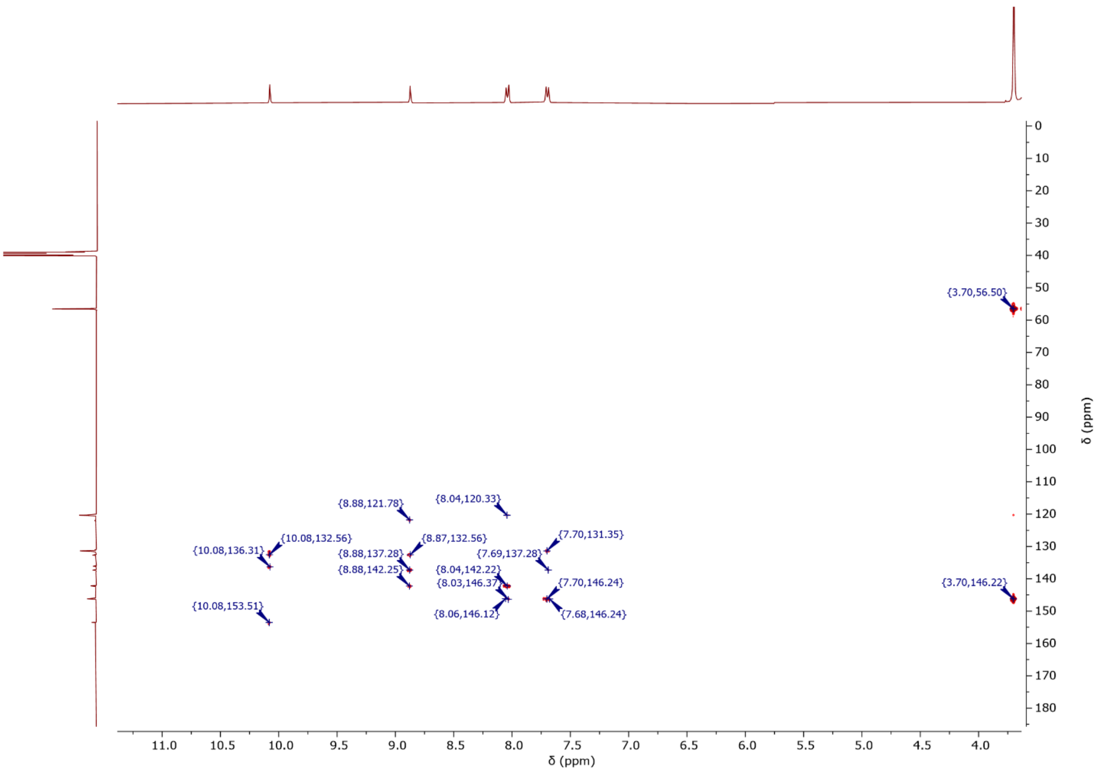


**Fig. S25** $^{1}H/^{13}C$-gHMBC-NMR spectrum (400 MHz/101 MHz, DMSO-$d_6$) of **Zn-NMe$_3$Nc**.

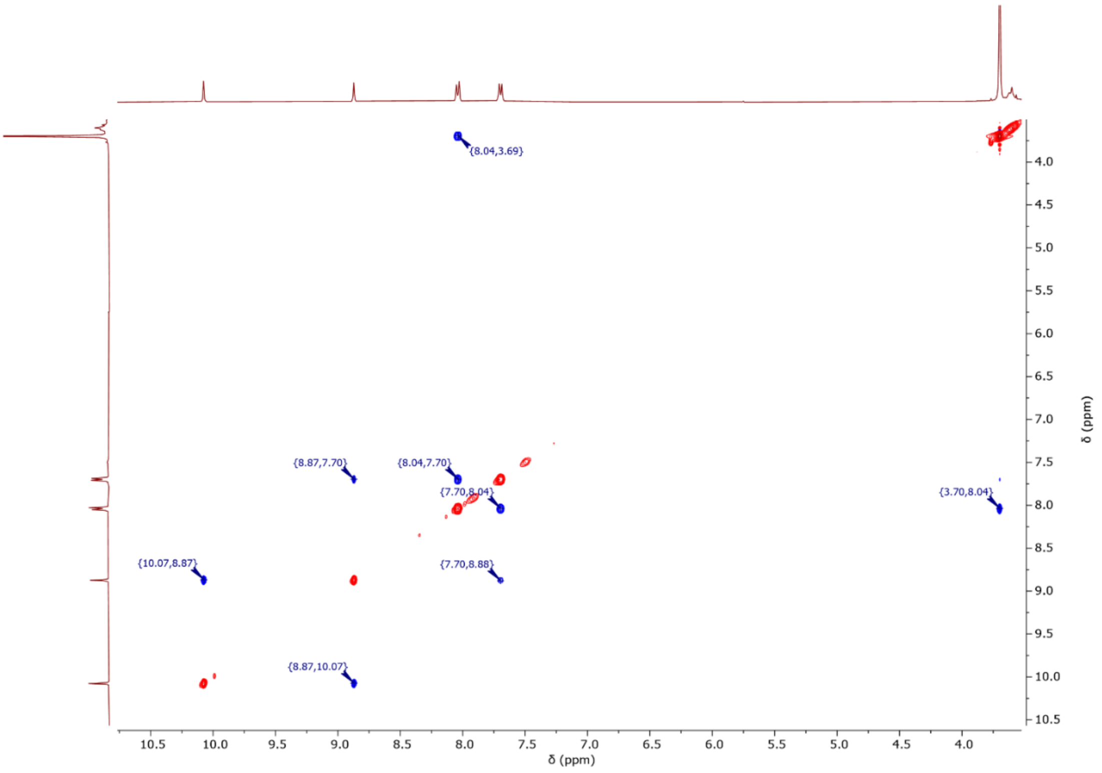


**Fig. S26** $^{1}H$-$^{1}H$-ROESY-NMR spectrum (400 MHz, DMSO-$d_6$) of **Zn-NMe$_3$Nc**.

## Section S2: Mass spectra of OMe, NMe$_2$, Zn-OMeNc, Zn-NMe$_2$Nc, Zn-NMe$_3$Nc

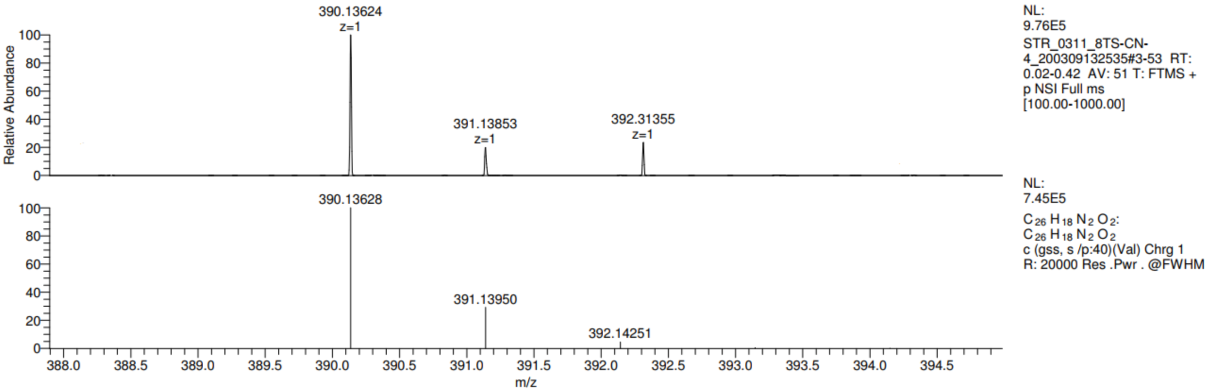


**Fig. S27** EM-MS-ESI spectrum of **OMe**.

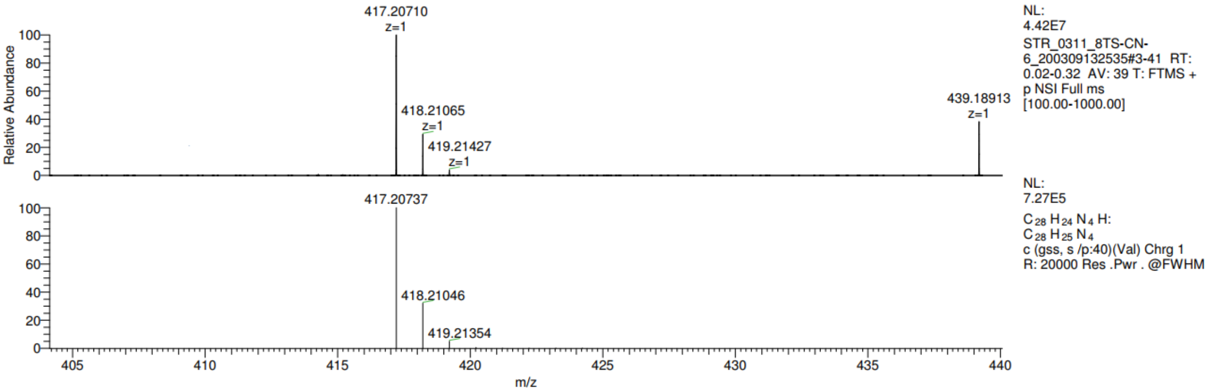


**Fig. S28** EM-MS-ESI spectrum of **NMe$_2$**.

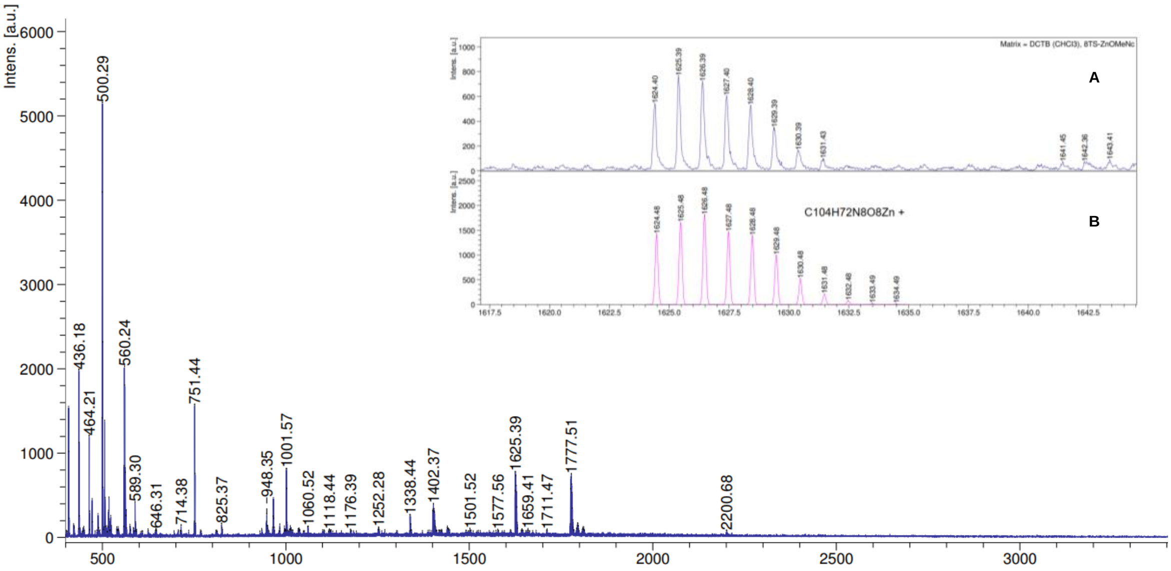


**Fig. S29** MALDI-TOF mass spectrum of **Zn-OMeNc**, isotopic patterns for the molecular ion (inset A) and simulated MS patterns of the molecular ion (inset B).

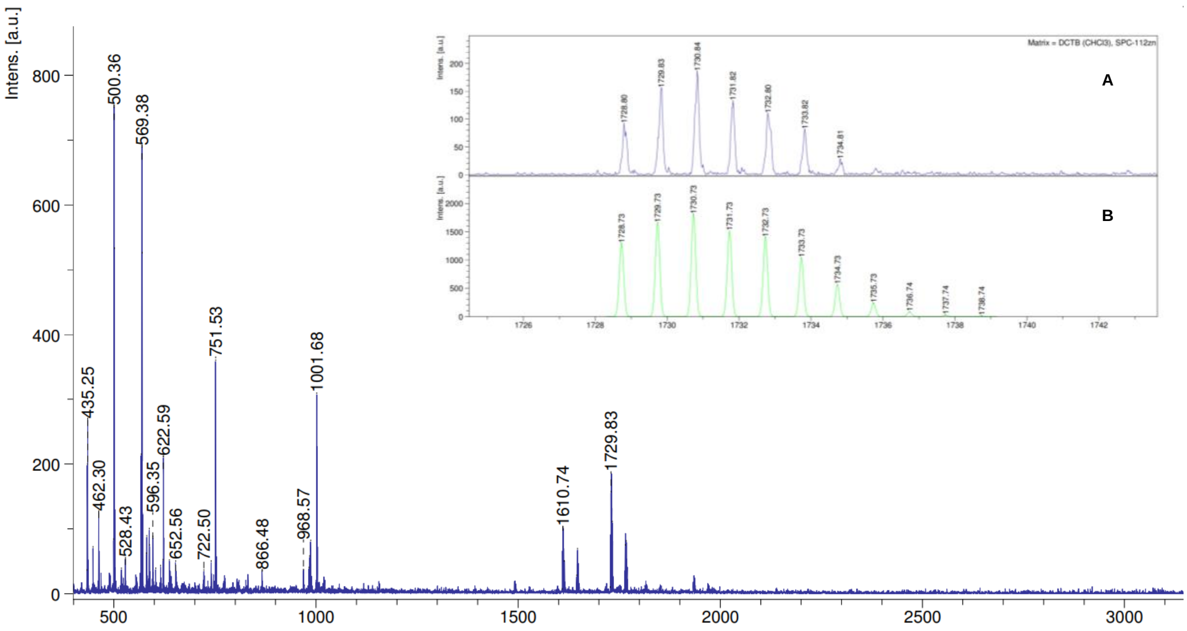


**Fig. S30** MALDI-TOF mass spectrum of **Zn-NMe$_2$Nc**, isotopic patterns for the molecular ion (inset A) and simulated MS patterns of the molecular ion (inset B).

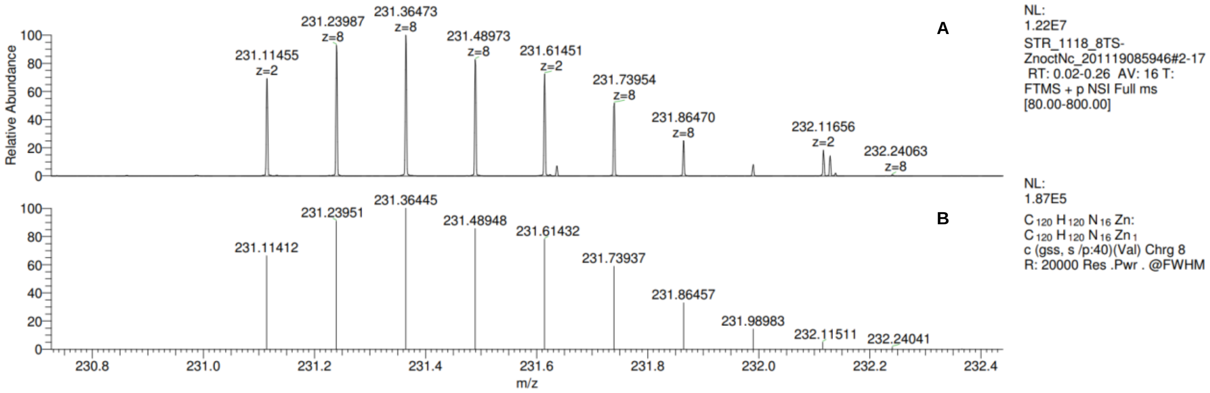


**Fig. S31** EM-MS-ESI spectrum of **Zn-NMe$_3$Nc**, isotopic patterns for the molecular ion (inset A) and simulated MS patterns of the molecular ion (inset B).

## Section S3: Photophysical characterization of Zn-OMeNc, Zn-NMe₂Nc, Zn-NMe₃Nc

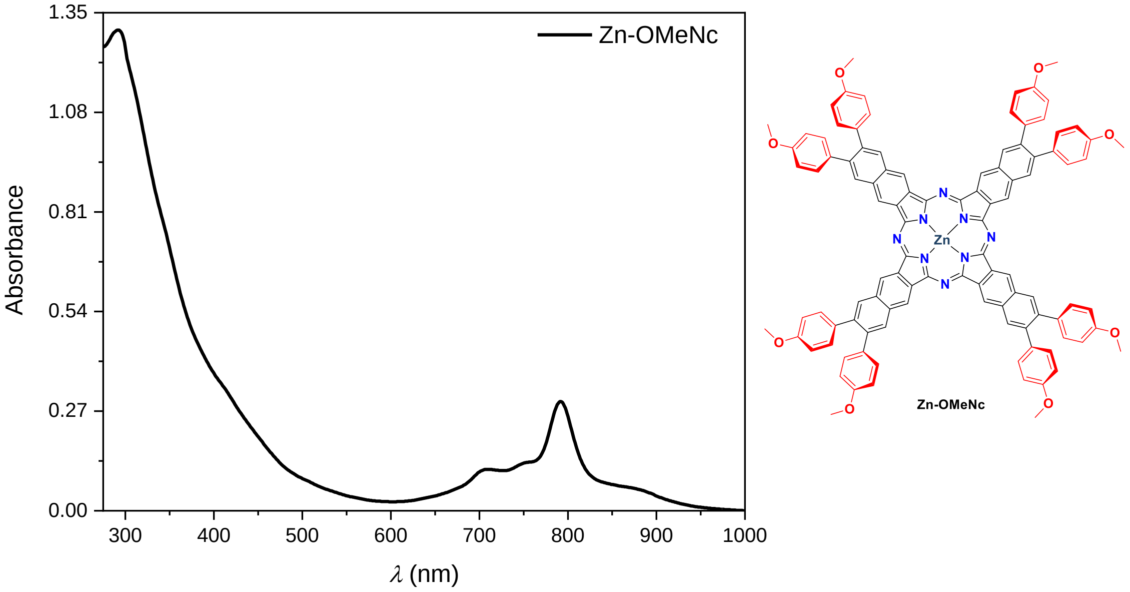


**Fig. S32** UV-vis absorption spectrum of **Zn-OMeNc** in liquid DMSO at 298 K ($\lambda_{max}$ = 794 nm).

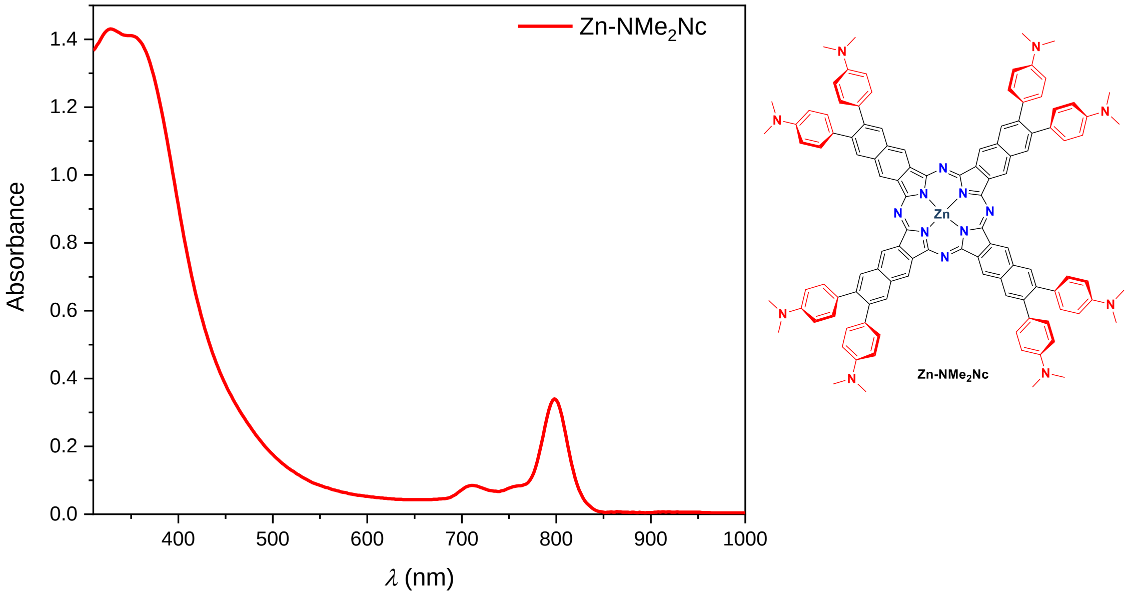


**Fig. S33** UV-vis absorption spectrum of **Zn-NMe₂Nc** in liquid DMSO at 298 K ($\lambda_{max}$ = 800 nm).

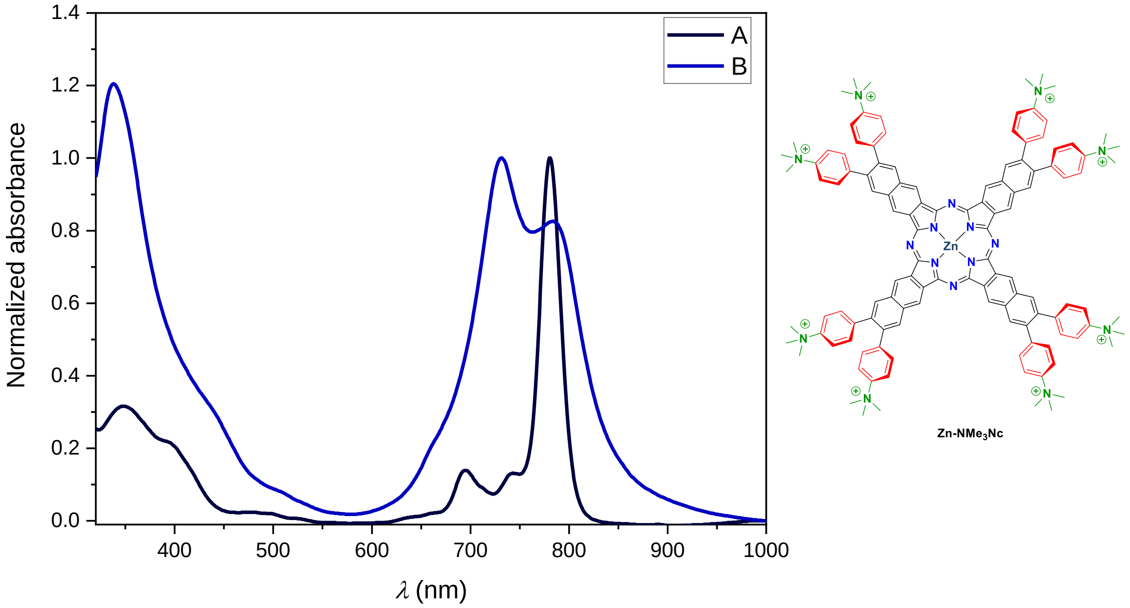


**Fig. S34** UV-vis absorption spectra of **Zn-NMe₃Nc** in liquid DMSO (A) ($\lambda_{max}$ = 781 nm) and water (B) ($\lambda_{max}$ = 732, 784 nm) at 298 K.

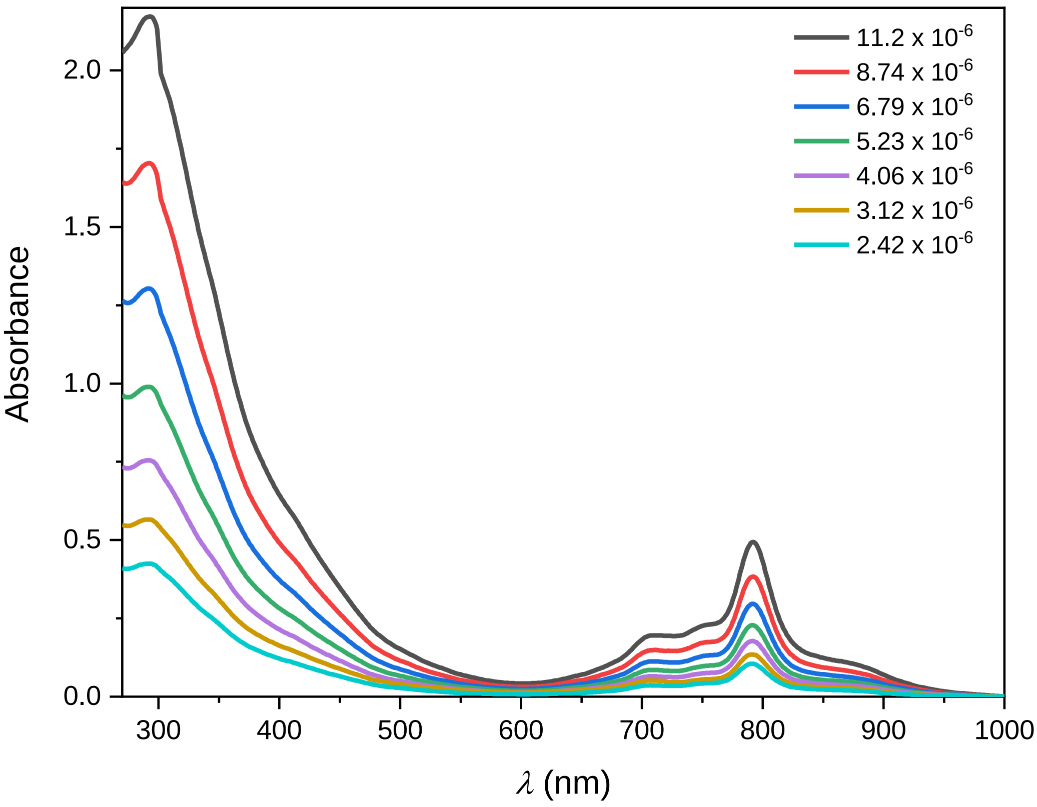


**Fig. S35** UV-vis absorption spectra of **Zn-OMeNc** in liquid DMSO at different concentrations.

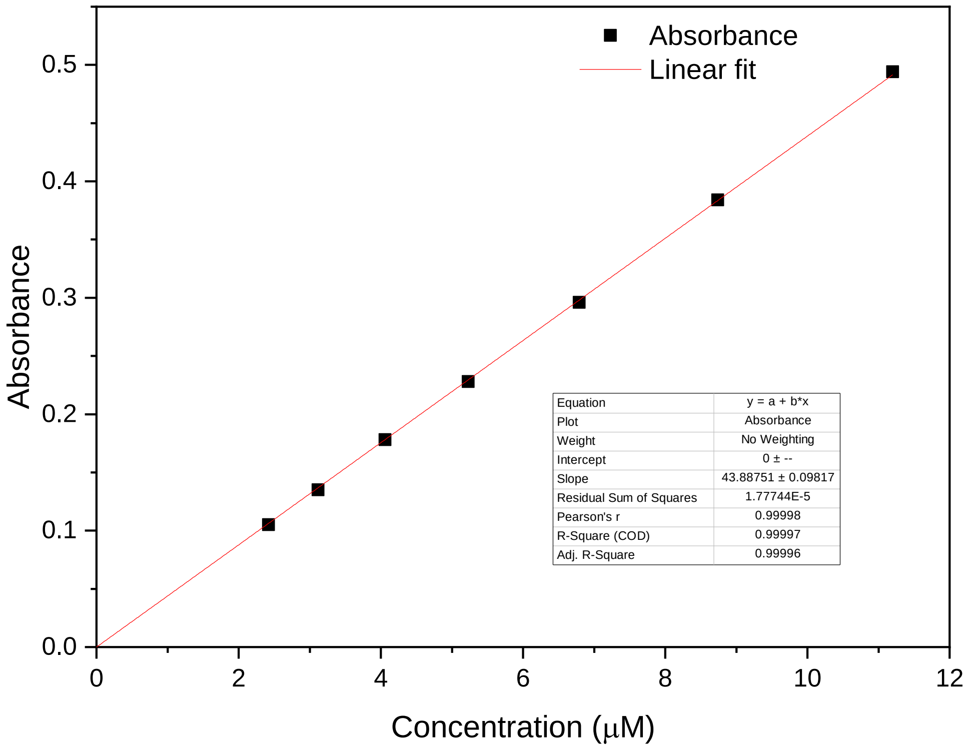


**Fig. S36** Lambert-Beer law verified for **Zn-OMeNc** in liquid DMSO at 298 K ($\lambda_{max}$ = 794 nm).

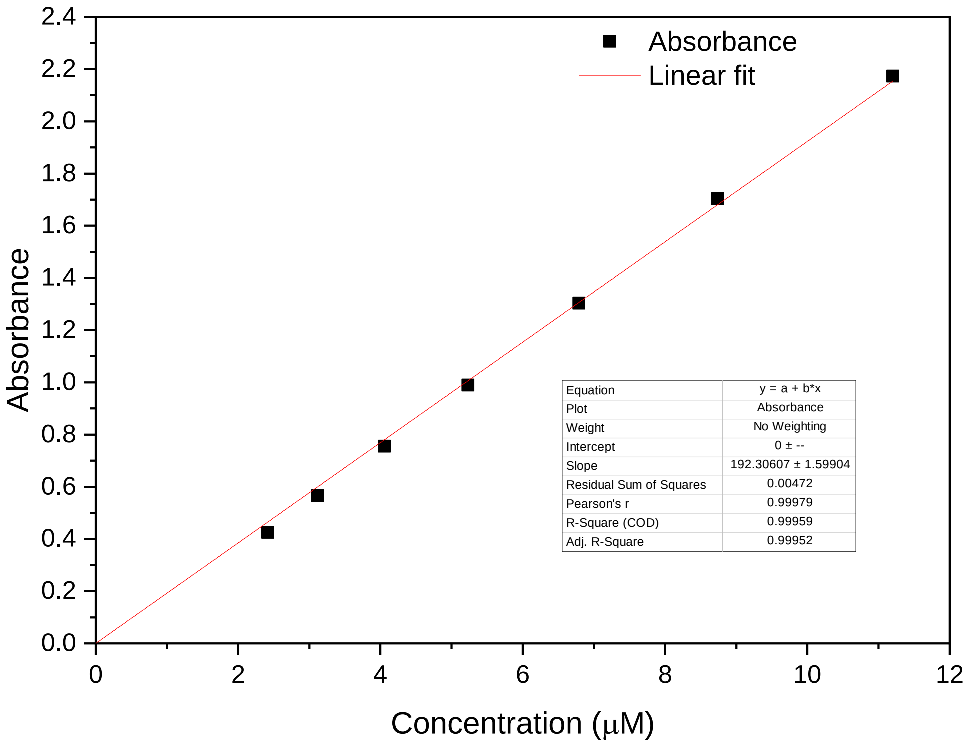


**Fig. S37** Lambert-Beer law verified for **Zn-OMeNc** in liquid DMSO at 298 K ($\lambda_{max}$ = 293 nm).

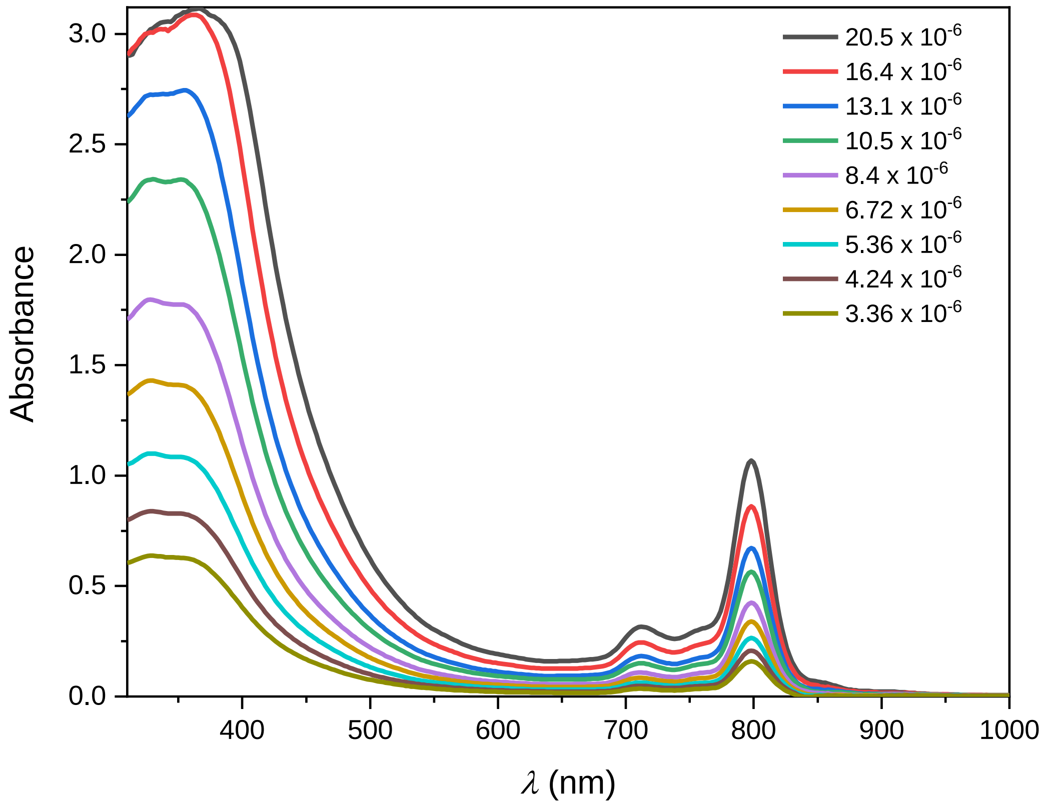


**Fig. S38** UV-vis absorption spectra of **Zn-NMe$_2$Nc** in liquid DMSO at different concentrations.

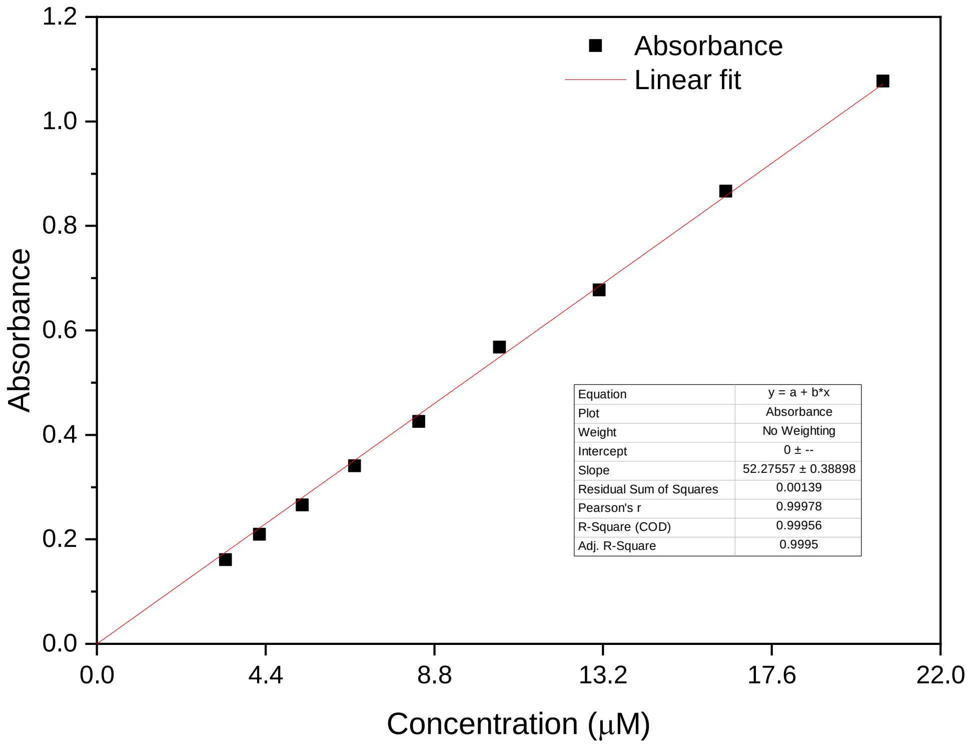


**Fig. S39** Lambert-Beer law verified for **Zn-NMe$_2$Nc** in liquid DMSO at 298 K ($\lambda_{max}$ = 800 nm).

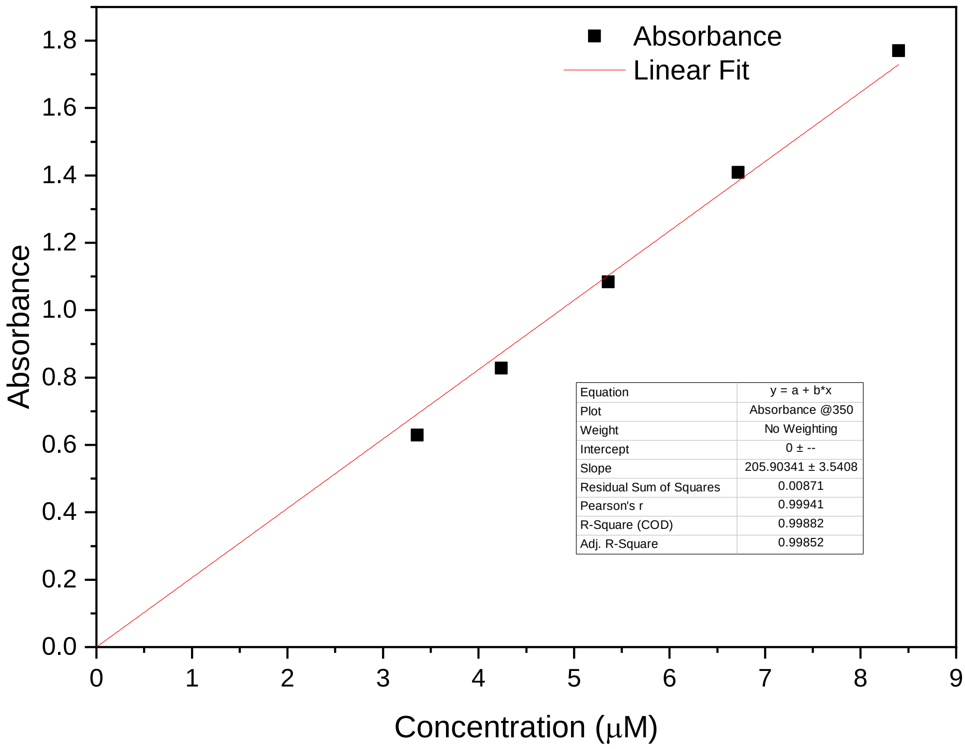


**Fig. S40** Lambert-Beer law verified for **Zn-NMe₂Nc** in liquid DMSO at 298 K ($\lambda_{max}$ = 350 nm).

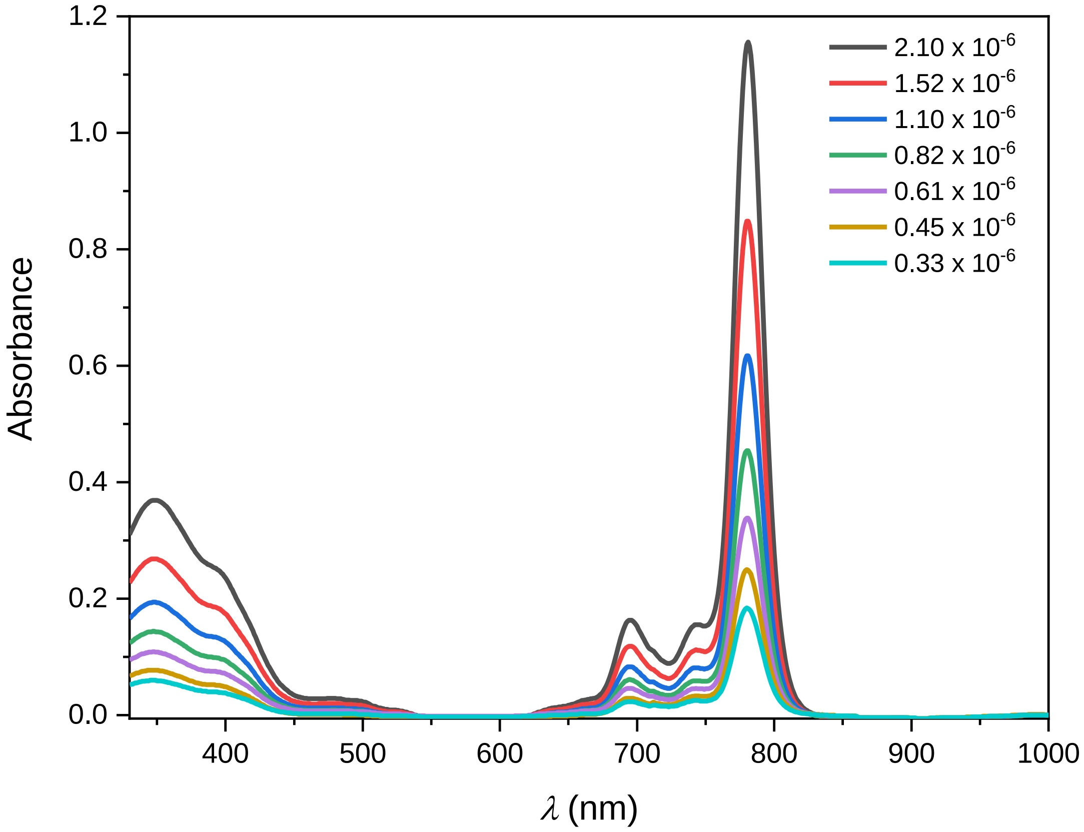


**Fig. S41** UV-vis absorption spectra of **Zn-NMe₃Nc** in liquid DMSO at different concentrations.

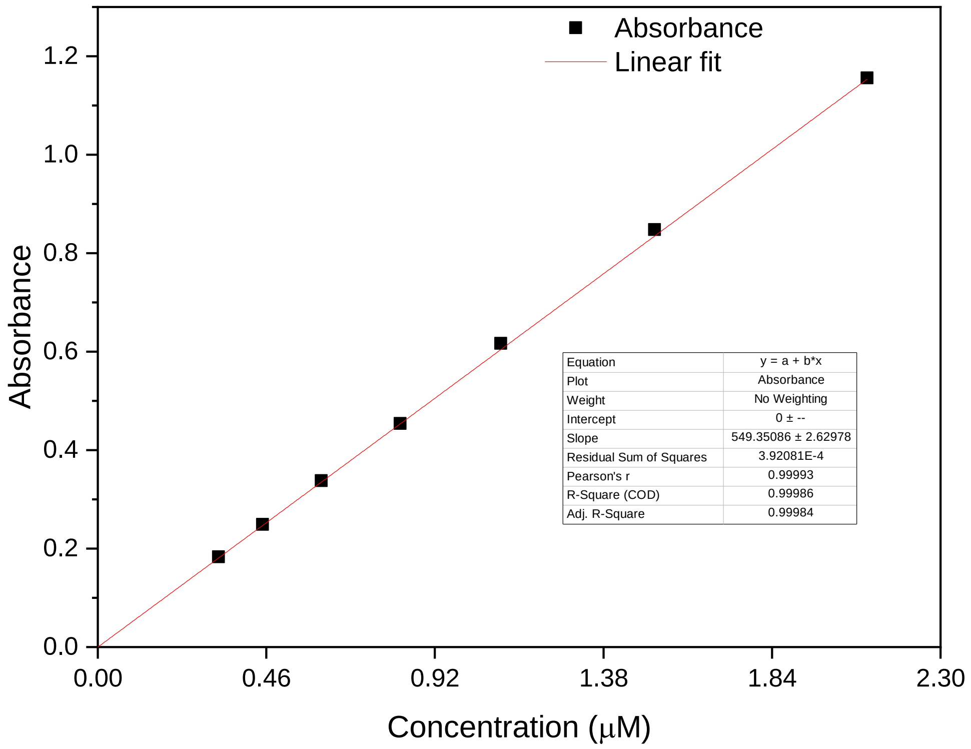


**Fig. S42** Lambert-Beer law verified for **Zn-NMe$_3$Nc** liquid DMSO at 298 K ($\lambda_{max}$ = 781 nm).

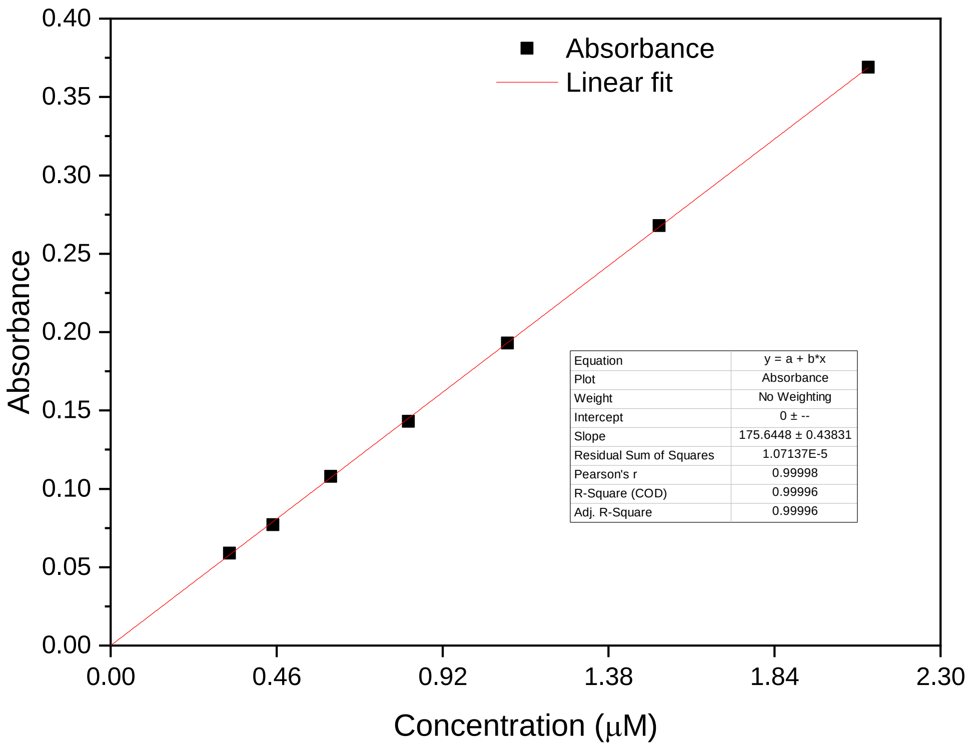


**Fig. S43** Lambert-Beer law verified for **Zn-NMe$_3$Nc** liquid DMSO at 298 K ($\lambda_{max}$ = 350 nm).

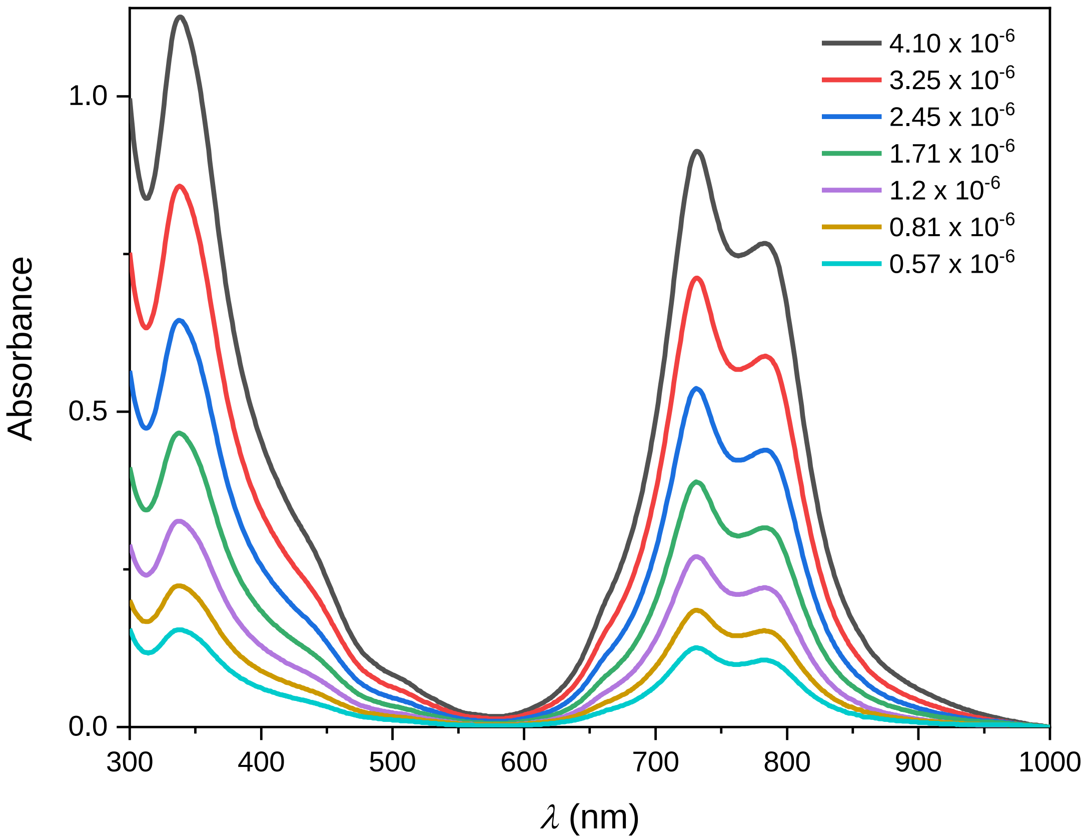


**Fig. S44** UV-vis absorption spectra of **Zn-NMe₃Nc** in liquid water at different concentrations.

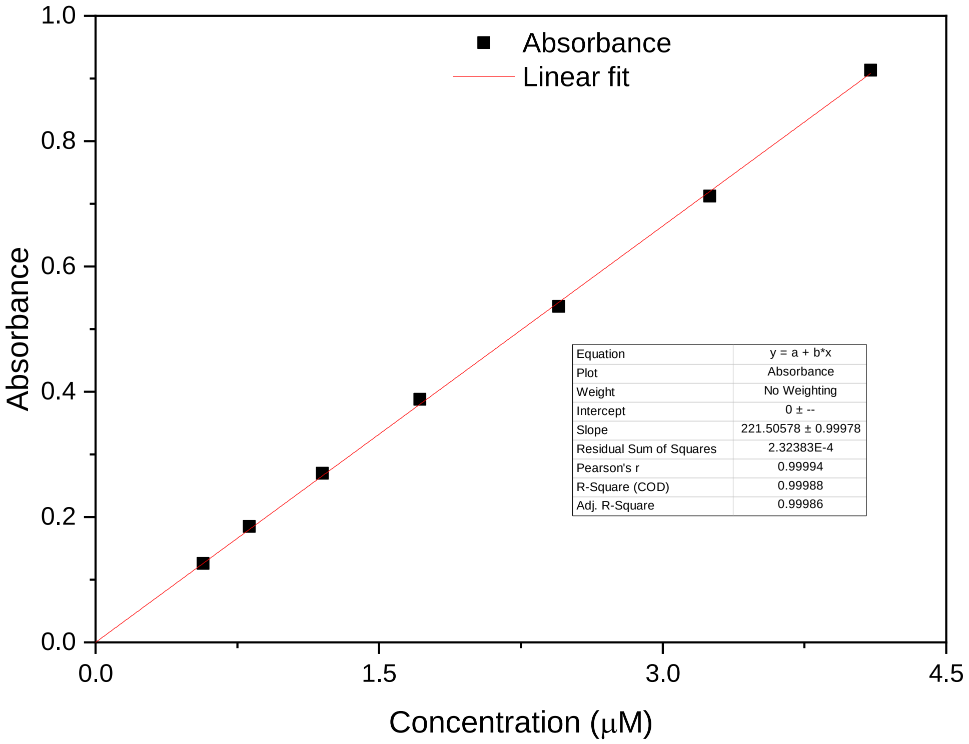


**Fig. S45** Lambert-Beer law verified for **Zn-NMe$_3$Nc** in liquid water at 298 K ($\lambda_{max}$ = 732 nm).

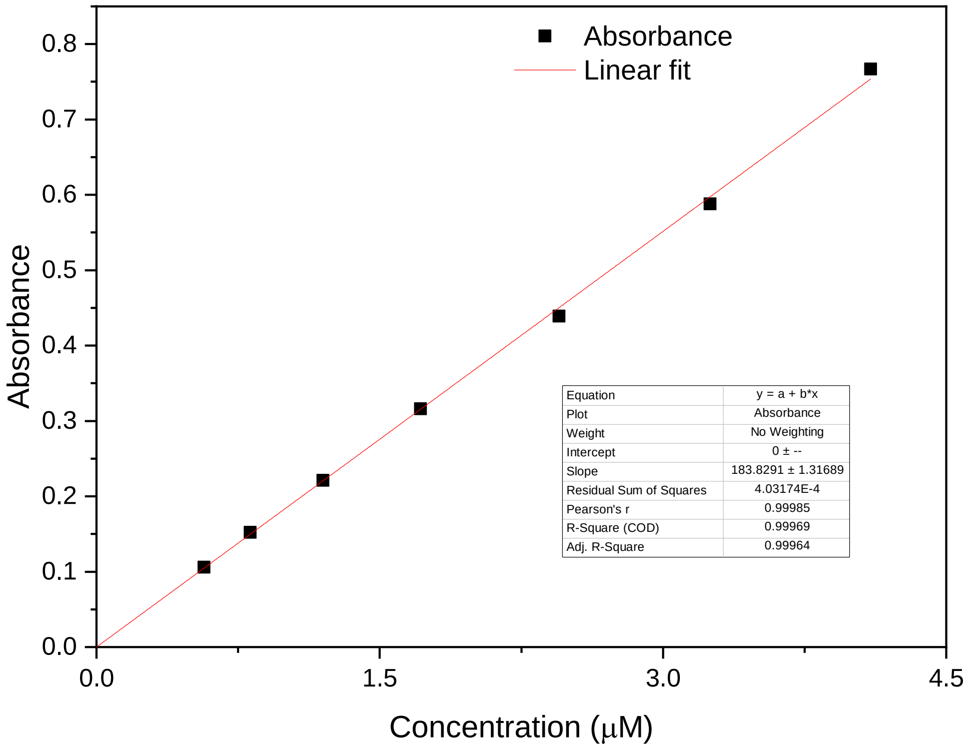


**Fig. S46** Lambert-Beer law verified for **Zn-NMe$_3$Nc** in liquid water at 298 K ($\lambda_{max}$ = 784 nm).

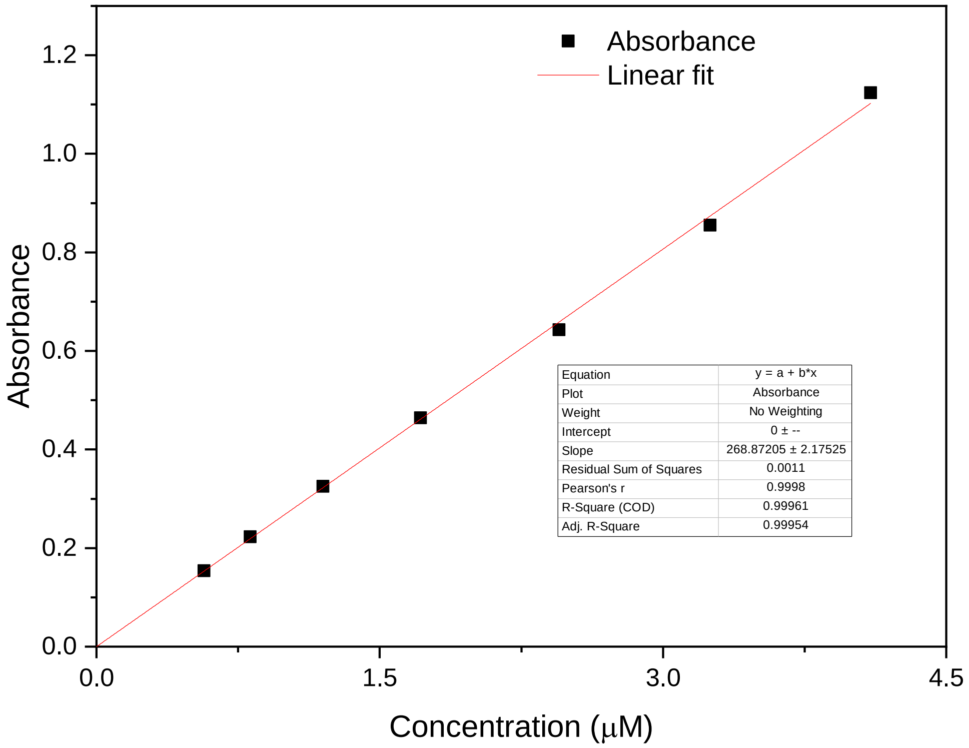


**Fig. S47** Lambert-Beer law verified for **Zn-NMe$_3$Nc** in liquid water at 298 K ($\lambda_{max}$ = 340 nm).

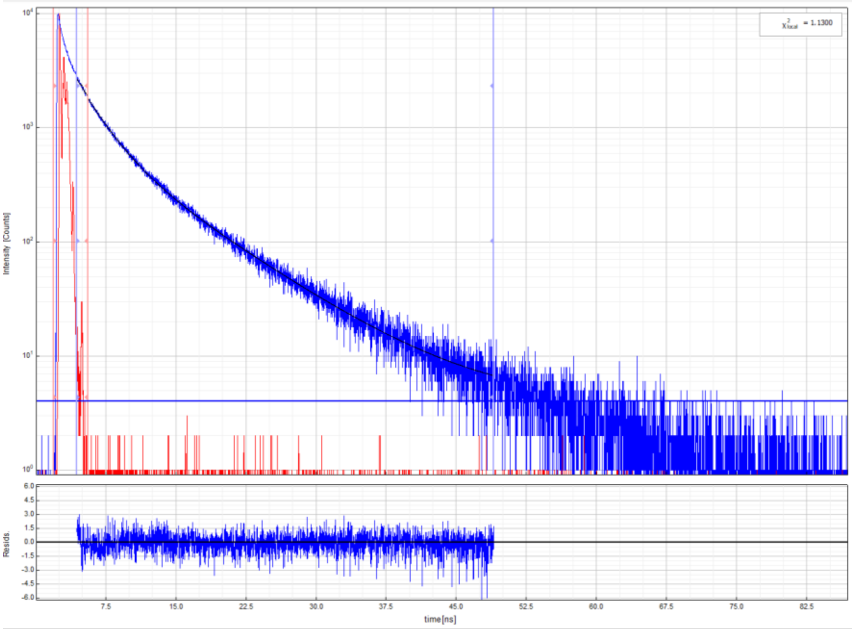


| Parameter | Value | Error |
|---|---|---|
| $A_1$[kCnts/Chnl] | 0.972 | ± 0.067 |
| $\tau_1$[ns] | 8.15 | ± 0.25 |
| $I_1$[kCnts] | 495 | ± 22 |
| $A_{Rel\,1}$[%] | 20.9 | ± 1.3 |
| $I_{Rel\,1}$[%] | 44.4 | ± 1.9 |
| $A_2$[kCnts/Chnl] | 3.68 | ± 0.20 |
| $\tau_2$[ns] | 2.69 | ± 0.11 |
| $I_2$[kCnts] | 619 | ± 40 |
| $A_{Rel\,2}$[%] | 79.1 | ± 1.3 |
| $I_{Rel\,2}$[%] | 55.6 | ± 1.9 |
| $Bkgr_{Dec}$[kCnts] | 0.0035 | ± 0.0006 |
| $Bkgr_{IRF}$[Cnts/Chnl] | 97 | ± 21 |
| $Shift_{IRF}$[ps] | 262 | ± 94 |
| $A_{Scatt}$[kCnts] | -110 | ± 64 |
| $\tau_{Av\,Int}$[ns] | 5.119 | ± 0.091 |
| $\tau_{Av\,Amp}$[ns] | 3.833 | ± 0.093 |

**Fig. S48** Left: Raw (experimental) time-resolved photoluminescence decay of **ZnOMeNc** in liquid DMSO (blue, $c = 10^{-5}$ M) and the instrument response function (red) at 298 K, including the residuals ($\lambda_{ex}$ = 376 nm, $\lambda_{em}$ = 508 nm). Right: Fitting parameters including pre-exponential factors and confidence limits.

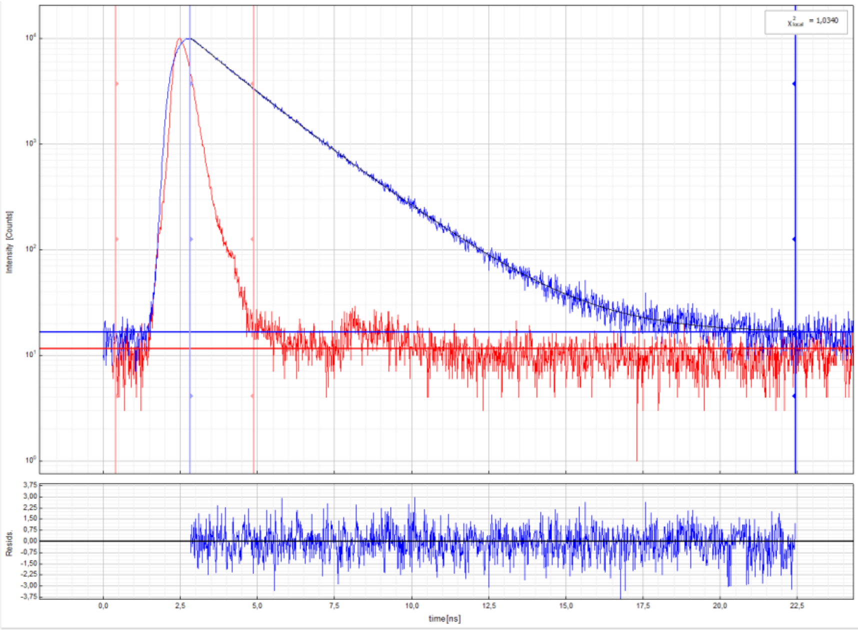


| Parameter | Value | Error |
|---|---|---|
| $A_1$[kCnts/Chnl] | 11,54 | ± 0,36 |
| $\tau_1$[ns] | 2,052 | ± 0,025 |
| $I_1$[kCnts] | 1480 | ± 28 |
| $A_{Rel\,1}$[%] | 68,4 | ± 2,9 |
| $I_{Rel\,1}$[%] | 81,6 | ± 1,7 |
| $A_2$[kCnts/Chnl] | 5,32 | ± 0,74 |
| $\tau_2$[ns] | 1,00 | ± 0,15 |
| $I_2$[kCnts] | 334 | ± 34 |
| $A_{Rel\,2}$[%] | 31,6 | ± 2,9 |
| $I_{Rel\,2}$[%] | 18,4 | ± 1,7 |
| $Bkgr_{Dec}$[kCnts] | 0,0164 | ± 0,0004 |
| $Bkgr_{IRF}$[Cnts/Chnl] | 5 | ± 23 |
| $Shift_{IRF}$[ps] | -228 | ± 16 |
| $A_{Scatt}$[kCnts] | -34 | ± 177 |
| $\tau_{Av\,Int}$[ns] | 1,859 | ± 0,039 |
| $\tau_{Av\,Amp}$[ns] | 1,721 | ± 0,085 |

**Fig. S49** Left: Raw (experimental) time-resolved photoluminescence decay of **ZnOMeNc** in liquid DMSO (blue, $c = 10^{-5}$ M) and the instrument response function (red) at 298 K, including the residuals ($\lambda_{ex}$ = 376 nm, $\lambda_{em}$ = 810 nm). Right: Fitting parameters including pre-exponential factors and confidence limits.

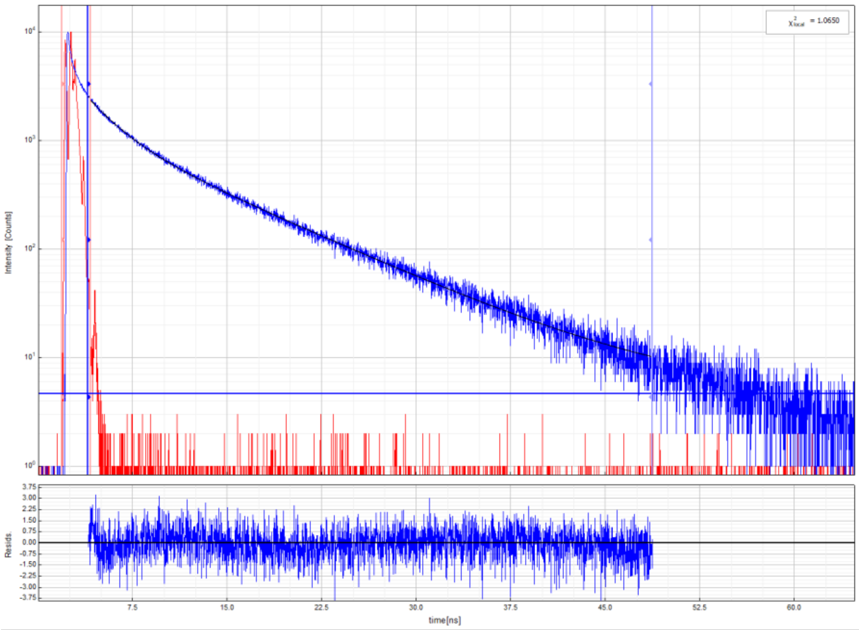


| Parameter | Value | Error |
|---|---|---|
| $A_1$[kCnts/Chnl] | 1.70 | ± 0.14 |
| $\tau_1$[ns] | 8.37 | ± 0.18 |
| $I_1$[kCnts] | 888 | ± 77 |
| $A_{Rel\,1}$[%] | 38.73 | ± 0.90 |
| $I_{Rel\,1}$[%] | 70.0 | ± 1.6 |
| $A_2$[kCnts/Chnl] | 2.69 | ± 0.27 |
| $\tau_2$[ns] | 2.27 | ± 0.15 |
| $I_2$[kCnts] | 381 | ± 43 |
| $A_{Rel\,2}$[%] | 61.27 | ± 0.90 |
| $I_{Rel\,2}$[%] | 30.0 | ± 1.6 |
| $Bkgr_{Dec}$[kCnts] | 0.0047 | ± 0.0006 |
| $Bkgr_{IRF}$[Cnts/Chnl] | -201 | ± 188 |
| $Shift_{IRF}$[ps] | -166 | ± 31 |
| $A_{Scatt}$[kCnts] | 118 | ± 365 |
| $\tau_{Av\,Int}$[ns] | 6.537 | ± 0.068 |
| $\tau_{Av\,Amp}$[ns] | 4.63 | ± 0.12 |

**Fig. S50** Left: Raw (experimental) time-resolved photoluminescence decay of **Zn-NMe2Nc** in liquid DMSO (blue, *c* = 10-5 M) and the instrument response function (red) at 298 K, including the residuals ($\lambda_{ex}$ = 376 nm, $\lambda_{em}$ = 560 nm). Right: Fitting parameters including pre-exponential factors and confidence limits.

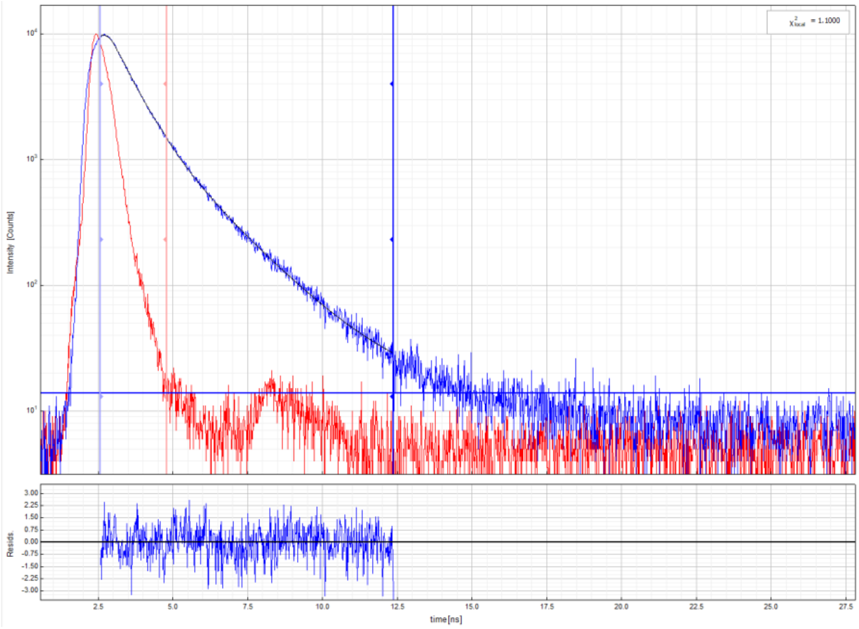


| Parameter | Value | Error |
|---|---|---|
| $A_1$[kCnts/Chnl] | 3.26 | ± 0.33 |
| $\tau_1$[ns] | 1.801 | ± 0.083 |
| $I_1$[kCnts] | 367 | ± 22 |
| $A_{Rel\,1}$[%] | 21.0 | ± 2.0 |
| $I_{Rel\,1}$[%] | 43.8 | ± 2.4 |
| $A_2$[kCnts/Chnl] | 12.31 | ± 0.50 |
| $\tau_2$[ns] | 0.614 | ± 0.032 |
| $I_2$[kCnts] | 472 | ± 18 |
| $A_{Rel\,2}$[%] | 79.0 | ± 2.0 |
| $I_{Rel\,2}$[%] | 56.2 | ± 2.4 |
| $Bkgr_{Dec}$[kCnts] | 0.0138 | ± 0.0046 |
| $Bkgr_{IRF}$[Cnts/Chnl] | -40 | ± 43 |
| $Shift_{IRF}$[ps] | 250.0000 | |
| $A_{Scatt}$[kCnts] | 133 | ± 14 |
| $\tau_{Av\,Int}$[ns] | 1.133 | ± 0.027 |
| $\tau_{Av\,Amp}$[ns] | 0.863 | ± 0.038 |

**Fig. S51** Left: Raw (experimental) time-resolved photoluminescence decay of **Zn-NMe2Nc** in liquid DMSO (blue, *c* = $10^{-5}$ M) and the instrument response function (red) at 298 K, including the residuals ($\lambda_{ex}$ = 376 nm, $\lambda_{em}$ = 811 nm). Right: Fitting parameters including pre-exponential factors and confidence limits.

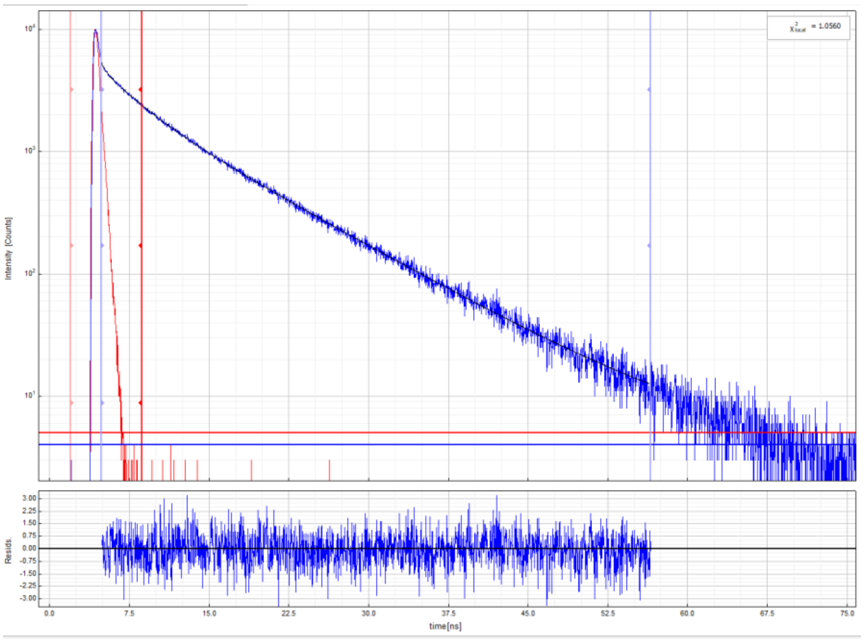


| Parameter | Value | Error |
|---|---|---|
| $A_1$[kCnts/Chnl] | 2.91 | ± 0.13 |
| $\tau_1$[ns] | 9.06 | ± 0.21 |
| $I_1$[kCnts] | 824 | ± 20 |
| $A_{Rel\,1}$[%] | 55.4 | ± 2.7 |
| $I_{Rel\,1}$[%] | 76.7 | ± 3.0 |
| $A_2$[kCnts/Chnl] | 2.34 | ± 0.16 |
| $\tau_2$[ns] | 3.42 | ± 0.42 |
| $I_2$[kCnts] | 250 | ± 40 |
| $A_{Rel\,2}$[%] | 44.6 | ± 2.7 |
| $I_{Rel\,2}$[%] | 23.3 | ± 3.0 |
| $Bkgr_{Dec}$[kCnts] | 0.0034 | ± 0.0010 |
| $Bkgr_{IRF}$[Cnts/Chnl] | 36 | ± 33 |
| $Shift_{IRF}$[ps] | -32 | ± 37 |
| $A_{Scat}$[kCnts] | 107 | ± 12 |
| $\tau_{Av\,Int}$[ns] | 7.748 | ± 0.062 |
| $\tau_{Av\,Amp}$[ns] | 6.55 | ± 0.20 |

**Fig. S52** Left: Raw (experimental) time-resolved photoluminescence decay of **Zn-NMe3Nc** in liquid DMSO (blue, $c = 10^{-5}$ M) and the instrument response function (red) at 298 K, including the residuals ($\lambda_{ex} = 376$ nm, $\lambda_{em} = 550$ nm). Right: Fitting parameters including pre-exponential factors and confidence limits.

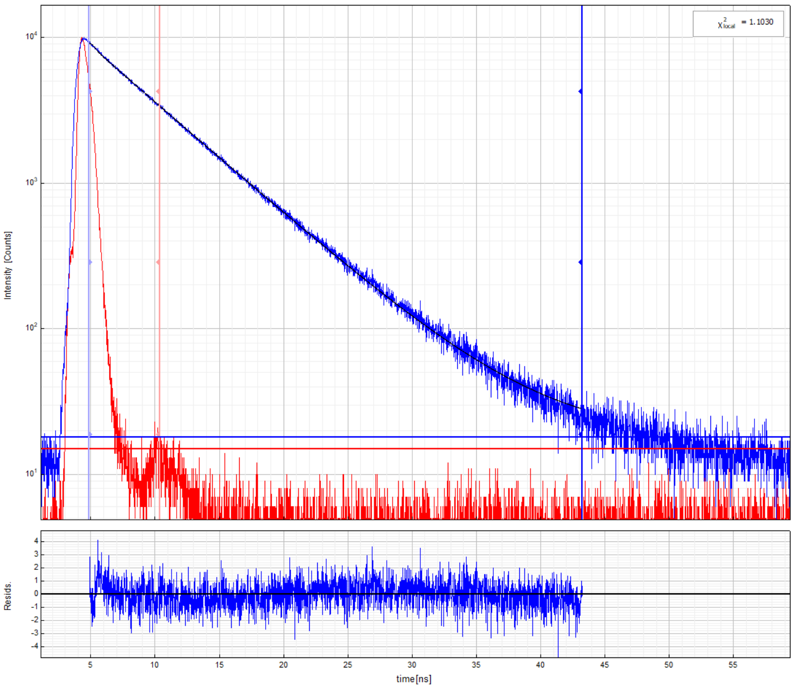


| Parameter | Value | Error |
|---|---|---|
| $A_1$[kCnts/Chnl] | 9.526 | ± 0.029 |
| $\tau_1$[ns] | 5.689 | ± 0.016 |
| $I_1$[kCnts] | 3386.8 | ± 6.6 |
| $A_{Rel\,1}$[%] | 100.0000 | ± 0.0000 |
| $I_{Rel\,1}$[%] | 100.0000 | ± 0.0000 |
| $Bkgr_{Dec}$[kCnts] | 0.0148 | ± 0.0004 |
| $Bkgr_{IRF}$[Cnts/Chnl] | 15.0000 | |
| $Shift_{IRF}$[ps] | 500.0000 | |
| $A_{Scat}$[kCnts] | 272.6 | ± 9.2 |
| $\tau_{Av\,Int}$[ns] | 5.689 | ± 0.016 |
| $\tau_{Av\,Amp}$[ns] | 5.689 | ± 0.016 |

**Fig. S53** Left: Raw (experimental) time-resolved photoluminescence decay of **Zn-NMe3Nc** in liquid DMSO (blue, $c = 10^{-5}$ M) and the instrument response function (red) at 298 K, including the residuals ($\lambda_{ex} = 376$ nm, $\lambda_{em} = 801$ nm). Right: Fitting parameters including pre-exponential factors and confidence limits.

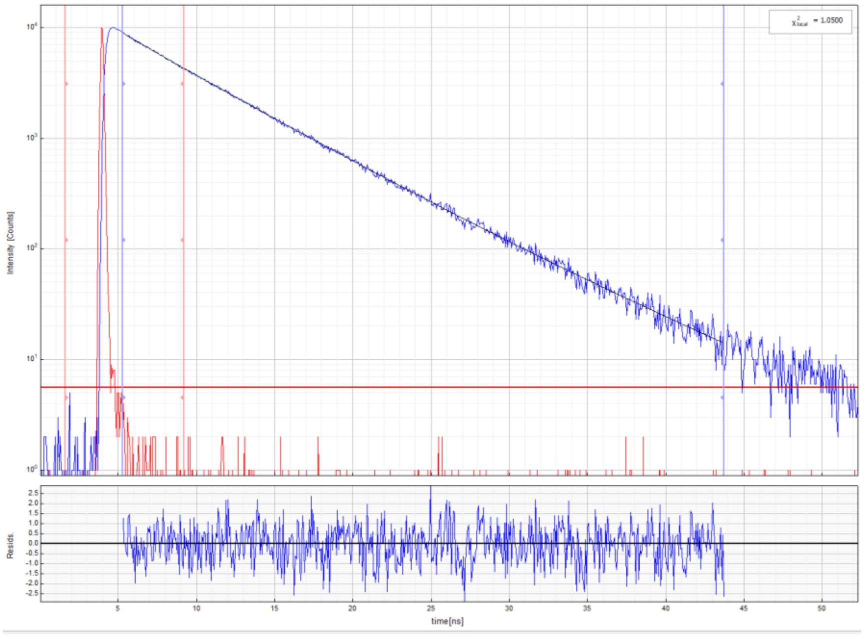


| Parameter | Value | Error |
|---|---|---|
| $A_1$[kCnts/Chnl] | 1.06 | ± 0.15 |
| $\tau_1$[ns] | 8.49 | ± 0.44 |
| $I_1$[kCnts] | 140 | ± 17 |
| $A_{Rel\,1}$[%] | 9.1 | ± 1.2 |
| $I_{Rel\,1}$[%] | 14.1 | ± 1.7 |
| $A_2$[kCnts/Chnl] | 10.53 | ± 0.20 |
| $\tau_2$[ns] | 5.209 | ± 0.070 |
| $I_2$[kCnts] | 857 | ± 24 |
| $A_{Rel\,2}$[%] | 90.9 | ± 1.2 |
| $I_{Rel\,2}$[%] | 85.9 | ± 1.7 |
| $Bkgr_{Dec}$[kCnts] | -0.0003 | ± 0.0020 |
| $Bkgr_{IRF}$[Cnts/Chnl] | 5.6 | ± 2.6 |
| $Shift_{IRF}$[ps] | -162 | ± 34 |
| $A_{Scat}$[kCnts] | 203 | ± 208 |
| $\tau_{Av\,Int}$[ns] | 5.670 | ± 0.023 |
| $\tau_{Av\,Amp}$[ns] | 5.508 | ± 0.030 |

**Fig. S54** Left: Raw (experimental) time-resolved photoluminescence decay of **Zn-NMe$_3$Nc** in liquid $H_2O$ (blue, $c$ = $10^{-5}$ M) and the instrument response function (red) at 298 K, including the residuals ($\lambda_{ex}$ = 376 nm, $\lambda_{em}$ = 432 nm). Right: Fitting parameters including pre-exponential factors and confidence limits.

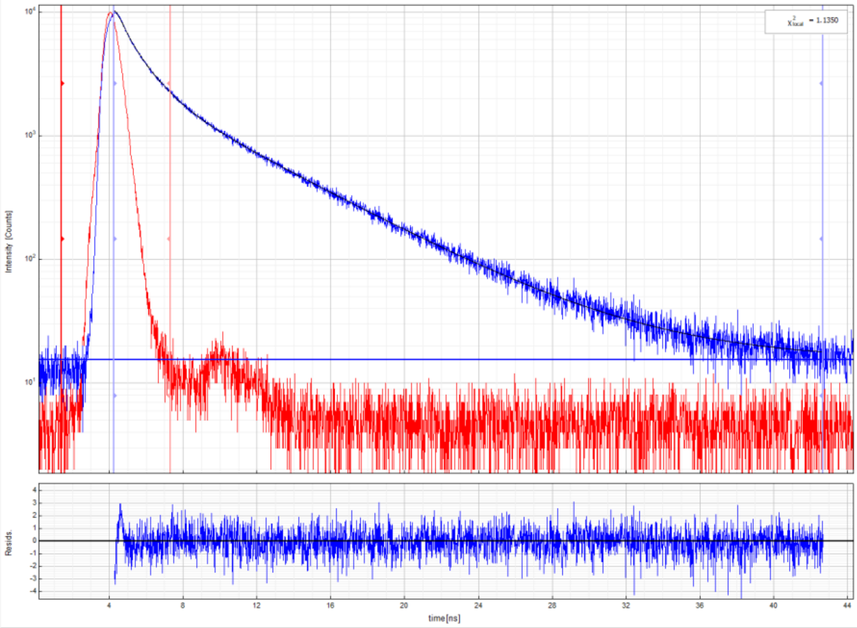


| Parameter | Value | Error |
|---|---|---|
| $A_1$[kCnts/Chnl] | 2.919 | ± 0.060 |
| $\tau_1$[ns] | 5.432 | ± 0.051 |
| $I_1$[kCnts] | 991 | ± 19 |
| $A_{Rel\,1}$[%] | 28.5 | ± 1.2 |
| $I_{Rel\,1}$[%] | 64.5 | ± 1.2 |
| $A_2$[kCnts/Chnl] | 7.32 | ± 0.51 |
| $\tau_2$[ns] | 1.192 | ± 0.079 |
| $I_2$[kCnts] | 546 | ± 37 |
| $A_{Rel\,2}$[%] | 71.5 | ± 1.2 |
| $I_{Rel\,2}$[%] | 35.5 | ± 1.2 |
| $Bkgr_{Dec}$[kCnts] | 0.0154 | ± 0.0006 |
| $Bkgr_{IRF}$[Cnts/Chnl] | -10 | ± 33 |
| $Shift_{IRF}$[ps] | 91 | ± 66 |
| $A_{Scat}$[kCnts] | 355 | ± 22 |
| $\tau_{Av\,Int}$[ns] | 3.927 | ± 0.054 |
| $\tau_{Av\,Amp}$[ns] | 2.40 | ± 0.12 |

**Fig. S55** Left: Raw (experimental) time-resolved photoluminescence decay of **Zn-NMe$_3$Nc** in liquid $H_2O$ (blue, $c$ = $10^{-5}$ M) and the instrument response function (red) at 298 K, including the residuals ($\lambda_{ex}$ = 376 nm, $\lambda_{em}$ = 804 nm). Right: Fitting parameters including pre-exponential factors and confidence limits.

## Section S4: DFT calculations

**Table S1.** Monomer emission wavelengths (in nm) for the $S_1 \rightarrow S_0$ and $S_3 \rightarrow S_0$ transitions as obtained from the difference in energy between the corresponding optimized singlet geometries obtained with CAM-B3LYP functional.

| | **Zn-NMe₂Nc ($\lambda_{max}$ / nm)** | **Zn-OMeNc ($\lambda_{max}$ / nm)** | **Zn-NMe₃Nc ($\lambda_{max}$ / nm)** |
|---|---|---|---|
| $S_1$ (0-0) | 839.40 | 828.20 | 817.60 |
| $S_1$ (vertical) | 888.20 | 874.90 | 861.70 |
| $S_3$ (0-0) | 450.50 | 390.40 | 393.90 |
| $S_3$ (vertical) | 503.00 | 397.10 | 392.90 |

**Table S2.** Monomer emission wavelengths (in nm) for the $S_1 \rightarrow S_0$ and $S_3 \rightarrow S_0$ transitions as obtained from the difference in energy between the corresponding optimized singlet geometries obtained with M06-2X functional.

| | **Zn-NMe₂Nc ($\lambda_{max}$ / nm)** | **Zn-OMeNc ($\lambda_{max}$ / nm)** | **Zn-NMe₃Nc ($\lambda_{max}$ / nm)** |
|---|---|---|---|
| $S_1$ (0-0) | 816.60 | 802.90 | 792.80 |
| $S_1$ (vertical) | 888.20 | 874.90 | 861.70 |
| $S_3$ (0-0) | 479.40 | 393.70 | 397.80 |
| $S_3$ (vertical) | 503.40 | 397.10 | 392.90 |

**Table S3. TDDFT output for the optimized $S_1$ state with the PBE0 functional.**

**Zn-NMe$_2$Nc:**

```
Excited State   1:      Singlet    1.3859 eV   894.60 nm   f=1.5426  <S**2>=0.000
     450 -> 451       -0.70315

 Excited State   2:      Singlet    1.5712 eV   789.13 nm   f=1.3333  <S**2>=0.000
     450 -> 452       -0.69721

 Excited State   3:      Singlet    1.7517 eV   707.81 nm   f=0.0100  <S**2>=0.000
     446 -> 451        0.62707
     447 -> 451       -0.29216
     448 -> 451       -0.11923
```

**Zn-OMeNc:**

Excited State 1: Singlet 1.4583 eV 850.21 nm f=1.4799 <S**2>=0.000
418 -> 419 0.70495

Excited State 2: Singlet 1.6333 eV 759.12 nm f=1.3475 <S**2>=0.000
418 -> 420 0.70296

Excited State 3: Singlet 2.5219 eV 491.63 nm f=0.0012 <S**2>=0.000
416 -> 419 -0.63757
417 -> 419 0.26909

**Zn-Nme$_3$Nc:**

Excited State 1: Singlet 1.4946 eV 829.57 nm f=1.4806 <S**2>=0.000
482 -> 483 0.70479

Excited State 2: Singlet 1.6640 eV 745.10 nm f=1.3553 <S**2>=0.000
482 -> 484 -0.70290

Excited State 3: Singlet 2.6522 eV 467.48 nm f=0.0001 <S**2>=0.000
482 -> 485 0.70323

**Table S4. TDDFT output for the optimized S$_3$ state with the PBE0 functional.**

**Zn-NMe$_2$Nc:**

Excited State 1: Singlet 1.3854 eV 894.91 nm f=1.5975 <S**2>=0.000
450 -> 451 0.70308

Excited State 2: Singlet 1.5737 eV 787.86 nm f=1.1410 <S**2>=0.000
449 -> 451 0.18743
450 -> 452 0.67370

Excited State 3: Singlet 1.6277 eV 761.73 nm f=0.2369 <S**2>=0.000
449 -> 451 0.67946
450 -> 452 -0.18547

Excited State 4: Singlet 1.7639 eV 702.90 nm f=0.0656 <S**2>=0.000
446 -> 451 -0.70249

Excited State 5: Singlet 1.7858 eV 694.26 nm f=0.1647 <S**2>=0.000
448 -> 451 -0.70360

**Zn-OMeNc:**

```
Excited State   1:      Singlet   1.4392 eV  861.48 nm  f=1.4669  <S**2>=0.000
     418 -> 419         0.70502
 Excited State   2:      Singlet   1.6550 eV  749.15 nm  f=1.4042  <S**2>=0.000
     418 -> 420         0.70253

 Excited State   3:      Singlet   2.2385 eV  553.88 nm  f=0.1551  <S**2>=0.000
     417 -> 419         0.70275

 Excited State   4:      Singlet   2.4568 eV  504.66 nm  f=0.1417  <S**2>=0.000
     413 -> 419         0.18122
     417 -> 420         0.67214

 Excited State   5:      Singlet   2.5027 eV  495.39 nm  f=0.1582  <S**2>=0.000
     413 -> 419         0.62512
     415 -> 419        -0.18271
     416 -> 419         0.17749
     417 -> 420        -0.16789
```

**Zn-Nme$_3$Nc:**

```
Excited State   1:      Singlet   1.5755 eV  786.93 nm  f=1.4257  <S**2>=0.000
     482 -> 483        -0.70393

 Excited State   2:      Singlet   1.5781 eV  785.66 nm  f=1.4059  <S**2>=0.000
     482 -> 484        -0.70393

 Excited State   3:      Singlet   2.6031 eV  476.29 nm  f=0.0002  <S**2>=0.000
     482 -> 485         0.70369

 Excited State   4:      Singlet   2.7217 eV  455.54 nm  f=0.0002  <S**2>=0.000
     482 -> 486        -0.70132

 Excited State   5:      Singlet   2.7975 eV  443.20 nm  f=0.1784  <S**2>=0.000
     482 -> 487         0.70167
```

## Section S5: ZnNMe$_2$Nc loaded onto polystyrene microparticles (PSMPs)

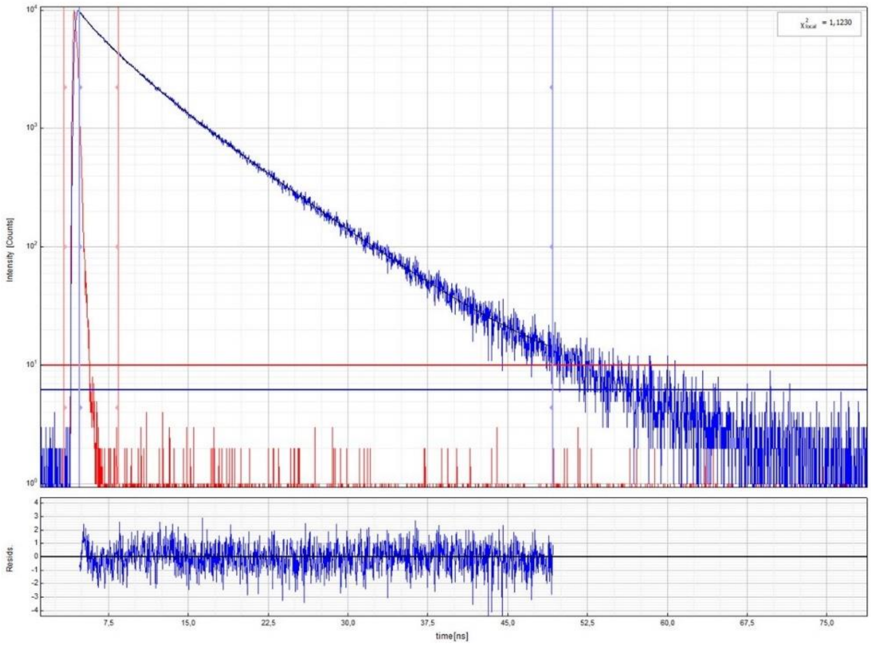


| Parameter | Value | Error |
|---|---|---|
| $A_1$[kCnts/Chnl] | 5,84 | ± 0,31 |
| $\tau_1$[ns] | 6,81 | ± 0,12 |
| $I_1$[kCnts] | 1244 | ± 46 |
| $A_{Rel\,1}$[%] | 56,2 | ± 2,7 |
| $I_{Rel\,1}$[%] | 75,0 | ± 2,7 |
| $A_2$[kCnts/Chnl] | 4,55 | ± 0,25 |
| $\tau_2$[ns] | 2,91 | ± 0,15 |
| $I_2$[kCnts] | 414 | ± 44 |
| $A_{Rel\,2}$[%] | 43,8 | ± 2,7 |
| $I_{Rel\,2}$[%] | 25,0 | ± 2,7 |
| $Bkgr_{Dec}$[kCnts] | 0,0063 | ± 0,0012 |
| $Bkgr_{IRF}$[Cnts/Chnl] | 10,0000 | |
| $Shift_{IRF}$[ps] | -6,0047 | |
| $A_{Scatt}$[kCnts] | 95,3 | ± 8,8 |
| $\tau_{Av\,Int}$[ns] | 5,839 | ± 0,021 |
| $\tau_{Av\,Amp}$[ns] | 5,105 | ± 0,033 |

**Figure S56** Left: Raw (experimental) time-resolved photoluminescence decay of **Zn-NMe$_2$Nc@PSMP** in $H_2O$ (blue) and the instrument response function (red) at 298 K, including the residuals ($\lambda_{ex}$ = 376 nm, $\lambda_{em}$ = 475 nm). Right: Fitting parameters including pre-exponential factors and confidence limits.

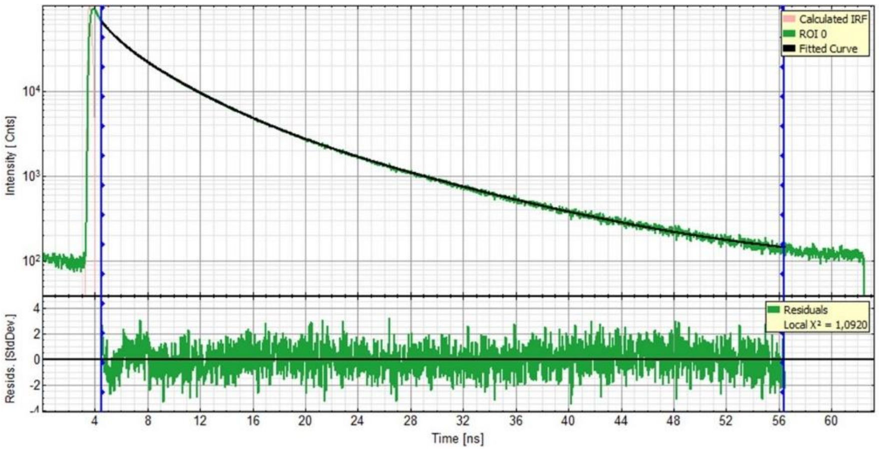


| Parameter | | Data Set (3) |
|---|---|---|
| $\tau_1$[ns] | Limits | 10,100 ± 0,073 |
| $\tau_2$[ns] | Limits | 3,670 ± 0,031 |
| $\tau_3$[ns] | Limits | 1,090 ± 0,024 |
| $Bkgr_{Dec}$[Cnts] | Limits | 88,0 ± 2,4 |
| $I_1$[kCnts] | | 4911 ± 47 |
| $I_2$[kCnts] | | 6814 ± 69 |
| $I_3$[kCnts] | | 1032 ± 35 |
| $I_{Sum}$[kCnts] | | 12757,0 ± 8,8 |
| $A_{Sum}$[kCnts] | | 66,00 ± 0,15 |
| $\tau_{Av\,Int}$[ns] | | 5,950 ± 0,019 |
| $\tau_{Av\,Amp}$[ns] | | 3,870 ± 0,011 |
| $A_{rel\,1}$ | | 0,147 ± 0,002 |
| $A_{rel\,2}$ | | 0,564 ± 0,004 |
| $A_{rel\,3}$ | | 0,2889 ± 0,0036 |

**Figure S57** Left: Raw (experimental) time-resolved photoluminescence decay of **Zn-NMe$_2$Nc@PSMP** observed under the confocal microscope (SPE) and the instrument response function (red) at 298 K, including the residuals ($\lambda_{ex}$ = 376 nm, 460 LP filter). Right: Fitting parameters including pre-exponential factors and confidence limits.

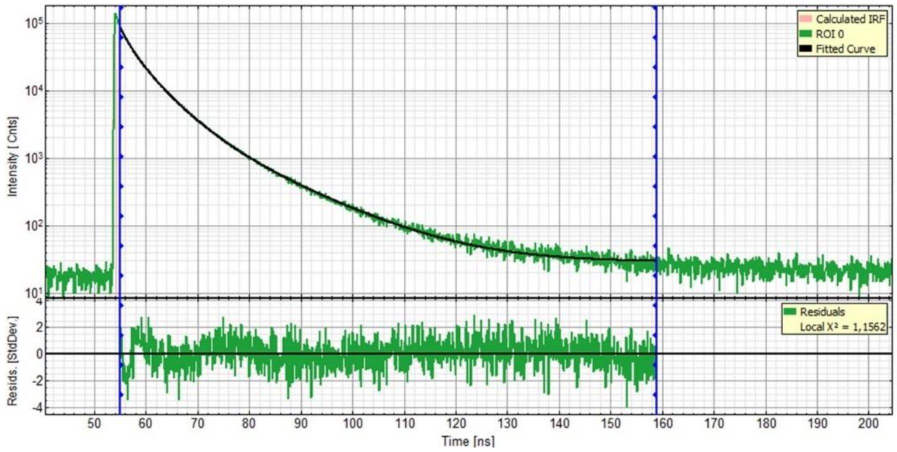


| Parameter | | Overall Decay |
| --- | --- | --- |
| $t_0$[ns] | | 54,8800 ± ------ |
| $A_1$[kCnts] | Limits | 48,00 ± 0,25 |
| $A_2$[kCnts] | Limits | 6,600 ± 0,078 |
| $A_3$[kCnts] | Limits | 39,00 ± 0,19 |
| $\tau_1$[ns] | Limits | 4,630 ± 0,016 |
| $\tau_2$[ns] | Limits | 12,000 ± 0,052 |
| $\tau_3$[ns] | Limits | 1,720 ± 0,031 |
| $Bkgr_{Dec}$[Cnts] | Limits | 65 ± 1 |
| $I_1$[kCnts] | | 2798,0 ± 8,2 |
| $I_2$[kCnts] | | 992,0 ± 8,3 |
| $I_3$[kCnts] | | 835 ± 16 |
| $I_{Sum}$[kCnts] | | 4625,0 ± 1,9 |
| $A_{Sum}$[kCnts] | | 94,00 ± 0,13 |
| $\tau_{Av Int}$[ns] | | 5,6920 ± 0,0034 |
| $\tau_{Av Amp}$[ns] | | 3,9430 ± 0,0065 |

**Figure S58** Left: Raw (experimental) time-resolved photoluminescence decay of **Zn-NMe$_2$Nc@PSNP** observed under the confocal microscope (TPE) and the instrument response function (red) at 298 K, including the residuals ($\lambda_{ex}$ = 810 nm, 460 LP filter). Right: Fitting parameters including pre-exponential factors and confidence limits.
